# Genome-Wide Identification and Expression Profile Analysis of the Phenylalanine Ammonia-Lyase Gene Family in *Hevea brasiliensis*

**DOI:** 10.3390/ijms25095052

**Published:** 2024-05-06

**Authors:** Hui Liu, Qiguang He, Yiyu Hu, Ruilin Lu, Shuang Wu, Chengtian Feng, Kun Yuan, Zhenhui Wang

**Affiliations:** Key Laboratory of Biology and Genetic Resources of Rubber Tree, Ministry of Agriculture and Rural Affairs/State Key Laboratory Incubation Base for Cultivation & Physiology of Tropical Crops, Rubber Research Institute, Chinese Academy of Tropical Agricultural Sciences, Haikou 571101, China; hqg11300817@163.com (Q.H.); huyy2009@163.com (Y.H.); 17689786590@163.com (R.L.); 17863609652@163.com (S.W.); fengchengtian@126.com (C.F.); yuankun__628@126.com (K.Y.)

**Keywords:** rubber tree, phenylpropanoid metabolism, phenylalanine ammonia-lyase (PAL), hormone, abiotic stress, biotic stress, tapping panel dryness

## Abstract

The majority of the world’s natural rubber comes from the rubber tree (*Hevea brasiliensis*). As a key enzyme for synthesizing phenylpropanoid compounds, phenylalanine ammonia-lyase (PAL) has a critical role in plant satisfactory growth and environmental adaptation. To clarify the characteristics of rubber tree PAL family genes, a genome-wide characterization of rubber tree *PALs* was conducted in this study. Eight *PAL* genes (*HbPAL1*-*HbPAL8*), which spread over chromosomes 3, 7, 8, 10, 12, 13, 14, 16, and 18, were found to be present in the genome of *H. brasiliensis*. Phylogenetic analysis classified HbPALs into groups I and II, and the group I HbPALs (HbPAL1-HbPAL6) displayed similar conserved motif compositions and gene architectures. Tissue expression patterns of *HbPALs* quantified by quantitative real-time PCR (qPCR) proved that distinct *HbPALs* exhibited varying tissue expression patterns. The *HbPAL* promoters contained a plethora of *cis*-acting elements that responded to hormones and stress, and the qPCR analysis demonstrated that abiotic stressors like cold, drought, salt, and H_2_O_2_-induced oxidative stress, as well as hormones like salicylic acid, abscisic acid, ethylene, and methyl jasmonate, controlled the expression of *HbPALs*. The majority of *HbPALs* were also regulated by powdery mildew, anthracnose, and *Corynespora* leaf fall disease infection. In addition, *HbPAL1*, *HbPAL4*, and *HbPAL7* were significantly up-regulated in the bark of tapping panel dryness rubber trees relative to that of healthy trees. Our results provide a thorough comprehension of the characteristics of *HbPAL* genes and set the groundwork for further investigation of the biological functions of *HbPALs* in rubber trees.

## 1. Introduction

In the phenylpropanoid biosynthesis pathway, phenylalanine ammonia-lyase (PAL; EC 4.3.1.24) is the first committed enzyme. It is responsible for catalyzing L-phenylalanine conversion to *trans*-cinnamate, the starting point for the biosynthesis of various phenylpropanoid compounds, like salicylic acid (SA), lignin, flavonoids, isoflavonoids, etc. [1,2]. These metabolites are essential for satisfactory plant growth and environmental adaptation. Many studies have shown that the accumulation of phenylpropanoid compounds was correlated with PAL activity [3,4,5]. *PALs* have been characterized in rice [6], wheat [7], castor [3], *Fritillaria unibracteata* [8], *Astragalus membranaceus* [9], and *Panax ginseng* [10] because of PAL significance for phenylpropanoid biosynthesis.

Studies have shown that there are different numbers of *PAL* gene members in different plant species, for instance, 17 in *Brassica napus* [11], 9 in *Oryza sativa* [12], 5 in *Populus trichocarpa* [13], 7 in *Cucumis sativus* [1], 12 in *Juglans regia* [14], 14 in *Solanum tuberosum* [15], and 54 in *Triticum aestivum* [16]. Various tissues showed distinct expression patterns for different PAL family members [2]. For example, three *SmPALs* were identified in *Salvia miltiorrhiza*. *SmPAL1* and *SmPAL3* displayed relatively high expression levels in roots and leaves, whereas *SmPAL2* was highly expressed in stems and flowers [17]. Furthermore, distinct environmental stimuli, including drought, cold, salt, phosphate shortage, wounding, pathogen infection, SA, and methyl jasmonate (MeJA), controlled *PAL* gene expression in diverse ways [1,12,15,16,17,18,19].

The rubber tree (*Hevea brasiliensis*), belonging to the family of spurge (Euphorbiaceae), is an important tropical tree species. It possesses significant economic value due to the production of industrial raw material, natural rubber [20]. Rubber trees grown in non-traditional growing areas (such as China, Laos, Thailand, Vietnam, and Cambodia) are frequently threatened by environmental stress. Although genome-wide characterization of the *PAL* gene family has been implemented in many plant species, little is reported regarding the *PALs* in rubber trees. To date, only a partial cDNA encoding PAL was reported to be induced by SA in the rubber tree [21]. To explore the functions of the *HbPAL* gene family in response to stresses, a thorough and methodical examination of the rubber tree’s *PAL* family genes was conducted, and eight *HbPAL* genes (*HbPAL1*-*HbPAL8*) were identified. Subsequently, a thorough bioinformatics analysis of *HbPALs* was performed, encompassing gene structure, phylogenetic relationship, chromosomal location, promoter *cis*-acting element, and conserved motifs. Additionally, the expression patterns of *HbPALs* in varied tissues and under different hormones or stress treatments were analyzed. Our results provide important findings for further investigations of the biological roles of *HbPALs* in rubber trees.

## 2. Results

### 2.1. Identification and Characterization of PAL Family Genes in Rubber Tree

The rubber tree genome contained eight *PAL* family members, designated as *HbPAL1*-*HbPAL8* (Table 1). These *HbPALs* encoded polypeptides with lengths varying from 522 to 714 amino acids, with an open reading frame (ORF) length ranging from 1569 to 2145 bp. HbPAL2, HbPAL3, and HbPAL4 had 714 amino acids and were the three largest proteins. HbPAL8, consisting of 522 amino acids, was the smallest PAL protein. The projected isoelectric points of HbPALs varied from 5.85 (HbPAL7) to 8.17 (HbPAL8), while the predicted molecular weights of HbPALs ranged from 57.10 (HbPAL8) to 78.29 kDa (HbPAL7) (Table 1). Based on subcellular localization prediction, the cytoplasm was the location of all eight HbPALs (Table 1). Pairwise sequence comparisons indicated that the identities between two HbPAL proteins were 54.95% to 93.16%. HbPAL5 and HbPAL6 had the highest sequence identity (93.16%), whereas HbPAL4 and HbPAL8 had the lowest sequence identity (54.95%) (Table S1).

Figure S1 displayed the multiple sequence alignment of the HbPAL proteins. All of the HbPAL proteins contained four domains: the inserted shielding domain, the core domain, the MIO (4-methylidene-imidazolone-5-one) domain, and the N-terminal domain. This is in keeping with earlier descriptions of PAL proteins in other plants [1,22,23]. The Ala-Ser-Gly (ASG) tripeptide motif, which was the conserved enzymatic active site of plant PALs, was present within the MIO domain. With the exception of the N-terminal region, which exhibited significant sequence divergence, all eight HbPAL proteins were found to be highly conserved (Figure S1).

### 2.2. Chromosome Location and Phylogenetic Analysis of HbPALs

**Figure 1:**
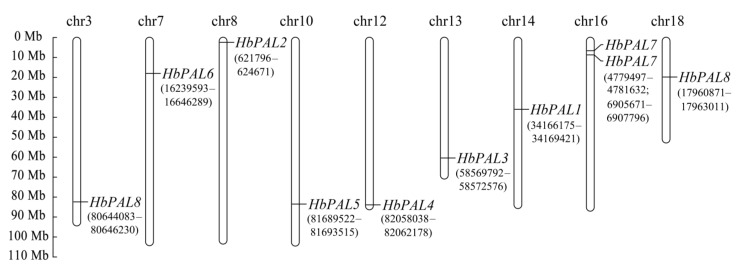
Chromosomal distribution of *HbPAL* genes in *Hevea brasiliensis*.

According to the rubber tree genome annotation, the eight *HbPAL* genes were distributed on nine chromosomes (Figure 1). *HbPAL1* to *HbPAL6* were located on chromosomes 14, 8, 13, 12, 10, and 7, respectively. Notably, *HbPAL7* and *HbPAL8* had two copies. Both copies of *HbPAL7* were found on chromosome 16, while the two copies of *HbPAL8* were localized independently on chromosomes 3 and 18.

**Figure 2:**
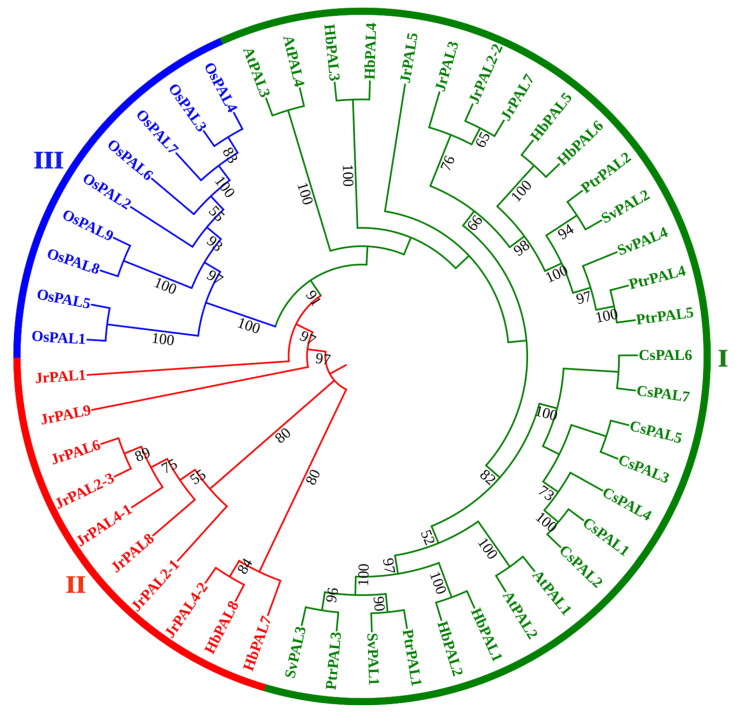
Phylogenetic analysis of PAL proteins from *Oryza sativa* (Os), *Juglans regia* (Jr), *Arabidopsis thaliana* (At), *Salix viminalis* (Sv), *Populus trichocarpa* (Pt), *Cucumis sativus* (Cs), and *Hevea brasiliensis* (Hb). MEGA X was employed for creating the phylogenetic tree, and Evolview was used to visualize the tree. Three groups (I, II, and III) were identified and denoted by distinct colors.

**Figure 3:**
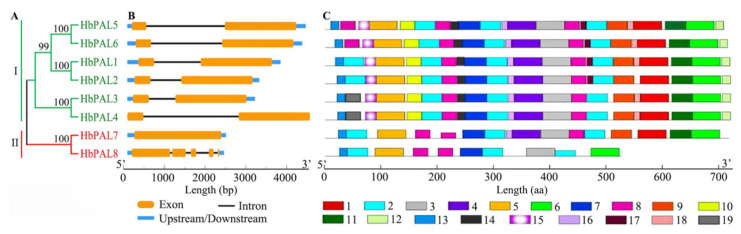
The conserved motifs, gene architectures, and phylogenetic tree of *HbPALs*. (**A**) The *HbPALs*’ evolutionary connection. (**B**) The *HbPALs*’ gene architectures. (**C**) *HbPALs*’ conserved motifs. Figure S2 displays the sequence logos for each motif, which are represented by distinct colored boxes.

To explore the evolutionary relationships among the PAL proteins from different plant species, we constructed a phylogenetic tree using 49 PAL proteins from *O. sativa*, *P. trichocarpa*, *A. thaliana*, *C. sativus*, *J. regia*, *H. brasiliensis*, and *Salix viminalis*. These PALs were grouped into three categories (I, II, and III) (Figure 2). Among them, group I was the largest group and contained thirty PALs, including four AtPALs, four JrPALs, four SvPALs, five PtrPALs, six HbPALs, and seven CsPALs. Group II was the second largest group, consisting of two HbPALs and eight JrPALs. The nine rice OsPALs formed a separate group (III), suggesting that the PALs of monocotyledonous plants were phylogenically different from those of dicotyledonous plants. Furthermore, a phylogenetic tree of HbPALs was also constructed. The eight HbPALs were split into two groups, as seen in Figure 3A, which was consistent with the above clustering result. HbPAL1-HbPAL6 were clustered into group I, and HbPAL7 and HbPAL8 were clustered into group II.

### 2.3. Analysis of Conserved Motif and Gene Structure of HbPALs

To reveal the diversity of gene structure of *HbPALs*, the exon-intron architectures of *HbPALs* were compared according to their phylogenetic relationships. As illustrated in Figure 3A,B, the exon number and length of group I *HbPALs* were very similar. All *HbPALs* of group I (*HbPAL1*-*HbPAL6*) contained two exons. However, the *HbPALs* within group II had quite different exon-intron structures. *HbPAL7* had only one exon, while *HbPAL8* possessed five exons.

To better comprehend the variability in motif compositions among HbPAL proteins, the conserved motifs of HbPALs were identified by the MEME program. Nineteen conserved motifs, numbered 1 through 19, were found (Figure 3C and Figure S2). These motifs’ lengths varied from 10 to 50 amino acids. Motifs 2, 3, 5, 6, 7, 8, and 13 were present in all HbPALs. However, some motifs were exclusively present in group I, including motifs 10, 12, 14, and 15. It is noteworthy that most HbPALs within the same sub-cluster had similar motif compositions, implying that the HbPALs within the same sub-cluster may perform similar functions. Compared to other HbPALs, the HbPAL8 protein was shorter and had fewer motifs, similar to PbPAL3 in *Pyrus bretschneideri* [24].

### 2.4. HbPALs Promoters-Based Cis-acting Element Analysis

**Figure 4:**
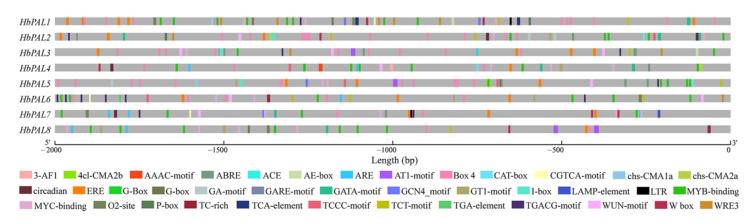
Distribution of the main *cis*-acting elements in *HbPALs* promoters. Different color boxes represent different elements.

In controlling gene expression, *cis*-acting elements located in the promoter region play significant functions. To explore the potential molecular function of *HbPALs*, the 2 kb promoter sequences of *HbPALs* were analyzed with PlantCARE. As shown in Figure 4 and Table S2, numerous important *cis*-acting elements were detected, except for basic elements like TATA-box and CAAT-box. These elements were divided into five main categories. The first type of element was related to light response, including GT1-motif, 3-AF1 binding site, G-box, chs-CMA2a, chs-CMA1a, 4cl-CMA2b, Box II, Box 4, TCT-motif, I-box, MRE, GA-motif, ACE, AAAC-motif, TCCC-motif, Gap-box, AT1-motif, GATA-motif, and AE-box. Of these, only Box 4 was detected in the promoters of all eight *HbPALs*; the others were localized to the promoter regions of specific *HbPALs*. For instance, I-box was found only in the promoters of *HbPAL2*, *HbPAL3*, and *HbPAL5*. The second type of element was involved in hormone response, including MeJA-responsive elements (TGACG-motif and CGTCA-motif), SA-responsive element (TCA-element), gibberellin-responsive elements (P-box and GARE-motif), ABA-responsive elements (ABRE, ABRE3a and ABRE4), ethylene-responsive element (ERE), zein metabolism regulatory element (O2-site), and auxin-responsive element (TGA-element). Among these, the elements involved in MeJA (34), ABA (26), and ethylene (26) accounted for the most number and were separately detected in the promoters of 6, 6, and 8 *HbPALs*. The SA- and auxin-related elements were found only in the promoter of *HbPAL1* and *HbPAL6*, respectively. The O2-site was present in the promoters of *HbPAL1*, *HbPAL5*, *HbPAL6*, and *HbPAL7*, whereas the gibberellin-related elements were found in the promoters of *HbPAL1*, *HbPAL2*, and *HbPAL7*. The third type of element was involved in environmental stress response, including WUN-motif and WRE3 (wound-responsive element), TC-rich repeats (defense and stress responsiveness element), ARE (anaerobic induction regulatory element), LTR (low-temperature responsiveness element), and MBS (MYB binding site involved in drought-inducibility). These elements specifically appeared in the promoters of some *HbPAL* genes. The transcription factor binding sites constituted the fourth category of elements, which included the W boxes, MYC, and MYB. Notably, the promoters of every *HbPAL* gene contained the MYB and MYC binding sites. Plant-specific regulatory elements like circadian (circadian control regulatory element), CAT-box (meristem expression regulatory element), and GCN4_motif (endosperm expression regulatory element) comprised the fifth class of elements. Only a few particular *HbPAL* genes’ promoters contained these elements.

### 2.5. Expression Profiles of HbPALs in Various Tissues

**Figure 5:**
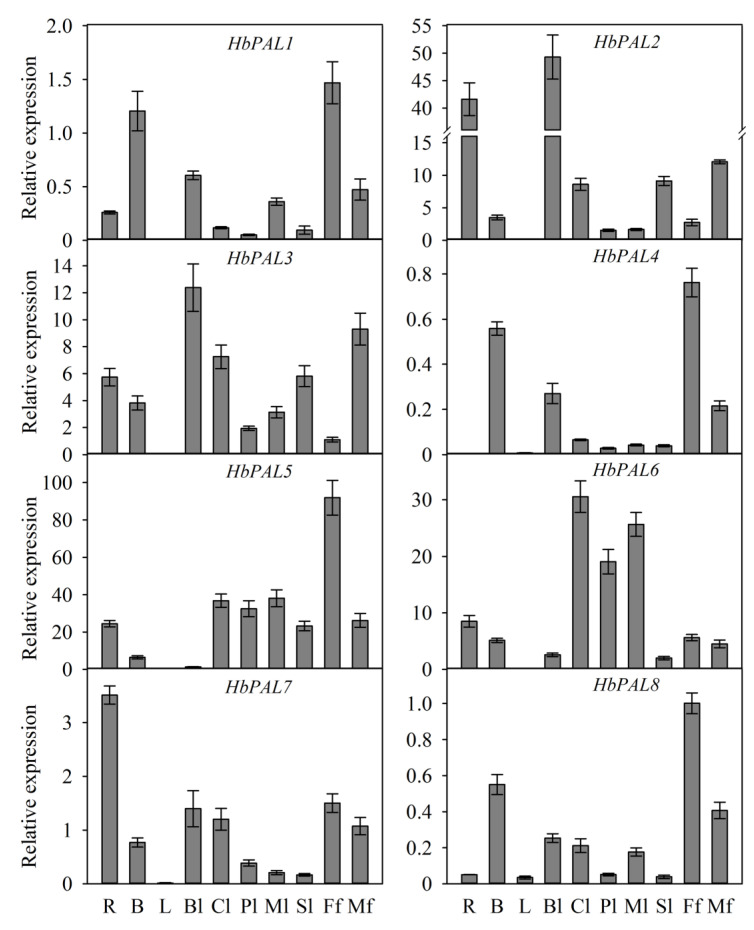
*HbPA* expression patterns within different rubber tree tissues. R, root; B, bark; L, latex; Ml, Sl, senescent leaf; mature leaf; Pl, pale-green leaf; Bl, bronze leaf; Cl, color-change leaf; Ff, female flower; Mf, male flower.

**Figure 6:**
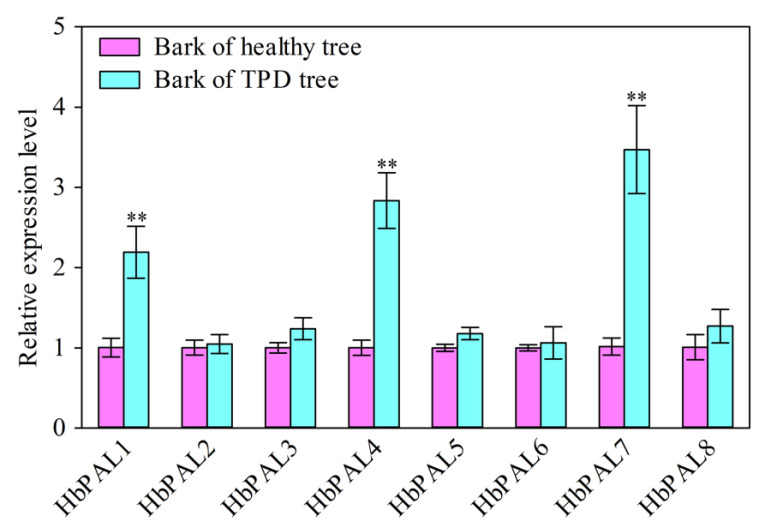
Analysis of changes in *HbPAL* expression in the bark of TPD and healthy rubber tree. Double asterisks (**) show significance when compared to the bark of the healthy tree (*p* < 0.01, *t*-test).

Female and male flowers, latex, barks, roots, and diverse developmental stages of leaves were among the rubber tree tissues used for quantitative real-time PCR (qPCR) detection of *HbPAL* expression levels. As seen in Figure 5, the eight *HbPALs* had diverse patterns of tissue expression. *HbPAL2*, *HbPAL7*, and *HbPAL8* were detected in all tested tissues, although their expression in latex was quite low. Additionally, *HbPAL1*, *HbPAL3*, *HbPAL5*, and *HbPAL6* had no expression in latex, whereas *HbPAL4* showed no expression in roots. However, high levels of expression of certain *HbPALs* were seen in specific tissues. For instance, *HbPAL2* was predominately expressed in roots and bronze leaves, while *HbPAL6* displayed relatively high expression in color-change and mature leaves. *HbPAL1*, *HbPAL4*, *HbPAL5*, and *HbPAL8* showed the greatest expression in female flowers, whereas the greatest expression of *HbPAL7* was detected in roots. The differential tissue expression patterns of *HbPALs* indicate that the *HbPALs* may play different functions in the development of different tissues. Additionally, the expression pattern of *HbPALs* was also analyzed in the healthy and tapping panel dryness (TPD) barks of rubber trees. In comparison to the healthy bark, significantly higher expression of *HbPAL1*, *HbPAL4*, and *HbPAL7* was detected in the TPD bark. However, no remarkable changes were seen within the remaining *HbPAL* gene expression in the TPD bark (Figure 6). The results suggest that *HbPAL1*, *HbPAL4*, and *HbPAL7* may participate in the TPD onset of the rubber tree.

### 2.6. Expression Analysis of HbPALs under Hormone Treatments

**Figure 7:**
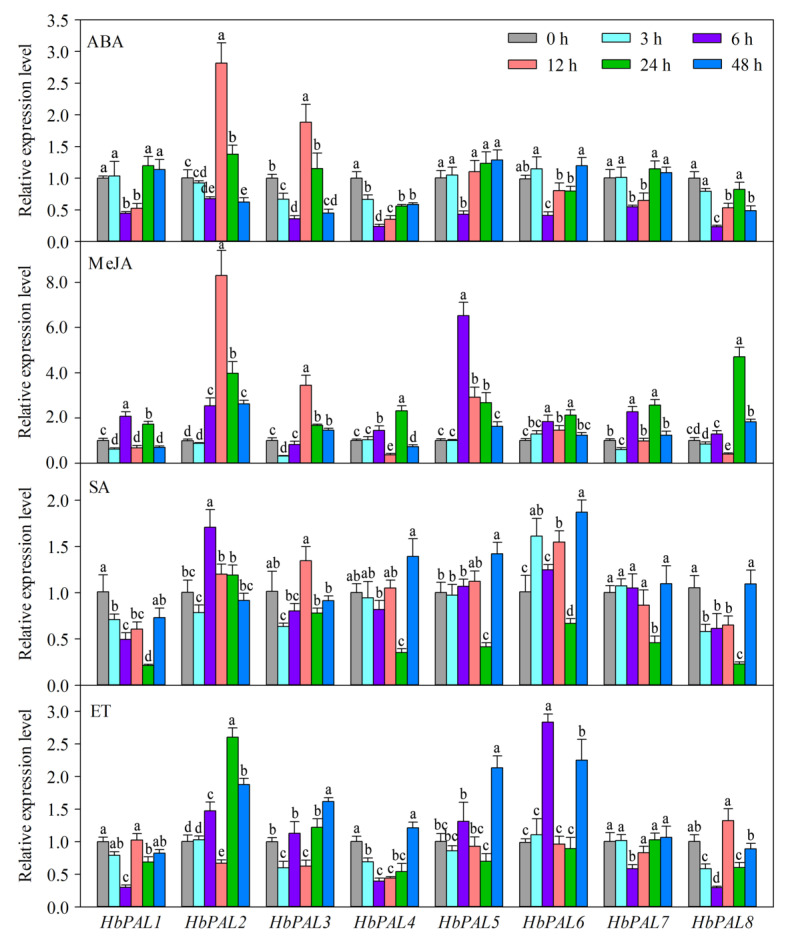
*HbPAL* genes expression analysis after different hormone applications. Significant differences are demonstrated by different letters (*p* < 0.05, Duncan’s multiple range test). SA, salicylic acid; ET, ethephon; MeJA, methyl jasmonate; ABA, abscisic acid.

Under abscisic acid (ABA) treatment, all the *HbPALs* showed obvious down-regulation at certain time points (Figure 7). *HbPAL4*, in particular, showed a notable down-regulation during each of the treated time points. At 6, 12, and 48 h, *HbPAL8* showed considerable repression, while *HbPAL3* showed significant repression at 3, 6, and 48 h. *HbPAL1* and *HbPAL7* were significantly down-regulated throughout 6–12 h, whereas *HbPAL2* displayed down-regulation at 6 and 48 h. At 6 h, there was a considerable down-regulation of *HbPAL5* and *HbPAL6*. However, at 12 h, *HbPAL2* and *HbPAL3* were significantly up-regulated. *HbPAL2*, *HbPAL5*, and *HbPAL6* were all markedly up-regulated with MeJA administration for 6 to 24 h, peaking in expression at 12, 6, and 24 h, respectively. Both down- and up-regulation were seen in *HbPAL1*, *HbPAL3*, *HbPAL4*, *HbPAL7*, and *HbPAL8* after MeJA treatment. For example, *HbPAL8* was significantly suppressed at 12 h but showed significant up-regulation between 24 and 48 h. Under SA treatment, *HbPAL2* exhibited up-regulation at 6 h. However, *HbPAL1*, *HbPAL3*, *HbPAL4*, *HbPAL7*, and *HbPAL8* were significantly repressed, and most of them showed the lowest expression at 24 h. At 24 h, *HbPAL5* and *HbPAL6* likewise exhibited notable down-regulation; however, their expression sharply increased thereafter. Under ethephon (ET) treatment, *HbPAL5* and *HbPAL6* were significantly up-regulated, whereas *HbPAL1*, *HbPAL4*, *HbPAL7*, and *HbPAL8* were significantly suppressed, with the lowest level at 6 h. At 12 h, *HbPAL2* and *HbPAL3* showed a considerable down-regulation, but at 48 h, they showed a significant up-regulation. In summary, these findings indicated that ABA, MeJA, SA, and ET regulated all *HbPALs*.

### 2.7. HbPAL Expression Analysis in Response to Abiotic Stresses

**Figure 8:**
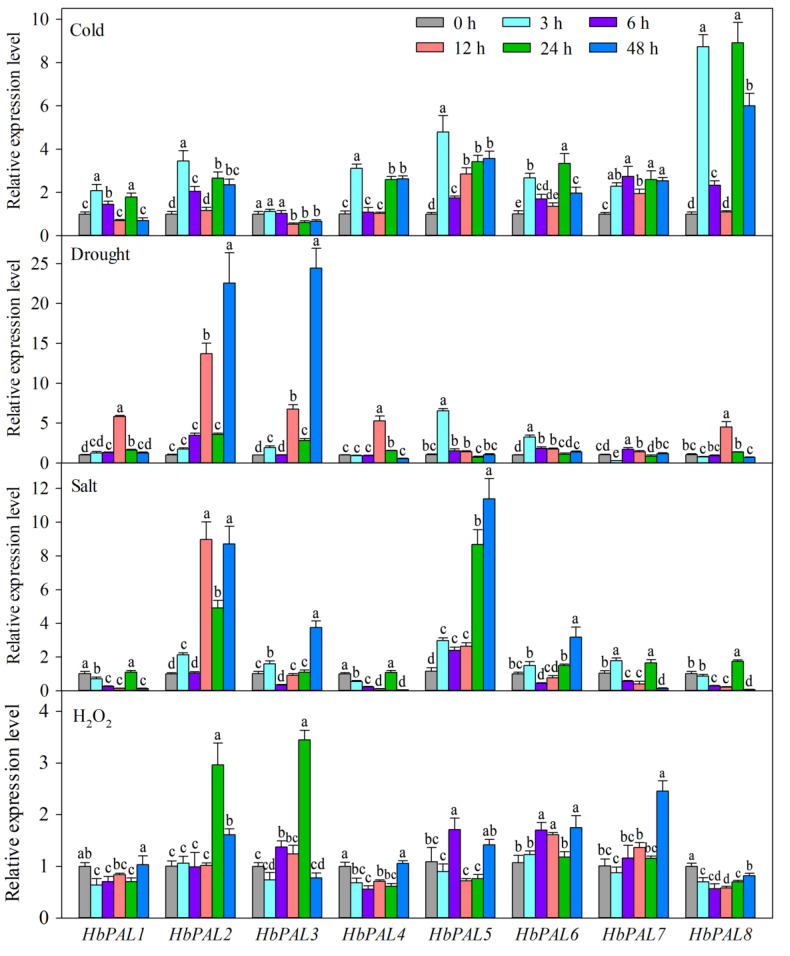
*HbPAL* gene expression profiles under different abiotic stress. Significant differences (*p* < 0.05, Duncan’s multiple range test) are demonstrated by different letters.

Under cold treatment, only *HbPAL3* was significantly down-regulated during 12–48 h, whereas all others exhibited significant up-regulation (Figure 8). Under PEG-induced drought stress, all *HbPALs* demonstrated considerable up-regulation at least at one treated time point; only *HbPAL7* exhibited significant down-regulation at 3 h. Under salt stress, *HbPAL2* and *HbPAL5* displayed remarkable up-regulation at nearly all managed time points; *HbPAL1* and *HbPAL4* expression were repressed during 3–12 h and 48 h treatments, with the lowest level at 48 h; *HbPAL7* and *HbPAL8* expression were repressed during 6–12 h and 48 h treatments, but their expression was remarkably enhanced at 24 h; *HbPAL3* and *HbPAL6* had similar expression patterns, demonstrating significant up-regulation at 3 h and 48 h whereas down-regulation at 6 h. Five *HbPALs* (*HbPAL2*, *HbPAL3*, *HbPAL5*, *HbPAL6*, and *HbPAL7*) were highly up-regulated, while the other three were dramatically down-regulated under H_2_O_2_-induced oxidative stress. All of these findings suggested that *HbPALs* responded to oxidant, salt, cold, and drought stressors.

### 2.8. HbPAL Expression Analysis Subjected to Biotic Stresses

**Figure 9:**
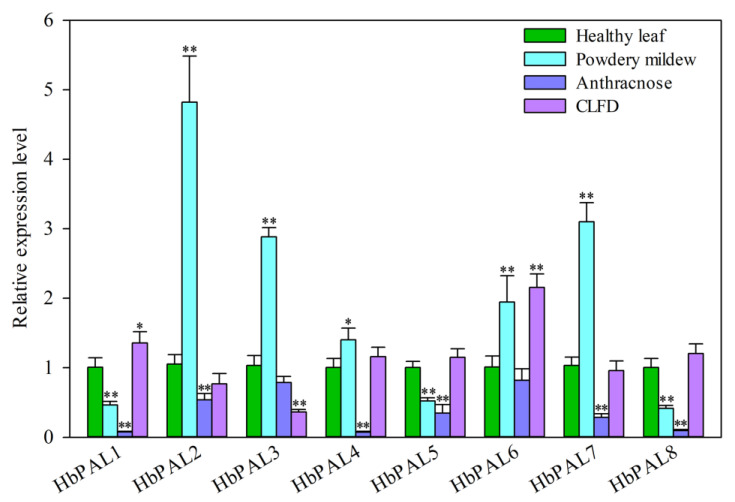
*HbPAL* genes expression analysis in leaves infected with powdery mildew, anthracnose, and *Corynespora* leaf fall disease (CFLD). Asterisks show a significant difference relative to the healthy leaves (*, *p* < 0.05; **, *p* < 0.01, *t*-test).

To determine whether *HbPALs* were responsive to biotic stress, their expression was analyzed against the three major fungal diseases, including powdery mildew, anthracnose, and *Corynespora* leaf fall disease (CFLD). As shown in Figure 9, *HbPAL2*, *HbPAL3*, *HbPAL4*, *HbPAL6*, and *HbPAL7* were dramatically up-regulated, whereas the other displayed significant down-regulation in powdery mildew-infested leaves relative to healthy leaves. The remaining six *HbPALs* were all significantly down-regulated in anthracnose-infested leaves relative to healthy leaves, with the exception of *HbPAL3* and *HbPAL6*. Only three *HbPALs* were found to be differentially expressed upon CFLD infection; *HbPAL1* and *HbPAL6* showed significant up-regulation, whereas *HbPAL3* showed significant down-regulation in CFLD-infested leaves as compared to healthy leaves.

## 3. Discussion

PALs contribute momentous functions in plant adaptation to environmental stresses as well as growth and development by participating in synthesizing important secondary metabolites [11,25]. Analysis of the PAL gene family at the whole-genome level has been carried out in certain plant species, such as *B. napus* [11], *O. sativa* [12], *P. trichocarpa* [13], *C. sativus* [1], and *J. regia* [14]. But the members of the rubber tree PAL family are still mostly unknown. In this study, we discovered the presence of a total of eight *HbPALs* in the genome of the rubber tree. All of these HbPAL proteins had the same basic structure as other PAL proteins, which included an N-terminal domain, an insert shielding region, a core domain, and an MIO domain [1,22,23]. Notably, the MIO domain of all HbPALs possessed the ASG tripeptide motif, which is the most typical conserved enzymatic active site of plant PALs [16,22,23]. These results indicated that the newly found HbPAL proteins were PALs and may have a similar catalytic function to the existing PALs. The PALs were found in the cytoplasm, according to a number of earlier investigations [13,14,16,25]. Our prediction results also demonstrated that every HbPAL was found in the cytoplasm, which is in line with prior investigations (Table 1).

The number of genes coding for PALs is species-specific and is determined by a multigene family [15]. In this study, we found that the rubber tree genome has eight PAL gene family members, which is higher than that in *P. trichocarpa* (5) and *C. sativus* (7) [1,13] but is lower than that in *O. sativa* (9), *J. regia* (12), potato (14), maize (10), and wheat (54) [12,14,15,16,26]. These eight *HbPAL* genes were localized to nine chromosomes of the rubber tree, including chromosomes 3, 7, 8, 10, 12, 13, 14, 16, and 18 (Figure 1). It was worth noting that both *HbPAL7* and *HbPAL8* had two copies. The two *HbPAL8* copies were found on chromosomes 3 and 18, respectively, while both *HbPAL7* copies were positioned in tandem on chromosome 16. These findings imply that the rubber tree’s PAL gene family expansion involved gene duplication events. The majority of the expansion and evolution within plant gene families is attributed to gene duplication, which encompasses whole-genome duplication, tandem duplication, and segmental duplication [27]. Several studies showed that the expansion of the PAL family genes was caused by gene duplications, as observed in *Cucumis sativus* [1], *Juglans regia* [14], *Solanum tuberosum* [15], willow [25], and cucumber and melon [22]. Therefore, we speculate that the variations in the number of *PALs* across diverse plant species may be caused by gene duplications, and it is probable that these duplication events play a crucial role in PAL gene family expansion.

Several studies [14,24,28] established a taxonomy of plant *PAL* genes and classified them into three distinct groups. In this study, we established the evolutionary connections among PAL proteins from *J. regia*, *P. trichocarpa*, *S. viminalis*, *A. thaliana*, *C. sativus*, *H. brasiliensis*, and *O. sativa*, and also found that these PALs were classed into three categories (Figure 2). Further analysis proved that the identified eight *HbPALs* were split into groups I and II (Figure 3A). This is consistent with the classification of *PAL* genes from other dicots, such as sorghum [28], willow [25], *Cephalotaxus hainanensis* [29], common walnut [14], and cucumber and melon [22]. These results suggest similar evolutionary trajectories among dicots. Previous studies indicated that the *PALs* within the same category exhibited similar exon/intron architectures [14,15,24]. Our findings supported this by demonstrating that the group I HbPALs had nearly identical exon lengths and identical exon/intron architectures (two exons and one intron) (Figure 3B). Furthermore, the HbPALs of group I had similar motif compositions (Figure 3C). In total, 16 motifs were shared by all group I HbPALs. Only three motifs were positioned in particular HbPALs of group I. For example, motif 19 existed only in HbPAL3 and HbPAL4. These particular motifs might be required for the particular roles of certain HbPALs. However, unlike group I *HbPALs*, quite different exon/intron structures and motif compositions were presented in the two *HbPALs* of group II. *HbPAL7* had one exon and 12 motifs, while *HbPAL8* possessed five exons and eight motifs. These differences may lead to functional differences between *HbPAL7* and *HbPAL8*.

Previous studies indicated that PAL family genes from several plant species displayed tissue-specific expression patterns [15,25,29,30]. Our study revealed that *HbPALs* had different expressions in various rubber tree tissues (Figure 5). Compared to other tissues, the female flowers exhibited higher levels of expression for *HbPAL1*, *HbPAL4*, *HbPAL5,* and *HbPAL8*. Several *CsPALs* also displayed the greatest expression in the cucumber female flowers [1], which is consistent with our findings. In addition, the female flowers of cucumber had the highest PAL activity [1]. These results suggested specific roles of PALs in female flower development. Analysis of the expression of *PALs* in plants like cucumber [1], *C. hainanensis* [29], and willow [25] revealed that some *PALs* were substantially expressed in root tissue that is rich in lignin. In this study, the greatest expression of *HbPAL7* was detected in the root, implying that this gene may participate in the biosynthesis of lignin in the root. In addition to lignin synthesis, *PALs* also participate in the synthesis of flavonoids, isoflavones, and anthocyanins [29]. The bronze leaf is rich in anthocyanins [31]. It is rather remarkable that *HbPAL2* and *HbPAL3* displayed the greatest expression in the bronze leaf, indicating the involvement of these two genes in the anthocyanin production of the bronze leaf. However, to date, there have been no reports on the functions of *HbPALs* in rubber tree tissue development. For the above-mentioned *HbPALs*, their functions in the development of specific tissues like female flowers, roots, and bronze leaves should be characterized through overexpression and gene knockout in the future.

Essential functions of promoter *cis*-acting elements include controlling transcription and gene expression. Previous investigations indicated that the promoters of *PALs* in wheat and potato contained numerous hormone and stress response-linked *cis*-acting elements [15,16]. By analyzing the *HbPALs* promoters, many important hormone-related elements were identified, including ABRE, GARE-motif, TGACG-motif, TCA-element, and ERE (Figure 4). In every *HbPAL* promoter, at least one hormone-responsive element was found. Especially, some elements only existed in the promoters of particular *HbPALs*, suggesting that the *cis*-elements vary in different family members. For instance, the TCA element, which is SA-responsive, was only found in the promoter of *HbPAL1*. At the same time, the ERE was detected in the promoters of all *HbPALs*. Similar to our results, nearly all promoters of potato *StPALs* contained the ERE [15]. However, the ERE was only identified in the promoter of *PbPAL1* in pear [24]. These results suggest that *cis*-elements in *PAL* promoters vary from species to species. In addition, in *HbPALs* promoters, we found various biotic and abiotic stress-linked *cis*-acting elements. These elements include LTR, which is a cold-responsive element, TC-rich repeats, MBS, which is a drought-inducibility element, which is a defense and stress-responsive element, and WUN-motif, which is a wound-responsive element. These *cis*-acting elements also appeared in the *PALs* promoters in cucumber, potato, wheat, and pear [1,15,16,24]. These findings indicate that some *PALs* may take part in response to abiotic or biotic stress. Several recent studies have demonstrated this [8,9]. For example, overexpressing of *FuPAL1* gene from *F. unibracteata* enhanced drought tolerance in *Arabidopsis* [8].

To investigate whether the expressions of *HbPALs* were affected by exogenous hormones and abiotic stimulus, *HbPAL* expression patterns were examined after ABA, ET, SA, MeJA, drought, cold, salt, and H_2_O_2_-oxidative stress treatments (Figure 7 and Figure 8). Our results indicated that all of these treatments prominently altered the expression of *HbPALs* at least at one time point, although their expression patterns were different. Several investigations have demonstrated that abiotic stressors and exogenous hormones had an impact on the *PAL* genes’ expressions [1,24,29]. *HbPAL2* was shown to be up-regulated by SA in a prior work [21]. Consistent with this, we also found that SA treatment significantly up-regulated *HbPAL2*’s expression. This also confirmed the reliability of our expression analysis. In cucumber, all *CsPALs* exhibited up-regulation under low temperatures [1]. In this study, cold stress also up-regulated the other *HbPAL* genes, excluding *HbPAL3*. Olsen et al. [32] showed that PAL activity and expression increased under cold stress; meanwhile, phenylpropanoid metabolites accumulated. The accumulation of phenylpropanoid metabolites can protect plants against cold stress [32,33,34]. Therefore, we conjecture that up-regulation of *HbPAL* genes is a strategy for rubber trees to adapt to low temperatures. *HbPAL* promoters contained abiotic stress-related *cis*-elements, and their expressions were regulated by abiotic stresses, implying that *HbPLAs* have significant roles in adapting to abiotic stress. In the future, it is necessary to validate the roles of *HbPALs* in rubber tree abiotic stress tolerance through genetic engineering methods.

Derivatives of phenolpropanoid have broad-spectrum antibacterial effects and are crucial for plant defense against pathogen infections [35,36]. PAL, as the critical enzyme for the synthesis of phenylpropanoid compounds, has been demonstrated to play a positive function in plants resistant to pathogens [35]. For example, overexpression of *Stylosanthes humilis ShPAL* gene enhanced resistance to *Cercospora nicotianae* in tobacco [37]. In rice, reduced resistance to three different diseases (rice blast, sheath blight, and bacterial blight) was observed in the heterozygous mutant of *ospal4* [6]. In addition, soybean plants silenced for five *PAL* isoforms were more susceptible to *Phytophthora sojae* [38]. Given the prominent functions of *PALs* in plant resistance to disease, the changes in *HbPAL* expression in powdery mildew, anthracnose, and CFLD-infected leaves were detected (Figure 9). In comparison to the control group, three *HbPALs* displayed down-regulation, and the others were up-regulated in powdery mildew-infected leaves. The expression analysis of 54 *TaPALs* in wheat also found that most *TaPALs* showed up-regulation, and just a small portion exhibited down-regulation after powdery mildew infection [16]. Additionally, Powdery mildew-infected leaves had significantly higher *PAL* gene expression and activity than the healthy leaves in pumpkin [39]. These findings suggest that increasing *PAL* expression seems to be a common strategy for plants to cope with powdery mildew. Differently from their response to powdery mildew, except for *HbPAL3* and *HbPAL6* with no change in expression, all other *HbPALs* were down-regulated in anthracnose-infected leaves. Bergamini Lopes et al. [40] showed that the activity of PAL was enhanced after treatments that strengthened anthracnose resistance in rubber trees. Therefore, we speculate that the susceptibility to anthracnose may be related to the decrease in *HbPAL* expression in the rubber tree. However, the roles of *HbPALs* in resistance to anthracnose, powdery mildew, and CFLD in rubber trees are still unclear. More studies are needed on the functions of *HbPALs* in disease resistance.

TPD, with the main symptom of locally or completely no latex flowing out from the cutting line after tapping, is the main cause of latex yield and economic losses in rubber trees. The bark structure of the TPD tree changed obviously as compared with the healthy bark. As the increase in TPD extent, the presence of stone cells, also as sclereids, increased significantly in the bark, and the position of their formation shifted obviously inward [41]. Parenchyma cells may harden their cell walls by the deposition of lignin and cellulose, which leads to the production of stone cells [42,43]. Research has shown that the formation of stone cells is strongly associated with lignin and cellulose production, transport, and deposition [43]. PAL, as an essential enzyme for lignin synthesis, may participate in the stone cell formation in the bark of TPD rubber tree, so we analyzed the differences in *HbPAL* expression between healthy and TPD barks (Figure 6). Our results indicated that *HbPAL1*, *HbPAL4*, and *HbPAL7* expression were significantly enhanced in TPD bark relative to healthy bark. The up-regulation of *HbPAL1*, *HbPAL4*, and *HbPAL7* may be responsible for promoting stone cell formation in TPD tree bark by enhancing lignin biosynthesis. Further experiments can be conducted to see if it is possible to inhibit the production of stone cells by reducing the expression of these three *HbPALs* in bark tissue, thereby preventing the onset of TPD in rubber trees.

## 4. Materials and Methods

### 4.1. Plant Materials and Treatments

To investigate the tissue expression characteristics of rubber tree PAL family genes, various tissue specimens, including male flower (Mf), female flower (Ff), senescent leaf (Sl), mature leaf (Ml), pale-green leaf (Pl), color-change leaf (Cl), bronze leaf (Bl), latex (L), and bark (B), were harvested from rubber trees (16-year-old) in May 2022. These rubber trees were planted at the CATAS experimental farm (109°49′ E, 19°47′ N) in Danzhou, Hainan, China, with the variety of Reyan 7-33-97. The root (R) samples were harvested from tissue culture plants of Reyan 7-33-97 (two-year-old). To study the changes in *PAL* expression in the bark of TPD trees, bark specimens were taken from TPD or healthy rubber trees in accordance with the procedures outlined in our previous study [44]. To investigate the effects of pathogen infection on *HbPAL* expression, the mature leaves showing typical symptoms of powdery mildew, anthracnose, or *Corynespora* leaf fall disease (CFLD) were collected from the corresponding diseased plants of Reyan 7-33-97, which were planted in the field for three years. The control leaves were collected from healthy plants without disease infection. One biological replication was made up of mixed samples collected from at least six different trees, and each specimen was prepared with three separate biological replicates. All specimens were kept at −80 °C after being frozen in liquid nitrogen.

To investigate the effects of hormone and abiotic stress treatments on *HbPAL* expression, uniform Reyan 7-33-97 tissue culture plants (approximately two years old, six leaves) were chosen for hormone and abiotic stress treatments. These seedlings were grown in a greenhouse under natural lighting and temperature in plastic bags filled with a blend of vermiculite, soil, and peat (v/v/v = 1:1:1). These selected seedlings were moved into an artificial climate box with the following settings: 16 h light/8 h dark, 27 °C, and 75% relative humidity. Following adaptation for 3 days, these plants were grouped and subjected to various treatments. For hormone and H_2_O_2_ treatments, 200 μmol/L ABA (Sangon Biotech, Shanghai, China), 10 mmol/L ET (Solarbio, Beijing, China), 200 μmol/L MeJA (Sigma, Saint-Louis, MO, USA), 5 mmol/L SA (Solarbio, Beijing, China), or 20 mmol/L H_2_O_2_ (Xilong Scientific, Shantou, China) were applied according to the previous studies [45,46]. Treatments for cold, drought, and salt stress were carried out largely according to Cao et al.’s [46] description. In short, the plants were treated to cold stress by being kept in a 4 °C artificial climate box under 16 h light/8 h dark conditions. For salt and PEG (polyethylene glycol)-induced drought stresses, the plant root matrix was washed out with water and then immersed the roots in 400 mmol/L NaCl or 20% PEG6000 (Sinopharm, Bejing, China) solution, respectively. These two treatments were also carried out in artificial climate boxes at 27 °C, 16 h light/8 h dark, and 175% relative humidity. For every treatment, three repetitions with three plants each were carried out. As previously mentioned [46], the leaves were taken at 0, 3, 6, 12, 24, and 48 h following treatments. All specimens were kept at −80 °C after being frozen in liquid nitrogen.

### 4.2. Identification and Sequence Analysis of Rubber Tree PAL Gene Family

Four *Arabidopsis* PALs’ amino acid sequences were downloaded from NCBI. Using *Arabidopsis* PAL protein sequences as references, a BLASTp search was conducted against the rubber tree genome of Reyan 7-33-97 in HeveaDB (http://hevea.catas.cn/home/index) (accessed on 10 April 2022) [47]. Hits with an E-value less than 10^−5^ were obtained. 

After removing redundant sequences, the InterProScan (http://www.ebi.ac.uk/interpro/scan.html) (accessed on 6 May 2022), Conserved Domain Database (CDD) (https://www.ncbi.nlm.nih.gov/Structure/cdd/wrpsb.cgi) (accessed on 6 May 2022), and PROSITE (https://prosite.expasy.org/index.html) (accessed on 6 May 2022) were used to analyze all of the HbPAL candidate protein sequences to validate the presence of the PAL domain. The isoelectric point (pI) and theoretical molecular weight (Mw) of HbPAL proteins were predicted by the Compute pI/Mw tool of Expasy (https://www.expasy.org/) (accessed on 10 May 2022). Every HbPAL protein’s subcellular location was predicted using CELLO [48].

### 4.3. Phylogenetic and Sequence Alignment Analysis

Multiple sequence alignment was implemented by Clustal Omega (https://www.ebi.ac.uk/Tools/msa/clustalo/) (accessed on 20 May 2022). To explore the evolutionary relationship of PALs in plants, the phylogenetic tree of PAL family proteins from *J. regia*, *P. trichocarpa*, *A. thaliana*, *S. viminalis*, *C. sativus*, *H. brasiliensis*, and *O. sativa* was constructed by MEGA X [49] with Neighbor-Joining method (1000 bootstrap replicates). Using Evolview v3 [50], the resulting phylogenetic tree was further beautified.

### 4.4. Analysis of Gene Structure and Chromosome Location of HbPALs

The *HbPALs* genomic DNA and coding sequences were obtained from the HeveaDB database. The *HbPAL* exon-intron architectures were created by GSDS2.0 [51] based on aligning genomic DNA and coding sequences. The chromosomal localization of *HbPAL* genes was ascertained by matching *HbPAL* genomic DNA sequences to the chromosome-based rubber tree genome of GT1 [52] and was drawn by Mapchart [53].

### 4.5. Cis-Acting Elements and Conserved Motifs Analysis

Reyan 7-33-97 genome sequences were adopted to extract the upstream 2 kb region of each *HbPAL* initiation codon. The promoter *cis*-acting elements were obtained by PlantCARE [54] and visualized using GSDS2.0 [51]. The MEME [55] was applied to predict conserved motifs of HbPAL proteins, with parameter settings referring to Zhan et al. [16].

### 4.6. RNA Isolation and qPCR Detection

To detect the expression of *HbPALs* in each specimen, qPCR analysis was performed. Using the RNAprep Pure Plant Plus Kit (Tiangen, Beijing, China), total RNA was isolated from the obtained specimens. The reagent kits, reaction systems, and procedures for cDNA synthesis and qPCR detection were the same as in our previous report [44]. The internal control was *HbUBC4* gene [56]. Primer3web (https://bioinfo.ut.ee/primer3/ (accessed on 8 June 2022), version 4.1.0) was utilized to design the qPCR primer pairs for each gene (Table S3). Agarose gel electrophoresis, as well as melting curve analyses, were carried out to evaluate the primer pair’s specificity. A mixed cDNA sample of different tissues was diluted three times to create a standard curve for calculating the primer pair’s PCR amplification efficiency. According to the 2^−ΔΔCT^ method [30], relative gene expression was computed and normalized to the quantity of *HbUBC4*. Data were presented as mean ± SD, which was calculated from three biological replicate values. Significant differences between TPD bark and healthy bark, diseased leaves, and healthy leaves were conducted using a *t*-test in SigmaPlot 12.0 (Systat Software Inc., San Jose, CA, USA). To determine whether there are significant differences in gene expression among different treatment time points under hormone or abiotic stress treatment, the data were analyzed using one-way ANOVA in SigmaPlot 12.0 (Systat Software Inc., San Jose, CA, USA). Comparisons between multiple treatment time points were performed with Duncan’s multiple range test, and *p* < 0.05 was considered to be statistically significant.

## 5. Conclusions

In this study, we conducted a comprehensive identification and characterization of the PAL family in *H. brasiliensis*. We identified eight *PAL* genes (*HbPAL1*–*HbPAL8*) in the rubber tree, which were distributed on nine chromosomes. These genes were separated into groups I and II, and the group I *HbPALs* (*HbPAL1*–*HbPAL6*) shared similar exon-intron architectures and motif compositions. The expression analysis results indicated that the tissue expression patterns of *HbPALs* varied. Promoter *cis*-acting element and qPCR detection showed that abiotic stimuli (oxidative stress, salt, drought, and cold) and many hormones (ABA, ET, SA, and MeJA) modulated the expression of *HbPALs*. Additionally, most *HbPAL* expressions were repressed in anthracnose-infested leaves but enhanced in powdery mildew-infested leaves. In CFLD-infested leaves, *HbPAL1* and *HbPAL6* were up-regulated, whereas *HbPAL3* was down-regulated. Moreover, *HbPAL1*, *HbPAL4*, and *HbPAL7* showed significant up-regulation in the TPD bark, suggesting a possible role for *HbPALs* in TPD onset. In summary, our research offers a thorough comprehension of the traits of *HbPALs*, laying the groundwork for further investigations on the biological functions of *PAL* family genes in rubber trees.

## Figures and Tables

**Table 1:** *HbPAL* genes characteristics in rubber tree.

Gene Name	Gene ID	ORF Length (bp)	Protein Length (aa)	GenBank Accession No.	pI	Mw (kDa)	Subcellular Localization
*HbPAL1*	scaffold0093_366908	2142	713	XP_021667240.1	6.37	77.50	Cytoplasm
*HbPAL2*	scaffold0050_2730293	2145	714	XP_021660472.1	6.06	77.84	Cytoplasm
*HbPAL3*	scaffold0177_1718462	2145	714	XP_021673747.1	6.06	77.54	Cytoplasm
*HbPAL4*	scaffold1042_98893	2145	714	XP_021650034.1	6.06	77.87	Cytoplasm
*HbPAL5*	scaffold0382_321936	2109	702	XP_021684500.1	6.04	76.74	Cytoplasm
*HbPAL6*	scaffold0063_2642034	2130	709	XP_021665269.1	6.55	77.34	Cytoplasm
*HbPAL7*	scaffold0388_448358	2136	711	XP_021684949.1	5.85	78.29	Cytoplasm
*HbPAL8*	scaffold0735_280091	1569	522	-	8.17	57.1	Cytoplasm

Mw: molecular weight; pI: isoelectric point; ORF: open reading frame; kDa: kilo Dalton; bp: base pair; aa: amino acid.

## Data Availability

All data were given in the article and Supplementary Materials. The rubber tree genome data and the *HbPALs* sequences can be downloaded from HeveaDB (http://hevea.catas.cn/home/index) (accessed on 10 April 2022).

## Supplementary Materials

The following supporting information can be downloaded at https://www.mdpi.com/article/10.3390/ijms25095052/s1.

## References

[1] Shang Q.M., Li L., Dong C.J. (2012). Multiple tandem duplication of the phenylalanine ammonia-lyase genes in *Cucumis sativus* L.. Planta.

[2] Barros J., Dixon R.A. (2020). Plant phenylalanine/tyrosine ammonia-lyases. Trends Plant Sci..

[3] Lu J., Shi Y., Li W., Chen S., Wang Y., He X., Yin X. (2019). *RcPAL*, a key gene in lignin biosynthesis in *Ricinus communis* L.. BMC Plant Biol..

[4] Persic M., Mikulic-Petkovsek M., Halbwirth H., Solar A., Veberic R., Slatnar A. (2018). Red walnut: Characterization of the phenolic profiles, activities and gene expression of selected enzymes related to the phenylpropanoid pathway in pellicle during walnut development. J. Agric. Food Chem..

[5] Sui Z., Luo J., Yao R., Huang C., Zhao Y., Kong L. (2019). Functional characterization and correlation analysis of phenylalanine ammonia-lyase (PAL) in coumarin biosynthesis from *Peucedanum praeruptorum* Dunn. Phytochemistry.

[6] Tonnessen B.W., Manosalva P., Lang J.M., Baraoidan M., Bordeos A., Mauleon R., Oard J., Hulbert S., Leung H., Leach J.E. (2015). Rice phenylalanine ammonia-lyase gene *OsPAL4* is associated with broad spectrum disease resistance. Plant Mol. Biol..

[7] Zhang H., Huang Q., Yi L., Song X., Li L., Deng G., Liang J., Chen F., Yu M., Long H. (2021). PAL-mediated SA biosynthesis pathway contributes to nematode resistance in wheat. Plant J..

[8] Qin Y., Li Q., An Q., Li D., Huang S., Zhao Y., Chen W., Zhou J., Liao H. (2022). A phenylalanine ammonia lyase from *Fritillaria unibracteata* promotes drought tolerance by regulating lignin biosynthesis and SA signaling pathway. Int. J. Biol. Macromol..

[9] Fan L., Shi G., Yang J., Liu G., Niu Z., Ye W., Wu S., Wang L., Guan Q. (2022). A protective role of phenylalanine ammonia-lyase from *Astragalus membranaceus* against saline-alkali stress. Int. J. Mol. Sci..

[10] Yao L., Zhang H., Liu Y., Ji Q., Xie J., Zhang R., Huang L., Mei K., Wang J., Gao W. (2022). Engineering of triterpene metabolism and overexpression of the lignin biosynthesis gene *PAL* promotes ginsenoside Rg_3_ accumulation in ginseng plant chassis. J. Integr. Plant Biol..

[11] Zhang H., Zhang X., Zhao H., Hu J., Wang Z., Yang G., Zhou X., Wan H. (2023). Genome-wide identification and expression analysis of phenylalanine ammonia-lyase (PAL) family in rapeseed (*Brassica napus* L.). BMC Plant Biol..

[12] Gho Y.S., Kim S.J., Jung K.H. (2020). Phenylalanine ammonia-lyase family is closely associated with response to phosphate deficiency in rice. Genes Genom..

[13] Shi R., Shuford C.M., Wang J.P., Sun Y.H., Yang Z., Chen H.C., Tunlaya-Anukit S., Li Q., Liu J., Muddiman D.C. (2013). Regulation of phenylalanine ammonia-lyase (PAL) gene family in wood forming tissue of *Populus trichocarpa*. Planta.

[14] Yan F., Li H., Zhao P. (2019). Genome-wide identification and transcriptional expression of the *PAL* gene family in common walnut (*Juglans regia* L.). Genes.

[15] Mo F., Li L., Zhang C., Yang C., Chen G., Niu Y., Si J., Liu T., Sun X., Wang S. (2022). Genome-wide analysis and expression profiling of the phenylalanine ammonia-lyase gene family in *Solanum tuberosum*. Int. J. Mol. Sci..

[16] Zhan C., Li Y., Li H., Wang M., Gong S., Ma D., Li Y. (2022). Phylogenomic analysis of phenylalanine ammonia-lyase (PAL) multigene family and their differential expression analysis in wheat (*Triticum aestivum* L.) suggested their roles during different stress responses. Front. Plant Sci..

[17] Hou X., Shao F., Ma Y., Lu S. (2013). The phenylalanine ammonia-lyase gene family in *Salvia miltiorrhiza*: Genome-wide characterization, molecular cloning and expression analysis. Mol. Biol. Rep..

[18] Chen Y., Li F., Tian L., Huang M., Deng R., Li X., Chen W., Wu P., Li M., Jiang H. (2017). The phenylalanine ammonia lyase gene *LjPAL1* is involved in plant defense responses to pathogens and plays diverse roles in *Lotus japonicus*-rhizobium symbioses. Mol. Plant Microbe Interact..

[19] Yoshikawa M., Luo W., Tanaka G., Konishi Y., Matsuura H., Takahashi K. (2018). Wounding stress induces phenylalanine ammonia lyases, leading to the accumulation of phenylpropanoids in the model liverwort *Marchantia polymorpha*. Phytochemistry.

[20] Tang C., Yang M., Fang Y., Luo Y., Gao S., Xiao X., An Z., Zhou B., Zhang B., Tan X. (2016). The rubber tree genome reveals new insights into rubber production and species adaptation. Nat. Plants.

[21] Deenamo N., Kuyyogsuy A., Khompatara K., Chanwun T., Ekchaweng K., Churngchow N. (2018). Salicylic acid induces resistance in rubber tree against *Phytophthora palmivora*. Int. J. Mol. Sci..

[22] Dong C.J., Cao N., Zhang Z.G., Shang Q.M. (2016). Phenylalanine ammonia-lyase gene families incucurbit species: Structure, evolution, and expression. J. Integr. Agric..

[23] Ritter H., Schulz G.E. (2004). Structural basis for the entrance into the phenylpropanoid metabolism catalyzed by phenylalanine ammonia-lyase. Plant Cell.

[24] Li G., Wang H., Cheng X., Su X., Zhao Y., Jiang T., Jin Q., Lin Y., Cai Y. (2019). Comparative genomic analysis of the *PAL* genes in five rosaceae species and functional identification of chinese white pear. Peer J..

[25] de Jong F., Hanley S.J., Beale M.H., Karp A. (2015). Characterisation of the willow phenylalanine ammonia-lyase (PAL) gene family reveals expression differences compared with poplar. Phytochemistry.

[26] Yuan W., Jiang T., Du K., Chen H., Cao Y., Xie J., Li M., Carr J.P., Wu B., Fan Z. (2019). Maize phenylalanine ammonia-lyases contribute to resistance to sugarcane mosaic virus infection, most likely through positive regulation of salicylic acid accumulation. Mol. Plant Pathol..

[27] Freeling M. (2009). Bias in plant gene content following different sorts of duplication: Tandem, whole-genome, segmental, or by transposition. Annu. Rev. Plant Biol..

[28] Pant S., Huang Y. (2022). Genome-wide studies of *PAL* genes in sorghum and their responses to aphid infestation. Sci. Rep..

[29] He Y., Zhong X., Jiang X., Cong H., Sun H., Qiao F. (2020). Characterisation, expression and functional analysis of *PAL* gene family in *Cephalotaxus hainanensis*. Plant Physiol. Biochem..

[30] Schmittgen T.D., Livak K.J. (2008). Analyzing real-time PCR data by the comparative c(t) method. Nat. Protoc..

[31] Huang T., Xin S., Fang Y., Chen T., Chang J., Ko N.C.K., Huang H., Hua Y. (2021). Use of a novel R2R3-MYB transcriptional activator of anthocyanin biosynthesis as visual selection marker for rubber tree (*Hevea brasiliensis*) transformation. Ind. Crop. Prod..

[32] Olsen K.M., Lea U.S., Slimestad R., Verheul M., Lillo C. (2008). Differential expression of four Arabidopsis *PAL* genes; *PAL1* and *PAL2* have functional specialization in abiotic environmental-triggered flavonoid synthesis. J. Plant Physiol..

[33] Siboza X.I., Bertling I., Odindo A.O. (2014). Salicylic acid and methyl jasmonate improve chilling tolerance in cold-stored lemon fruit (*Citrus limon*). J. Plant Physiol..

[34] Liu Y., Pan J., Ni S., Xing B., Cheng K., Peng X. (2022). Transcriptome and metabonomics combined analysis revealed the defense mechanism involved in hydrogen-rich water-regulated cold stress response of *Tetrastigma hemsleyanum*. Front. Plant Sci..

[35] Wang R., Wang G.L., Ning Y. (2019). PALs: Emerging key players in broad-spectrum disease resistance. Trends Plant Sci..

[36] Dixon R.A., Achnine L., Kota P., Liu C.J., Reddy M.S., Wang L. (2002). The phenylpropanoid pathway and plant defence—A genomics perspective. Mol. Plant Pathol..

[37] Way H.M., Kazan K., Mitter N., Goulter K.C., Birch R.G., Manners J.M. (2002). Constitutive expression of a phenylalanine ammonia-lyase gene from *Stylosanthes humilis* in transgenic tobacco leads to enhanced disease resistance but impaired plant growth. Physiol. Mol. Plant Pathol..

[38] Shine M.B., Yang J.W., El-Habbak M., Nagyabhyru P., Fu D.Q., Navarre D., Ghabrial S., Kachroo P., Kachroo A. (2016). Cooperative functioning between phenylalanine ammonia lyase and isochorismate synthase activities contributes to salicylic acid biosynthesis in soybean. New Phytol..

[39] Zhang S., Liu J., Xu B., Zhou J. (2021). Differential responses of *Cucurbita pepo* to *Podosphaera xanthii* reveal the mechanism of powdery mildew disease resistance in pumpkin. Front. Plant Sci..

[40] Bergamini Lopes M.P., Gomes M.E., Celin G.D.S., Bello H.N., Henrique R.L.P., Magalhaes I.P., Santos L.V., Tropaldi L., Pascholate S.F., Furtado E.L. (2022). Initial studies of the response of rubber tree seedlings treated with saprobic fungi from the semiarid region of northeast brazil to anthracnose. Plants.

[41] Lu Y., Zhang S., Wang Z., Tian W., Shi M. (2021). Bark structure of the clone RY7-33-97 with different level of tapping panel dryness (TPD) in rubber tree (*Hevea brasiliensis* muell. Arg.). Chin. J. Trop. Crops.

[42] Zhang L., Kamitakahara H., Takano T., Morimoto T., Sakamoto S., Mitsuda N., Itai A. (2023). Stone cell formation in the pedicel of pears and apples. Planta.

[43] Wang R., Xue Y., Fan J., Yao J.L., Qin M., Lin T., Lian Q., Zhang M., Li X., Li J. (2021). A systems genetics approach reveals PbrNSC as a regulator of lignin and cellulose biosynthesis in stone cells of pear fruit. Genome Biol..

[44] Liu H., Hu Y., Yuan K., Feng C., He Q., Sun L., Wang Z. (2022). Genome-wide identification of lncRNAs, miRNAs, mRNAs and their regulatory networks involved in tapping panel dryness in rubber tree (*Hevea brasiliensis*). Tree Physiol..

[45] Qin B., Zheng F., Zhang Y. (2015). Molecular cloning and characterization of a *Mlo* gene in rubber tree (*Hevea brasiliensis*). J. Plant Physiol..

[46] Cao Y., Xiang X., Geng M., You Q., Huang X. (2017). Effect of *HbDHN1* and *HbDHN2* genes on abiotic stress responses in *Arabidopsis*. Front. Plant Sci..

[47] Cheng H., Matsui M., Chow K.S. (2020). HeveaDB: A hub for rubber tree genetic and genomic resources. The Rubber Tree Genome.

[48] Yu C.S., Chen Y.C., Lu C.H., Hwang J.K. (2006). Prediction of protein subcellular localization. Proteins.

[49] Mello B. (2018). Estimating timetrees with MEGA and the timetree resource. Mol. Biol. Evol..

[50] Subramanian B., Gao S., Lercher M.J., Hu S., Chen W.H. (2019). Evolview v3: A webserver for visualization, annotation, and management of phylogenetic trees. Nucleic Acids Res..

[51] Hu B., Jin J., Guo A.Y., Zhang H., Luo J., Gao G. (2015). GSDS 2.0: An upgraded gene feature visualization server. Bioinformatics.

[52] Liu J., Shi C., Shi C.C., Li W., Zhang Q.J., Zhang Y., Li K., Lu H.F., Shi C., Zhu S.T. (2020). The chromosome-based rubber tree genome provides new insights into spurge genome evolution and rubber biosynthesis. Mol. Plant..

[53] Voorrips R.E. (2002). MapChart: Software for the graphical presentation of linkage maps and QTLs. J. Hered..

[54] Lescot M., Dehais P., Thijs G., Marchal K., Moreau Y., Van de Peer Y., Rouzé P., Rombauts S. (2002). PlantCARE, a database of plant *cis*-acting regulatory elements and a portal to tools for in silico analysis of promoter sequences. Nucleic Acids Res..

[55] Bailey T.L., Williams N., Misleh C., Li W.W. (2006). MEME: Discovering and analyzing DNA and protein sequence motifs. Nucleic Acids Res..

[56] Li H., Qin Y., Xiao X., Tang C. (2011). Screening of valid reference genes for real-time RT-PCR data normalization in *Hevea brasiliensis* and expression validation of a sucrose transporter gene *HbSUT3*. Plant Sci..

