# Supplementary material for: Genome-Wide Identification and Expression Profile Analysis of the Phenylalanine Ammonia-Lyase Gene Family in Hevea brasiliensis
Source: Int J Mol Sci. 2024 May 6;25(9):5052. doi: 10.3390/ijms25095052 (PMC11084274; doi:10.3390/ijms25095052)
Supplement: Supplementary file 1 [file ijms-25-05052-s001.zip › ijms-2937641-supplementary.pdf]

Genome-Wide Identification and Expression Profile Analysis of the Phenylalanine Ammonia-Lyase Gene Family in *Hevea brasiliensis*

Hui Liu*,†, Qiguang He†, Yiyu Hu, Ruilin Lu, Shuang Wu, Chengtian Feng, Kun Yuan and Zhenhui Wang*

Key Laboratory of Biology and Genetic Resources of Rubber Tree, Ministry of Agriculture and Rural Affairs/State Key Laboratory Incubation Base for Cultivation & Physiology of Tropical Crops, Rubber Research Institute, Chinese Academy of Tropical Agricultural Sciences, Haikou 571101, China

† These authors contributed equally to this work.

* Correspondence: liuhui@catas.cn (H. L.); wzh36@catas.cn (Z.W.)

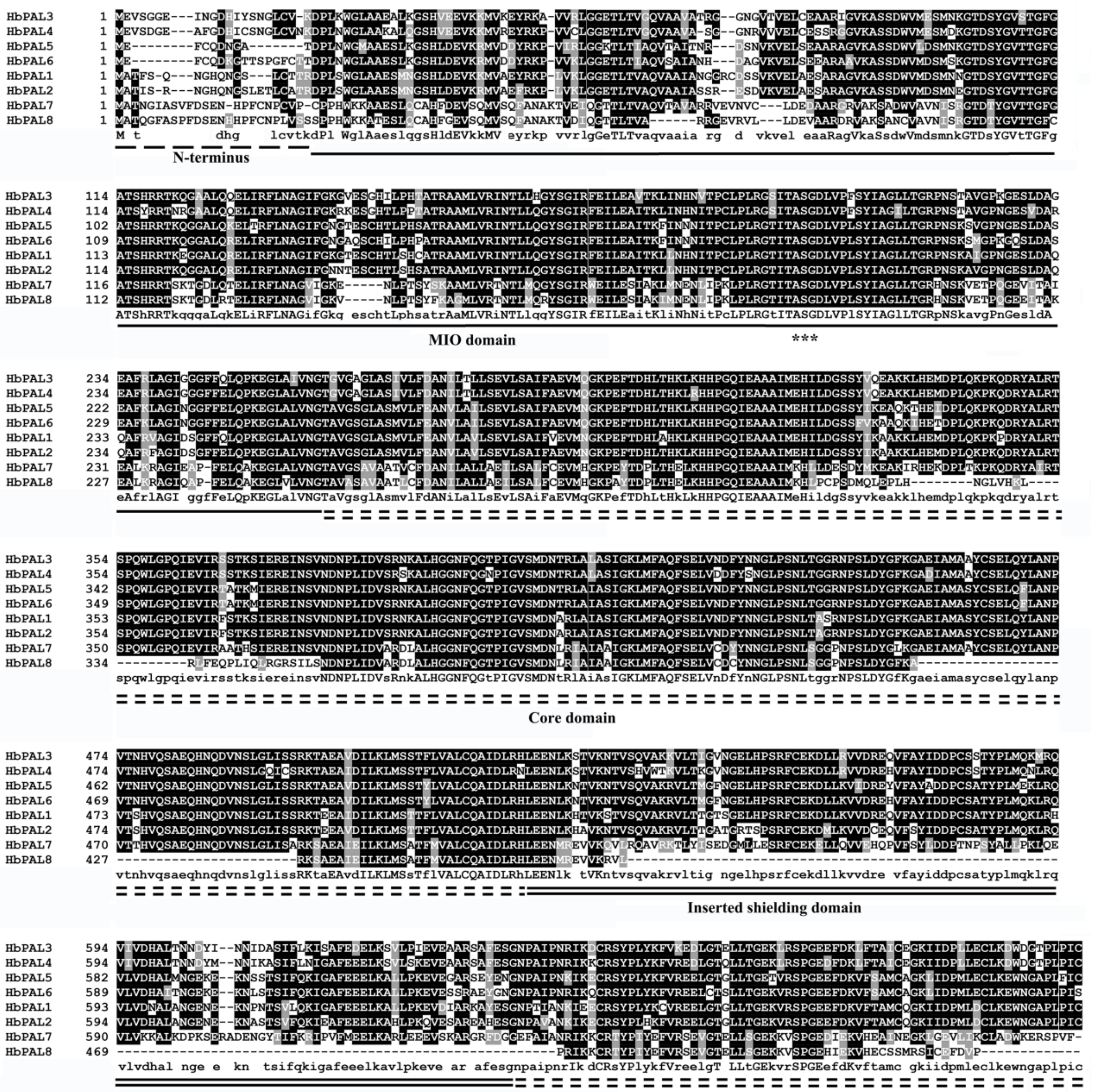

Figure S1. Multiple sequence alignment of HbPAL proteins. The four functional domains, including the N-terminus (single dashed line), MIO domain (single solid line), core domain (double dashed line), and inserted shielding domain (double solid line), are indicated. The conserved Ala-Ser-Gly (ASG) tripeptide motif is marked with asterisks (***).

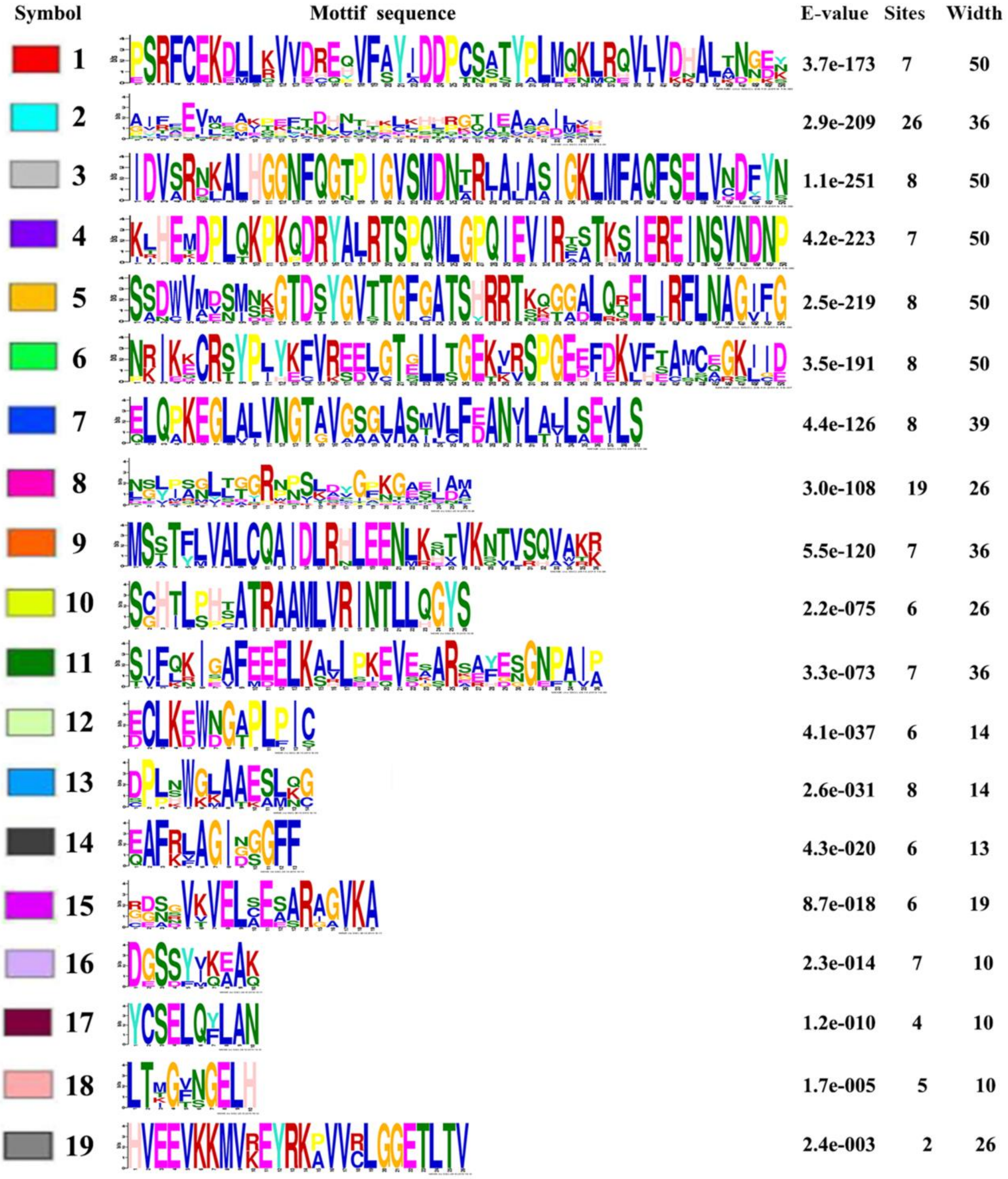

Figure S2. Sequence logos of conserved motifs identified in HbPAL proteins by MEME analysis.

Table S1. Percent identity matrix of HbPAL proteins.

	HbPAL1	HbPAL2	HbPAL3	HbPAL4	HbPAL5	HbPAL6	HbPAL7	HbPAL8
HbPAL1	100.00							
HbPAL2	92.97	100.00						
HbPAL3	80.85	80.36	100.00					
HbPAL4	79.15	78.68	91.30	100.00				
HbPAL5	86.18	86.61	82.34	80.17	100.00			
HbPAL6	84.99	84.91	81.92	79.66	93.16	100.00		
HbPAL7	62.05	62.54	62.68	61.82	63.77	62.70	100.00	
HbPAL8	55.64	56.12	55.73	54.95	57.65	56.67	84.42	100.00

Table S2. List of *cis*-acting elements identified in *HbPALs* promoters.

Promotor	Site Name	Sequence	Position	Matrix score.	Strand	Organism	Function
HbPAL1	AAGAA-motif	gGTAAAGAAA	337	9	-	Avena sativa	
HbPAL1	AC-I	GCTTACCTACCA	518	11	+	Arabidopsis thaliana	
HbPAL1	AC-II	CCACCAACCCCC	356	11	+	Phaseolus vulgaris	
HbPAL1	AE-box	AGAAACAA	1174	8	-	Arabidopsis thaliana	part of a module for light response
HbPAL1	as-1	TGACG	725	5	-	Arabidopsis thaliana	
HbPAL1	as-1	TGACG	944	5	+	Arabidopsis thaliana	
HbPAL1	AT~TATA-box	TATATA	44	6	+	Arabidopsis thaliana	
HbPAL1	AT~TATA-box	TATATA	1183	6	-	Arabidopsis thaliana	
HbPAL1	Box 4	ATTAAT	85	6	+	Petroselinum crispum	part of a conserved DNA module involved in light responsiveness
HbPAL1	Box 4	ATTAAT	149	6	+	Petroselinum crispum	part of a conserved DNA module involved in light responsiveness
HbPAL1	Box 4	ATTAAT	229	6	+	Petroselinum crispum	part of a conserved DNA module involved in light responsiveness
HbPAL1	Box 4	ATTAAT	469	6	+	Petroselinum crispum	part of a conserved DNA module involved in light responsiveness
HbPAL1	Box 4	ATTAAT	1497	6	-	Petroselinum crispum	part of a conserved DNA module involved in light responsiveness
HbPAL1	Box 4	ATTAAT	1513	6	-	Petroselinum crispum	part of a conserved DNA module involved in light responsiveness
HbPAL1	Box 4	ATTAAT	1524	6	-	Petroselinum crispum	part of a conserved DNA module involved in light responsiveness
HbPAL1	Box 4	ATTAAT	1592	6	-	Petroselinum crispum	part of a conserved DNA module involved in light responsiveness
HbPAL1	Box 4	ATTAAT	1809	6	-	Petroselinum crispum	part of a conserved DNA module involved in light responsiveness
HbPAL1	Box 4	ATTAAT	1950	6	-	Petroselinum crispum	part of a conserved DNA module involved in light responsiveness
HbPAL1	Box III	atCATTTTCACt	885	11	-	Pisum sativum	protein binding site
HbPAL1	box S	AGCCACC	897	7	-	Arabidopsis thaliana	
HbPAL1	CAAT-box	CAAT	153	4	-	Nicotiana glutinosa	
HbPAL1	CAAT-box	CAAAT	202	5	-	Pisum sativum	common cis-acting element in promoter and enhancer regions
HbPAL1	CAAT-box	CAAT	274	4	+	Nicotiana glutinosa	

HbPAL1	CAAT-box	TGCCAAC	307	7	+	Petunia hybrida	common cis-acting element in promoter and enhancer regions
HbPAL1	CAAT-box	CAAT	325	4	+	Nicotiana glutinosa	
HbPAL1	CAAT-box	CAAAT	396	5	+	Pisum sativum	common cis-acting element in promoter and enhancer regions
HbPAL1	CAAT-box	CAAT	458	4	+	Nicotiana glutinosa	
HbPAL1	CAAT-box	CAAT	553	4	+	Nicotiana glutinosa	
HbPAL1	CAAT-box	CAAT	557	4	+	Nicotiana glutinosa	
HbPAL1	CAAT-box	CAAT	559	4	-	Nicotiana glutinosa	
HbPAL1	CAAT-box	CAAAT	577	5	-	Pisum sativum	common cis-acting element in promoter and enhancer regions
HbPAL1	CAAT-box	CAAAT	740	5	-	Pisum sativum	common cis-acting element in promoter and enhancer regions
HbPAL1	CAAT-box	CAAT	745	4	-	Nicotiana glutinosa	
HbPAL1	CAAT-box	CAAT	860	4	+	Nicotiana glutinosa	
HbPAL1	CAAT-box	CAAT	967	4	-	Nicotiana glutinosa	
HbPAL1	CAAT-box	TGCCAAC	969	7	+	Petunia hybrida	common cis-acting element in promoter and enhancer regions
HbPAL1	CAAT-box	CAAAT	1104	5	-	Pisum sativum	common cis-acting element in promoter and enhancer regions
HbPAL1	CAAT-box	CAAT	1298	4	-	Nicotiana glutinosa	
HbPAL1	CAAT-box	CAAAT	1304	5	-	Pisum sativum	common cis-acting element in promoter and enhancer regions
HbPAL1	CAAT-box	CAAT	1325	4	-	Nicotiana glutinosa	
HbPAL1	CAAT-box	CAAT	1331	4	+	Nicotiana glutinosa	
HbPAL1	CAAT-box	CAAT	1342	4	+	Nicotiana glutinosa	
HbPAL1	CAAT-box	CCAAT	1456	5	-	Arabidopsis thaliana	common cis-acting element in promoter and enhancer regions
HbPAL1	CAAT-box	CAAT	1470	4	+	Nicotiana glutinosa	
HbPAL1	CAAT-box	CAAT	1528	4	-	Nicotiana glutinosa	
HbPAL1	CAAT-box	CAAT	1570	4	-	Nicotiana glutinosa	
HbPAL1	CAAT-box	CAAT	1779	4	+	Nicotiana glutinosa	
HbPAL1	CAAT-box	CAAT	1785	4	+	Nicotiana glutinosa	
HbPAL1	CAAT-box	CAAT	1897	4	+	Nicotiana glutinosa	

HbPAL1	CAAT-box	CAAAT	1945	5	-	Pisum sativum	common cis-acting element in promoter and enhancer regions
HbPAL1	CARE	CAACTCAC	1410	8	-	Oryza sativa	
HbPAL1	CAT-box	GCCACT	463	6	+	Arabidopsis thaliana	cis-acting regulatory element related to meristem expression
HbPAL1	CCAAT-box	CAACGG	972	6	+	Hordeum vulgare	MYBHv1 binding site
HbPAL1	CCAAT-box	CAACGG	1071	6	+	Hordeum vulgare	MYBHv1 binding site
HbPAL1	CGTCA-motif	CGTCA	725	5	+	Hordeum vulgare	cis-acting regulatory element involved in the MeJA-responsiveness
HbPAL1	CGTCA-motif	CGTCA	944	5	-	Hordeum vulgare	cis-acting regulatory element involved in the MeJA-responsiveness
HbPAL1	DRE core	GCCGAC	1017	6	-	Arabidopsis thaliana	dehydration-responsive element
HbPAL1	ERE	ATTTTAAA	33	8	+	Nicotiana glutinos	ethylene-responsive element
HbPAL1	ERE	ATTTTAAA	79	8	-	Nicotiana glutinos	ethylene-responsive element
HbPAL1	ERE	ATTTTAAA	185	8	+	Nicotiana glutinos	ethylene-responsive element
HbPAL1	ERE	ATTTTAAA	187	8	-	Nicotiana glutinos	ethylene-responsive element
HbPAL1	ERE	ATTTTAAA	214	8	-	Nicotiana glutinos	ethylene-responsive element
HbPAL1	ERE	ATTTTAAA	1887	8	+	Nicotiana glutinos	ethylene-responsive element
HbPAL1	GATA-motif	AAGATAAGATT	1871	10	+	Arabidopsis thaliana	part of a light responsive element
HbPAL1	G-box	CACGAC	583	6	-	Zea mays	cis-acting regulatory element involved in light responsiveness
HbPAL1	G-box	CACGAC	819	6	+	Zea mays	cis-acting regulatory element involved in light responsiveness
HbPAL1	GCN4_motif	TGAGTCA	855	7	+	Oryza sativa	cis-regulatory element involved in endosperm expression
HbPAL1	GCN4_motif	TGAGTCA	1253	7	-	Oryza sativa	cis-regulatory element involved in endosperm expression
HbPAL1	LTR	CCGAAA	1346	6	-	Hordeum vulgare	cis-acting element involved in low-temperature responsiveness
HbPAL1	MBS	CAACTG	566	6	+	Arabidopsis thaliana	MYB binding site involved in drought-inducibility
HbPAL1	MYB	CAACCA	310	6	+	Arabidopsis thaliana	MYB binding site
HbPAL1	MYB	CAACAG	349	6	-	Arabidopsis thaliana	MYB binding site
HbPAL1	MYB	CAACCA	478	6	+	Arabidopsis thaliana	MYB binding site
HbPAL1	MYB	TAACCA	684	6	+	Arabidopsis thaliana	MYB binding site
HbPAL1	MYB	CAACAG	702	6	-	Arabidopsis thaliana	MYB binding site

HbPAL1	Myb	CAACTG	566	6	+	Arabidopsis thaliana	MYB binding site
HbPAL1	MYB recognition site	CCGTTG	972	6	-	Arabidopsis thaliana	MYB binding site
HbPAL1	MYB recognition site	CCGTTG	1071	6	-	Arabidopsis thaliana	MYB binding site
HbPAL1	Myb-binding site	CAACAG	349	6	-	Nicotiana tabacum	MYB binding site
HbPAL1	Myb-binding site	CAACAG	702	6	-	Nicotiana tabacum	MYB binding site
HbPAL1	MYB-like sequence	TAACCA	684	6	+	Arabidopsis thaliana	MYB binding site
HbPAL1	MYC	CAATTG	557	6	+	Arabidopsis thaliana	MYC binding site
HbPAL1	MYC	CATTTG	739	6	+	Arabidopsis thaliana	MYC binding site
HbPAL1	MYC	CATTTG	1303	6	+	Arabidopsis thaliana	MYC binding site
HbPAL1	O2-site	GATGATGTGG	291	9	+	Zea mays	cis-acting regulatory element involved in zein metabolism regulation
HbPAL1	O2-site	GATGA(C/T)(A/G)TG(A/G)	880	8	+	Zea mays	cis-acting regulatory element involved in zein metabolism regulation
HbPAL1	P-box	CCTTTTG	1402	7	+	Oryza sativa	gibberellin-responsive element
HbPAL1	STRE	AGGGG	346	5	-	Arabidopsis thaliana	
HbPAL1	STRE	AGGGG	1315	5	+	Arabidopsis thaliana	
HbPAL1	TATA	TATAAAAT	5	8	-	Arabidopsis thaliana	
HbPAL1	TATA	TATAAAAT	170	8	+	Arabidopsis thaliana	
HbPAL1	TATA	TATAAAAT	242	8	+	Arabidopsis thaliana	
HbPAL1	TATA	TATAAAAT	1879	8	-	Arabidopsis thaliana	
HbPAL1	TATA-box	TATAAAA	6	7	-	Pisum sativum	core promoter element around -30 of transcription start

HbPAL1	TATA-box	TATAAA	7	6	-	Helianthus annuus	core promoter element around -30 of transcription start
HbPAL1	TATA-box	TATAA	8	5	-	Arabidopsis thaliana	core promoter element around -30 of transcription start
HbPAL1	TATA-box	TATA	9	4	+	Arabidopsis thaliana	core promoter element around -30 of transcription start
HbPAL1	TATA-box	TATTTAAA	35	8	-	Arabidopsis thaliana	core promoter element around -30 of transcription start
HbPAL1	TATA-box	ATATAT	43	6	+	Brassica napus	core promoter element around -30 of transcription start
HbPAL1	TATA-box	TATATA	44	6	+	Arabidopsis thaliana	core promoter element around -30 of transcription start
HbPAL1	TATA-box	ATATAT	45	6	+	Brassica napus	core promoter element around -30 of transcription start
HbPAL1	TATA-box	TATA	46	4	+	Arabidopsis thaliana	core promoter element around -30 of transcription start
HbPAL1	TATA-box	ATATAT	69	6	+	Brassica napus	core promoter element around -30 of transcription start
HbPAL1	TATA-box	TATA	70	4	+	Arabidopsis thaliana	core promoter element around -30 of transcription start
HbPAL1	TATA-box	TATAA	169	5	-	Arabidopsis thaliana	core promoter element around -30 of transcription start
HbPAL1	TATA-box	TATA	170	4	+	Arabidopsis thaliana	core promoter element around -30 of transcription start
HbPAL1	TATA-box	ATATAA	193	6	+	Brassica oleracea	core promoter element around -30 of transcription start
HbPAL1	TATA-box	TATA	194	4	+	Arabidopsis thaliana	core promoter element around -30 of transcription start
HbPAL1	TATA-box	ATATAA	220	6	+	Brassica oleracea	core promoter element around -30 of transcription start
HbPAL1	TATA-box	TATA	221	4	+	Arabidopsis thaliana	core promoter element around -30 of transcription start
HbPAL1	TATA-box	ATATAA	241	6	+	Brassica oleracea	core promoter element around -30 of transcription start
HbPAL1	TATA-box	TATA	242	4	+	Arabidopsis thaliana	core promoter element around -30 of transcription start
HbPAL1	TATA-box	taTATAAAtc	256	9	-	Arabidopsis thaliana	core promoter element around -30 of transcription start
HbPAL1	TATA-box	TATTTAAA	258	8	-	Arabidopsis thaliana	core promoter element around -30 of transcription start
HbPAL1	TATA-box	TATACA	494	6	-	Helianthus annuus	core promoter element around -30 of transcription start
HbPAL1	TATA-box	TATA	496	4	+	Arabidopsis thaliana	core promoter element around -30 of transcription start
HbPAL1	TATA-box	TATTTAAA	640	8	+	Arabidopsis thaliana	core promoter element around -30 of transcription start
HbPAL1	TATA-box	TATA	907	4	+	Arabidopsis thaliana	core promoter element around -30 of transcription start
HbPAL1	TATA-box	TATATA	1183	6	-	Arabidopsis thaliana	core promoter element around -30 of transcription start
HbPAL1	TATA-box	TATA	1185	4	-	Arabidopsis thaliana	core promoter element around -30 of transcription start

HbPAL1	TATA-box	TATA	1589	4	-	Arabidopsis thaliana	core promoter element around -30 of transcription start
HbPAL1	TATA-box	ATTATA	1619	6	+	Brassica napus	core promoter element around -30 of transcription start
HbPAL1	TATA-box	TATAA	1620	5	-	Arabidopsis thaliana	core promoter element around -30 of transcription start
HbPAL1	TATA-box	TATA	1621	4	-	Arabidopsis thaliana	core promoter element around -30 of transcription start
HbPAL1	TATA-box	TATA	1627	4	-	Arabidopsis thaliana	core promoter element around -30 of transcription start
HbPAL1	TATA-box	tcTATAAATAgg	1784	11	+	Nicotiana tabacum	core promoter element around -30 of transcription start
HbPAL1	TATA-box	TATTTAAA	1814	8	+	Arabidopsis thaliana	core promoter element around -30 of transcription start
HbPAL1	TATA-box	TATATTTATATTT	1828	12	-	Avena sativa	core promoter element around -30 of transcription start
HbPAL1	TATA-box	ATATAT	1836	6	-	Brassica napus	core promoter element around -30 of transcription start
HbPAL1	TATA-box	TATA	1837	4	-	Arabidopsis thaliana	core promoter element around -30 of transcription start
HbPAL1	TATA-box	TATAAAA	1880	7	-	Pisum sativum	core promoter element around -30 of transcription start
HbPAL1	TATA-box	TATAAA	1881	6	-	Helianthus annuus	core promoter element around -30 of transcription start
HbPAL1	TATA-box	TATAA	1882	5	-	Arabidopsis thaliana	core promoter element around -30 of transcription start
HbPAL1	TATA-box	TATA	1883	4	-	Arabidopsis thaliana	core promoter element around -30 of transcription start
HbPAL1	TATA-box	TATTTAAA	1900	8	-	Arabidopsis thaliana	core promoter element around -30 of transcription start
HbPAL1	TATA-box	ATTATA	1932	6	+	Brassica napus	core promoter element around -30 of transcription start
HbPAL1	TATA-box	TATAA	1933	5	-	Arabidopsis thaliana	core promoter element around -30 of transcription start
HbPAL1	TATA-box	TATA	1934	4	-	Arabidopsis thaliana	core promoter element around -30 of transcription start
HbPAL1	TATA-box	TACAAAA	1987	7	+	Oryza sativa	core promoter element around -30 of transcription start
HbPAL1	TCA-element	CCATCTTTTT	889	10	-	Nicotiana tabacum	cis-acting element involved in salicylic acid responsiveness
HbPAL1	TCA-element	TCAGAAGAGG	1368	9	+	Brassica oleracea	cis-acting element involved in salicylic acid responsiveness
HbPAL1	TCT-motif	TCTTAC	1310	6	-	Arabidopsis thaliana	part of a light responsive element
HbPAL1	TCT-motif	TCTTAC	1694	6	-	Arabidopsis thaliana	part of a light responsive element
HbPAL1	TGACG-motif	TGACG	725	5	-	Hordeum vulgare	cis-acting regulatory element involved in the MeJA-responsiveness
HbPAL1	TGACG-motif	TGACG	944	5	+	Hordeum vulgare	cis-acting regulatory element involved in the MeJA-responsiveness
HbPAL1	W box	TTGACC	921	6	-	Arabidopsis thaliana	WRKY binding site

HbPAL1	W box	TTGACC	1266	6	+	Arabidopsis thaliana	WRKY binding site
HbPAL1	WRE3	CCACCT	489	6	+	Pisum sativum	wound-responsive element
HbPAL1	WRE3	CCACCT	656	6	+	Pisum sativum	wound-responsive element
HbPAL1	WRE3	CCACCT	956	6	-	Pisum sativum	wound-responsive element
HbPAL1	WRE3	CCACCT	1001	6	-	Pisum sativum	wound-responsive element
HbPAL1	WRE3	CCACCT	1137	6	-	Pisum sativum	wound-responsive element
HbPAL2	AAGAA-motif	GAAAGAA	1616	7	-	Avena sativa	
HbPAL2	ABRE	CACGTG	62	6	+	Arabidopsis thaliana	cis-acting element involved in the abscisic acid responsiveness
HbPAL2	ABRE	ACGTG	63	5	+	Arabidopsis thaliana	cis-acting element involved in the abscisic acid responsiveness
HbPAL2	ABRE	ACGTG	1532	5	+	Arabidopsis thaliana	cis-acting element involved in the abscisic acid responsiveness
HbPAL2	ABRE3a	TACGTG	1531	6	+	Zea mays	cis-acting element involved in the abscisic acid responsiveness
HbPAL2	ABRE4	CACGTA	1531	6	-	Zea mays	cis-acting element involved in the abscisic acid responsiveness
HbPAL2	AC-I	GCTTACCTACCA	1791	11	+	Arabidopsis thaliana	
HbPAL2	as-1	TGACG	39	5	-	Arabidopsis thaliana	
HbPAL2	AT~TATA-box	TATATA	98	6	+	Arabidopsis thaliana	
HbPAL2	AT~TATA-box	TATATA	1114	6	-	Arabidopsis thaliana	
HbPAL2	AT~TATA-box	TATATAAA	1132	8	-	Arabidopsis thaliana	
HbPAL2	AT~TATA-box	TATATA	1134	6	-	Arabidopsis thaliana	
HbPAL2	AT~TATA-box	TATATA	1136	6	-	Arabidopsis thaliana	
HbPAL2	AT~TATA-box	TATATA	1138	6	-	Arabidopsis thaliana	
HbPAL2	AT~TATA-box	TATATA	1198	6	-	Arabidopsis thaliana	
HbPAL2	AT~TATA-box	TATATA	1504	6	-	Arabidopsis thaliana	
HbPAL2	Box 4	ATTAAT	492	6	+	Petroselinum crispum	part of a conserved DNA module involved in light responsiveness
HbPAL2	Box 4	ATTAAT	575	6	+	Petroselinum crispum	part of a conserved DNA module involved in light responsiveness
HbPAL2	Box 4	ATTAAT	730	6	+	Petroselinum crispum	part of a conserved DNA module involved in light responsiveness
HbPAL2	Box 4	ATTAAT	734	6	+	Petroselinum crispum	part of a conserved DNA module involved in light responsiveness

HbPAL2	Box 4	ATTAAT	738	6	+	Petroselinum crispum	part of a conserved DNA module involved in light responsiveness
HbPAL2	Box 4	ATTAAT	742	6	+	Petroselinum crispum	part of a conserved DNA module involved in light responsiveness
HbPAL2	Box 4	ATTAAT	746	6	+	Petroselinum crispum	part of a conserved DNA module involved in light responsiveness
HbPAL2	Box 4	ATTAAT	750	6	+	Petroselinum crispum	part of a conserved DNA module involved in light responsiveness
HbPAL2	Box 4	ATTAAT	930	6	+	Petroselinum crispum	part of a conserved DNA module involved in light responsiveness
HbPAL2	Box 4	ATTAAT	1100	6	-	Petroselinum crispum	part of a conserved DNA module involved in light responsiveness
HbPAL2	Box 4	ATTAAT	1210	6	-	Petroselinum crispum	part of a conserved DNA module involved in light responsiveness
HbPAL2	Box 4	ATTAAT	1332	6	-	Petroselinum crispum	part of a conserved DNA module involved in light responsiveness
HbPAL2	Box 4	ATTAAT	1365	6	-	Petroselinum crispum	part of a conserved DNA module involved in light responsiveness
HbPAL2	CAAT-box	CAAT	4	4	+	Nicotiana glutinosa	
HbPAL2	CAAT-box	CAAT	191	4	-	Nicotiana glutinosa	
HbPAL2	CAAT-box	CCAAT	251	5	-	Arabidopsis thaliana	common cis-acting element in promoter and enhancer regions
HbPAL2	CAAT-box	CAAAT	277	5	+	Pisum sativum	common cis-acting element in promoter and enhancer regions
HbPAL2	CAAT-box	CAAT	290	4	-	Nicotiana glutinosa	
HbPAL2	CAAT-box	CCAAT	384	5	-	Arabidopsis thaliana	common cis-acting element in promoter and enhancer regions
HbPAL2	CAAT-box	CAAT	418	4	+	Nicotiana glutinosa	
HbPAL2	CAAT-box	CAAAT	430	5	+	Pisum sativum	common cis-acting element in promoter and enhancer regions
HbPAL2	CAAT-box	CAAT	437	4	+	Nicotiana glutinosa	
HbPAL2	CAAT-box	CAAT	461	4	+	Nicotiana glutinosa	
HbPAL2	CAAT-box	CCAAT	484	5	+	Arabidopsis thaliana	common cis-acting element in promoter and enhancer regions
HbPAL2	CAAT-box	CAAT	485	4	+	Nicotiana glutinosa	
HbPAL2	CAAT-box	CCAAT	489	5	+	Arabidopsis thaliana	common cis-acting element in promoter and enhancer regions
HbPAL2	CAAT-box	CAAT	490	4	+	Nicotiana glutinosa	
HbPAL2	CAAT-box	CAAT	551	4	+	Nicotiana glutinosa	
HbPAL2	CAAT-box	CCAAT	648	5	+	Arabidopsis thaliana	common cis-acting element in promoter and enhancer regions
HbPAL2	CAAT-box	CAAT	649	4	+	Nicotiana glutinosa	

HbPAL2	CAAT-box	CCAAT	671	5	+	Arabidopsis thaliana	common cis-acting element in promoter and enhancer regions
HbPAL2	CAAT-box	CAAT	672	4	+	Nicotiana glutinosa	
HbPAL2	CAAT-box	CCAAT	687	5	+	Arabidopsis thaliana	common cis-acting element in promoter and enhancer regions
HbPAL2	CAAT-box	CAAT	688	4	+	Nicotiana glutinosa	
HbPAL2	CAAT-box	CAAAT	781	5	-	Pisum sativum	common cis-acting element in promoter and enhancer regions
HbPAL2	CAAT-box	CAAT	799	4	+	Nicotiana glutinosa	
HbPAL2	CAAT-box	CAAT	819	4	-	Nicotiana glutinosa	
HbPAL2	CAAT-box	CAAT	911	4	+	Nicotiana glutinosa	
HbPAL2	CAAT-box	CAAT	947	4	+	Nicotiana glutinosa	
HbPAL2	CAAT-box	CAAT	954	4	-	Nicotiana glutinosa	
HbPAL2	CAAT-box	CAAAT	1042	5	+	Pisum sativum	common cis-acting element in promoter and enhancer regions
HbPAL2	CAAT-box	CAAAT	1062	5	+	Pisum sativum	common cis-acting element in promoter and enhancer regions
HbPAL2	CAAT-box	CAAAT	1065	5	-	Pisum sativum	common cis-acting element in promoter and enhancer regions
HbPAL2	CAAT-box	CAAT	1111	4	+	Nicotiana glutinosa	
HbPAL2	CAAT-box	CAAT	1119	4	-	Nicotiana glutinosa	
HbPAL2	CAAT-box	CAAAT	1312	5	+	Pisum sativum	common cis-acting element in promoter and enhancer regions
HbPAL2	CAAT-box	CAAT	1380	4	+	Nicotiana glutinosa	
HbPAL2	CAAT-box	CAAT	1493	4	-	Nicotiana glutinosa	
HbPAL2	CAAT-box	CAAT	1518	4	+	Nicotiana glutinosa	
HbPAL2	CAAT-box	CAAT	1602	4	+	Nicotiana glutinosa	
HbPAL2	CAAT-box	CAAAT	1671	5	+	Pisum sativum	common cis-acting element in promoter and enhancer regions
HbPAL2	CAAT-box	CAAT	1707	4	+	Nicotiana glutinosa	
HbPAL2	CAAT-box	CAAT	1735	4	+	Nicotiana glutinosa	
HbPAL2	CAAT-box	CAAT	1970	4	-	Nicotiana glutinosa	
HbPAL2	CAT-box	GCCACT	1740	6	+	Arabidopsis thaliana	cis-acting regulatory element related to meristem expression
HbPAL2	CGTCA-motif	CGTCA	39	5	+	Hordeum vulgare	cis-acting regulatory element involved in the MeJA-responsiveness

HbPAL2	chs-CMA2a	TCACTTGA	635	8	+	Petroselinum crispum	part of a light responsive element
HbPAL2	circadian	CAAAGATATC	1276	9	+	Lycopersicon esculentum	cis-acting regulatory element involved in circadian control
HbPAL2	circadian	CAAAGATATC	1897	9	+	Lycopersicon esculentum	cis-acting regulatory element involved in circadian control
HbPAL2	ERE	ATTTTAAA	13	8	+	Nicotiana glutinos	ethylene-responsive element
HbPAL2	ERE	ATTTTAAA	154	8	+	Nicotiana glutinos	ethylene-responsive element
HbPAL2	ERE	ATTTTAAA	615	8	+	Nicotiana glutinos	ethylene-responsive element
HbPAL2	GA-motif	ATAGATAA	1400	8	-	Arabidopsis thaliana	part of a light responsive element
HbPAL2	GA-motif	ATAGATAA	1476	8	+	Arabidopsis thaliana	part of a light responsive element
HbPAL2	GARE-motif	TCTGTTG	1345	7	+	Brassica oleracea	gibberellin-responsive element
HbPAL2	GATA-motif	GATAGGA	201	7	+	Arabidopsis thaliana	part of a light responsive element
HbPAL2	GATA-motif	AAGGATAAGG	1785	9	-	Solanum tuberosum	part of a light responsive element
HbPAL2	GATA-motif	GATAGGA	1905	7	-	Arabidopsis thaliana	part of a light responsive element
HbPAL2	G-Box	CACGTG	62	6	+	Pisum sativum	cis-acting regulatory element involved in light responsiveness
HbPAL2	G-box	CACGTG	62	6	+	Arabidopsis thaliana	cis-acting regulatory element involved in light responsiveness
HbPAL2	G-box	TACGTG	1531	6	+	Arabidopsis thaliana	cis-acting regulatory element involved in light responsiveness
HbPAL2	GT1-motif	GGTTAAT	1463	7	+	Avena sativa	light responsive element
HbPAL2	I-box	cCATATCCAAT	642	10	+	Flaveria trinervia	part of a light responsive element
HbPAL2	MBS	CAACTG	1625	6	-	Arabidopsis thaliana	MYB binding site involved in drought-inducibility
HbPAL2	Myb	CAACTG	1625	6	-	Arabidopsis thaliana	MYB binding site
HbPAL2	Myb	TAACTG	1976	6	+	Arabidopsis thaliana	MYB binding site
HbPAL2	MYB	CAACAG	1346	6	-	Arabidopsis thaliana	MYB binding site
HbPAL2	MYB	CAACCA	1636	6	+	Arabidopsis thaliana	MYB binding site
HbPAL2	MYB	CAACCA	1755	6	+	Arabidopsis thaliana	MYB binding site
HbPAL2	Myb-binding	CAACAG	1346	6	-	Nicotiana tabacum	MYB binding site

	site						
HbPAL2	Myc	TCTCTTA	811	7	+	Arabidopsis thaliana	MYC binding site
HbPAL2	Myc	TCTCTTA	1917	7	+	Arabidopsis thaliana	MYC binding site
HbPAL2	MYC	CATGTG	1295	6	-	Arabidopsis thaliana	MYC binding site
HbPAL2	P-box	CCTTTTG	325	7	-	Oryza sativa	gibberellin-responsive element
HbPAL2	STRE	AGGGG	329	5	+	Arabidopsis thaliana	
HbPAL2	TATA-box	ATATAA	55	6	+	Brassica oleracea	core promoter element around -30 of transcription start
HbPAL2	TATA-box	TATA	56	4	+	Arabidopsis thaliana	core promoter element around -30 of transcription start
HbPAL2	TATA-box	ATATAT	74	6	+	Brassica napus	core promoter element around -30 of transcription start
HbPAL2	TATA-box	TATA	75	4	+	Arabidopsis thaliana	core promoter element around -30 of transcription start
HbPAL2	TATA-box	ATTATA	96	6	+	Brassica napus	core promoter element around -30 of transcription start
HbPAL2	TATA-box	TATATAA	97	7	-	Arabidopsis thaliana	core promoter element around -30 of transcription start
HbPAL2	TATA-box	TATATA	98	6	+	Arabidopsis thaliana	core promoter element around -30 of transcription start
HbPAL2	TATA-box	ATATAT	99	6	+	Brassica napus	core promoter element around -30 of transcription start
HbPAL2	TATA-box	TATA	100	4	+	Arabidopsis thaliana	core promoter element around -30 of transcription start
HbPAL2	TATA-box	TATAA	106	5	-	Arabidopsis thaliana	core promoter element around -30 of transcription start
HbPAL2	TATA-box	TATA	107	4	+	Arabidopsis thaliana	core promoter element around -30 of transcription start
HbPAL2	TATA-box	TATA	134	4	+	Arabidopsis thaliana	core promoter element around -30 of transcription start
HbPAL2	TATA-box	TATTTAAA	156	8	-	Arabidopsis thaliana	core promoter element around -30 of transcription start
HbPAL2	TATA-box	ATATAA	161	6	+	Brassica oleracea	core promoter element around -30 of transcription start
HbPAL2	TATA-box	TATA	162	4	+	Arabidopsis thaliana	core promoter element around -30 of transcription start
HbPAL2	TATA-box	TATAAATA	231	8	-	Daucus carota	core promoter element around -30 of transcription start
HbPAL2	TATA-box	TATAAAT	232	7	-	Brassica juncea	core promoter element around -30 of transcription start
HbPAL2	TATA-box	TATAAA	233	6	-	Helianthus annuus	core promoter element around -30 of transcription start
HbPAL2	TATA-box	TATAA	234	5	-	Arabidopsis thaliana	core promoter element around -30 of transcription start
HbPAL2	TATA-box	TATA	235	4	+	Arabidopsis thaliana	core promoter element around -30 of transcription start

HbPAL2	TATA-box	TATAAGAA	262	8	-	Zea mays	core promoter element around -30 of transcription start
HbPAL2	TATA-box	TATAA	265	5	-	Arabidopsis thaliana	core promoter element around -30 of transcription start
HbPAL2	TATA-box	TATA	266	4	+	Arabidopsis thaliana	core promoter element around -30 of transcription start
HbPAL2	TATA-box	TATA	358	4	+	Arabidopsis thaliana	core promoter element around -30 of transcription start
HbPAL2	TATA-box	TATAAAA	366	7	-	Pisum sativum	core promoter element around -30 of transcription start
HbPAL2	TATA-box	TATAAA	367	6	-	Helianthus annuus	core promoter element around -30 of transcription start
HbPAL2	TATA-box	TATAA	368	5	-	Arabidopsis thaliana	core promoter element around -30 of transcription start
HbPAL2	TATA-box	TATA	369	4	+	Arabidopsis thaliana	core promoter element around -30 of transcription start
HbPAL2	TATA-box	ATATAA	585	6	+	Brassica oleracea	core promoter element around -30 of transcription start
HbPAL2	TATA-box	TATA	586	4	+	Arabidopsis thaliana	core promoter element around -30 of transcription start
HbPAL2	TATA-box	TATA	723	4	+	Arabidopsis thaliana	core promoter element around -30 of transcription start
HbPAL2	TATA-box	TATA	981	4	+	Arabidopsis thaliana	core promoter element around -30 of transcription start
HbPAL2	TATA-box	ccTATAAAaa	997	9	+	Arabidopsis thaliana	core promoter element around -30 of transcription start
HbPAL2	TATA-box	TATA	999	4	-	Arabidopsis thaliana	core promoter element around -30 of transcription start
HbPAL2	TATA-box	ATATAT	1113	6	-	Brassica napus	core promoter element around -30 of transcription start
HbPAL2	TATA-box	TATATA	1114	6	-	Arabidopsis thaliana	core promoter element around -30 of transcription start
HbPAL2	TATA-box	ATATAT	1115	6	-	Brassica napus	core promoter element around -30 of transcription start
HbPAL2	TATA-box	TATA	1116	4	-	Arabidopsis thaliana	core promoter element around -30 of transcription start
HbPAL2	TATA-box	TATAAAA	1131	7	-	Pisum sativum	core promoter element around -30 of transcription start
HbPAL2	TATA-box	TATAAA	1132	6	-	Helianthus annuus	core promoter element around -30 of transcription start
HbPAL2	TATA-box	TATATAA	1133	7	-	Arabidopsis thaliana	core promoter element around -30 of transcription start
HbPAL2	TATA-box	TATATTTATATTT	1134	12	+	Avena sativa	core promoter element around -30 of transcription start
HbPAL2	TATA-box	ATATAT	1135	6	-	Brassica napus	core promoter element around -30 of transcription start
HbPAL2	TATA-box	TATATA	1136	6	-	Arabidopsis thaliana	core promoter element around -30 of transcription start
HbPAL2	TATA-box	ATATAT	1137	6	-	Brassica napus	core promoter element around -30 of transcription start
HbPAL2	TATA-box	TATATA	1138	6	-	Arabidopsis thaliana	core promoter element around -30 of transcription start

HbPAL2	TATA-box	ATATAT	1139	6	-	Brassica napus	core promoter element around -30 of transcription start
HbPAL2	TATA-box	TATA	1140	4	-	Arabidopsis thaliana	core promoter element around -30 of transcription start
HbPAL2	TATA-box	TATAAAA	1145	7	-	Pisum sativum	core promoter element around -30 of transcription start
HbPAL2	TATA-box	TATAAA	1146	6	-	Helianthus annuus	core promoter element around -30 of transcription start
HbPAL2	TATA-box	TATAA	1147	5	-	Arabidopsis thaliana	core promoter element around -30 of transcription start
HbPAL2	TATA-box	TATA	1148	4	-	Arabidopsis thaliana	core promoter element around -30 of transcription start
HbPAL2	TATA-box	TATATAA	1197	7	-	Arabidopsis thaliana	core promoter element around -30 of transcription start
HbPAL2	TATA-box	TATATA	1198	6	-	Arabidopsis thaliana	core promoter element around -30 of transcription start
HbPAL2	TATA-box	ATATAT	1199	6	-	Brassica napus	core promoter element around -30 of transcription start
HbPAL2	TATA-box	TATA	1200	4	-	Arabidopsis thaliana	core promoter element around -30 of transcription start
HbPAL2	TATA-box	TATACA	1321	6	-	Helianthus annuus	core promoter element around -30 of transcription start
HbPAL2	TATA-box	TATA	1323	4	-	Arabidopsis thaliana	core promoter element around -30 of transcription start
HbPAL2	TATA-box	ATTATA	1483	6	+	Brassica napus	core promoter element around -30 of transcription start
HbPAL2	TATA-box	TATAA	1484	5	-	Arabidopsis thaliana	core promoter element around -30 of transcription start
HbPAL2	TATA-box	TATA	1485	4	-	Arabidopsis thaliana	core promoter element around -30 of transcription start
HbPAL2	TATA-box	taTATAAAtc	1500	9	-	Arabidopsis thaliana	core promoter element around -30 of transcription start
HbPAL2	TATA-box	ATTATA	1502	6	+	Brassica napus	core promoter element around -30 of transcription start
HbPAL2	TATA-box	TATATAA	1503	7	-	Arabidopsis thaliana	core promoter element around -30 of transcription start
HbPAL2	TATA-box	TATATA	1504	6	-	Arabidopsis thaliana	core promoter element around -30 of transcription start
HbPAL2	TATA-box	ATATAT	1505	6	-	Brassica napus	core promoter element around -30 of transcription start
HbPAL2	TATA-box	TATA	1506	4	-	Arabidopsis thaliana	core promoter element around -30 of transcription start
HbPAL2	TATA-box	TATACA	1535	6	-	Helianthus annuus	core promoter element around -30 of transcription start
HbPAL2	TATA-box	TATA	1537	4	-	Arabidopsis thaliana	core promoter element around -30 of transcription start
HbPAL2	TATA-box	TATTTAAA	1845	8	+	Arabidopsis thaliana	core promoter element around -30 of transcription start
HbPAL2	TATA-box	TATAAA	1940	6	-	Helianthus annuus	core promoter element around -30 of transcription start
HbPAL2	TATA-box	TATAA	1941	5	-	Arabidopsis thaliana	core promoter element around -30 of transcription start

HbPAL2	TATA-box	TATA	1942	4	-	Arabidopsis thaliana	core promoter element around -30 of transcription start
HbPAL2	TCCC-motif	TCTCCCT	1771	7	+	Spinacia oleracea	part of a light responsive element
HbPAL2	TCT-motif	TCTTAC	813	6	+	Arabidopsis thaliana	part of a light responsive element
HbPAL2	TCT-motif	TCTTAC	1241	6	+	Arabidopsis thaliana	part of a light responsive element
HbPAL2	TGACG-motif	TGACG	39	5	-	Hordeum vulgare	cis-acting regulatory element involved in the MeJA-responsiveness
HbPAL2	W box	TTGACC	783	6	+	Arabidopsis thaliana	WRKY binding site
HbPAL2	WRE3	CCACCT	347	6	+	Pisum sativum	wound-responsive element
HbPAL2	WUN-motif	TAATTACTC	543	9	+	Nicotiana glutinosa	wound-responsive element
HbPAL3	AAGAA-motif	GAAAGAA	405	7	+	Avena sativa	
HbPAL3	ABRE	ACGTG	499	5	-	Arabidopsis thaliana	cis-acting element involved in the abscisic acid responsiveness
HbPAL3	ABRE	CACGTG	911	6	+	Arabidopsis thaliana	cis-acting element involved in the abscisic acid responsiveness
HbPAL3	ABRE	ACGTG	912	5	+	Arabidopsis thaliana	cis-acting element involved in the abscisic acid responsiveness
HbPAL3	ABRE	TACGTGTC	1714	8	+	Oryza sativa	cis-acting element involved in the abscisic acid responsiveness
HbPAL3	ABRE	ACGTG	1715	5	+	Arabidopsis thaliana	cis-acting element involved in the abscisic acid responsiveness
HbPAL3	ABRE3a	TACGTG	1714	6	+	Zea mays	cis-acting element involved in the abscisic acid responsiveness
HbPAL3	ABRE4	CACGTA	1714	6	-	Zea mays	cis-acting element involved in the abscisic acid responsiveness
HbPAL3	ACE	GACACGTATG	1712	9	-	Petroselinum crispum	cis-acting element involved in light responsiveness
HbPAL3	AC-II	TCACCAACCCCC	512	11	-	Populus tremuloides	
HbPAL3	AE-box	AGAAACTT	1897	8	-	Arabidopsis thaliana	part of a module for light response
HbPAL3	ARE	AAACCA	1247	6	+	Zea mays	cis-acting regulatory element essential for the anaerobic induction
HbPAL3	as-1	TGACG	671	5	+	Arabidopsis thaliana	
HbPAL3	as-1	TGACG	1671	5	+	Arabidopsis thaliana	
HbPAL3	AT~ABRE	TACGTGTC	1714	8	+	Arabidopsis thaliana	
HbPAL3	AT~TATA-box	TATATA	827	6	+	Arabidopsis thaliana	
HbPAL3	AT~TATA-box	TATATA	1165	6	-	Arabidopsis thaliana	
HbPAL3	AT~TATA-box	TATATA	1334	6	-	Arabidopsis thaliana	

HbPAL3	AT1-motif	AATTATTTTTTATT	876	13	+	Solanum tuberosum	part of a light responsive module
HbPAL3	Box 4	ATTAAT	182	6	+	Petroselinum crispum	part of a conserved DNA module involved in light responsiveness
HbPAL3	Box 4	ATTAAT	306	6	+	Petroselinum crispum	part of a conserved DNA module involved in light responsiveness
HbPAL3	Box 4	ATTAAT	326	6	+	Petroselinum crispum	part of a conserved DNA module involved in light responsiveness
HbPAL3	Box 4	ATTAAT	472	6	+	Petroselinum crispum	part of a conserved DNA module involved in light responsiveness
HbPAL3	Box 4	ATTAAT	836	6	+	Petroselinum crispum	part of a conserved DNA module involved in light responsiveness
HbPAL3	Box 4	ATTAAT	926	6	+	Petroselinum crispum	part of a conserved DNA module involved in light responsiveness
HbPAL3	Box 4	ATTAAT	1018	6	-	Petroselinum crispum	part of a conserved DNA module involved in light responsiveness
HbPAL3	Box 4	ATTAAT	1154	6	-	Petroselinum crispum	part of a conserved DNA module involved in light responsiveness
HbPAL3	Box 4	ATTAAT	1377	6	-	Petroselinum crispum	part of a conserved DNA module involved in light responsiveness
HbPAL3	CAAT-box	CAAT	60	4	+	Nicotiana glutinosa	
HbPAL3	CAAT-box	CAAT	72	4	+	Nicotiana glutinosa	
HbPAL3	CAAT-box	CAAAT	107	5	-	Pisum sativum	common cis-acting element in promoter and enhancer regions
HbPAL3	CAAT-box	CAAT	157	4	-	Nicotiana glutinosa	
HbPAL3	CAAT-box	CAAT	161	4	+	Nicotiana glutinosa	
HbPAL3	CAAT-box	CAAAT	189	5	-	Pisum sativum	common cis-acting element in promoter and enhancer regions
HbPAL3	CAAT-box	CAAT	254	4	-	Nicotiana glutinosa	
HbPAL3	CAAT-box	CAAT	259	4	+	Nicotiana glutinosa	
HbPAL3	CAAT-box	CAAT	267	4	-	Nicotiana glutinosa	
HbPAL3	CAAT-box	CAAAT	303	5	+	Pisum sativum	common cis-acting element in promoter and enhancer regions
HbPAL3	CAAT-box	CAAT	310	4	-	Nicotiana glutinosa	
HbPAL3	CAAT-box	CAAT	347	4	-	Nicotiana glutinosa	
HbPAL3	CAAT-box	CAAAT	387	5	+	Pisum sativum	common cis-acting element in promoter and enhancer regions
HbPAL3	CAAT-box	CAAAT	464	5	-	Pisum sativum	common cis-acting element in promoter and enhancer regions
HbPAL3	CAAT-box	CAAT	527	4	+	Nicotiana glutinosa	
HbPAL3	CAAT-box	CAAT	532	4	+	Nicotiana glutinosa	

HbPAL3	CAAT-box	CAAAT	547	5	+	Pisum sativum	common cis-acting element in promoter and enhancer regions
HbPAL3	CAAT-box	CAAT	570	4	-	Nicotiana glutinosa	
HbPAL3	CAAT-box	CAAAT	591	5	-	Pisum sativum	common cis-acting element in promoter and enhancer regions
HbPAL3	CAAT-box	CAAT	637	4	+	Nicotiana glutinosa	
HbPAL3	CAAT-box	CAAT	717	4	+	Nicotiana glutinosa	
HbPAL3	CAAT-box	CAAAT	719	5	-	Pisum sativum	common cis-acting element in promoter and enhancer regions
HbPAL3	CAAT-box	CAAT	735	4	+	Nicotiana glutinosa	
HbPAL3	CAAT-box	CAAT	796	4	-	Nicotiana glutinosa	
HbPAL3	CAAT-box	CAAT	816	4	-	Nicotiana glutinosa	
HbPAL3	CAAT-box	CAAT	1006	4	-	Nicotiana glutinosa	
HbPAL3	CAAT-box	CAAAT	1050	5	+	Pisum sativum	common cis-acting element in promoter and enhancer regions
HbPAL3	CAAT-box	CAAAT	1088	5	+	Pisum sativum	common cis-acting element in promoter and enhancer regions
HbPAL3	CAAT-box	CCAAT	1124	5	+	Arabidopsis thaliana	common cis-acting element in promoter and enhancer regions
HbPAL3	CAAT-box	CAAT	1125	4	+	Nicotiana glutinosa	
HbPAL3	CAAT-box	CAAAT	1286	5	-	Pisum sativum	common cis-acting element in promoter and enhancer regions
HbPAL3	CAAT-box	CAAAT	1406	5	-	Pisum sativum	common cis-acting element in promoter and enhancer regions
HbPAL3	CAAT-box	CAAAT	1486	5	+	Pisum sativum	common cis-acting element in promoter and enhancer regions
HbPAL3	CAAT-box	CAAAT	1544	5	-	Pisum sativum	common cis-acting element in promoter and enhancer regions
HbPAL3	CAAT-box	CAAT	1608	4	-	Nicotiana glutinosa	
HbPAL3	CAAT-box	CAACCAACTCC	1656	10	+	Arabidopsis thaliana	common cis-acting element in promoter and enhancer regions
HbPAL3	CAAT-box	CCAAT	1665	5	+	Arabidopsis thaliana	common cis-acting element in promoter and enhancer regions
HbPAL3	CAAT-box	CAAT	1666	4	+	Nicotiana glutinosa	
HbPAL3	CAAT-box	CAAAT	1668	5	-	Pisum sativum	common cis-acting element in promoter and enhancer regions
HbPAL3	CAAT-box	CAAT	1721	4	+	Nicotiana glutinosa	
HbPAL3	CAAT-box	CAAT	1854	4	+	Nicotiana glutinosa	
HbPAL3	CGTCA-motif	CGTCA	671	5	-	Hordeum vulgare	cis-acting regulatory element involved in the MeJA-responsiveness

HbPAL3	CGTCA-motif	CGTCA	1671	5	-	Hordeum vulgare	cis-acting regulatory element involved in the MeJA-responsiveness
HbPAL3	CTAG-motif	ACTAGCAGAA	1239	9	+	Avena sativa	
HbPAL3	ERE	ATTTTAAA	124	8	+	Nicotiana glutinos	ethylene-responsive element
HbPAL3	ERE	ATTTTAAA	1363	8	+	Nicotiana glutinos	ethylene-responsive element
HbPAL3	ERE	ATTTTAAA	1524	8	-	Nicotiana glutinos	ethylene-responsive element
HbPAL3	ERE	ATTTTAAA	1593	8	-	Nicotiana glutinos	ethylene-responsive element
HbPAL3	Gap-box	CAAATGAA(A/G)A	387	9	+	Arabidopsis thaliana	part of a light responsive element
HbPAL3	GATA-motif	AAGGATAAGG	485	9	+	Solanum tuberosum	part of a light responsive element
HbPAL3	G-Box	CACGTT	499	6	+	Pisum sativum	cis-acting regulatory element involved in light responsiveness
HbPAL3	G-Box	CACGTG	911	6	+	Pisum sativum	cis-acting regulatory element involved in light responsiveness
HbPAL3	G-box	CACGTG	911	6	+	Arabidopsis thaliana	cis-acting regulatory element involved in light responsiveness
HbPAL3	G-box	TAACACGTAG	1713	9	-	Brassica oleracea	cis-acting regulatory element involved in light responsiveness
HbPAL3	G-box	TACGTG	1714	6	+	Arabidopsis thaliana	cis-acting regulatory element involved in light responsiveness
HbPAL3	I-box	gGATAAGGTG	487	9	+	Zea mays	part of a light responsive element
HbPAL3	MYB	CAACCA	538	6	+	Arabidopsis thaliana	MYB binding site
HbPAL3	MYB	CAACAG	1948	6	-	Arabidopsis thaliana	MYB binding site
HbPAL3	Myb-binding site	CAACAG	1948	6	-	Nicotiana tabacum	MYB binding site
HbPAL3	MYC	CATTTG	387	6	-	Arabidopsis thaliana	MYC binding site
HbPAL3	STRE	AGGGG	1945	5	-	Arabidopsis thaliana	
HbPAL3	TATA	TATAAAAT	231	8	-	Arabidopsis thaliana	
HbPAL3	TATA	TATAAAAT	1184	8	+	Arabidopsis thaliana	
HbPAL3	TATA-box	ATTATA	62	6	+	Brassica napus	core promoter element around -30 of transcription start
HbPAL3	TATA-box	TATAA	63	5	-	Arabidopsis thaliana	core promoter element around -30 of transcription start
HbPAL3	TATA-box	TATA	64	4	+	Arabidopsis thaliana	core promoter element around -30 of transcription start
HbPAL3	TATA-box	ATTATA	86	6	+	Brassica napus	core promoter element around -30 of transcription start

HbPAL3	TATA-box	TATAA	87	5	-	Arabidopsis thaliana	core promoter element around -30 of transcription start
HbPAL3	TATA-box	TATA	88	4	+	Arabidopsis thaliana	core promoter element around -30 of transcription start
HbPAL3	TATA-box	ATATAA	114	6	+	Brassica oleracea	core promoter element around -30 of transcription start
HbPAL3	TATA-box	TATA	115	4	+	Arabidopsis thaliana	core promoter element around -30 of transcription start
HbPAL3	TATA-box	TATTTAAA	126	8	-	Arabidopsis thaliana	core promoter element around -30 of transcription start
HbPAL3	TATA-box	TATA	135	4	+	Arabidopsis thaliana	core promoter element around -30 of transcription start
HbPAL3	TATA-box	ATTATA	151	6	+	Brassica napus	core promoter element around -30 of transcription start
HbPAL3	TATA-box	TATAA	152	5	-	Arabidopsis thaliana	core promoter element around -30 of transcription start
HbPAL3	TATA-box	TATA	153	4	+	Arabidopsis thaliana	core promoter element around -30 of transcription start
HbPAL3	TATA-box	ATTATA	163	6	+	Brassica napus	core promoter element around -30 of transcription start
HbPAL3	TATA-box	TATAA	164	5	-	Arabidopsis thaliana	core promoter element around -30 of transcription start
HbPAL3	TATA-box	TATA	165	4	+	Arabidopsis thaliana	core promoter element around -30 of transcription start
HbPAL3	TATA-box	ATATAA	196	6	+	Brassica oleracea	core promoter element around -30 of transcription start
HbPAL3	TATA-box	TATA	197	4	+	Arabidopsis thaliana	core promoter element around -30 of transcription start
HbPAL3	TATA-box	TATAAAA	211	7	-	Pisum sativum	core promoter element around -30 of transcription start
HbPAL3	TATA-box	TATAAA	212	6	-	Helianthus annuus	core promoter element around -30 of transcription start
HbPAL3	TATA-box	TATAA	213	5	-	Arabidopsis thaliana	core promoter element around -30 of transcription start
HbPAL3	TATA-box	TATA	214	4	+	Arabidopsis thaliana	core promoter element around -30 of transcription start
HbPAL3	TATA-box	TATAAAA	232	7	-	Pisum sativum	core promoter element around -30 of transcription start
HbPAL3	TATA-box	TATAAA	233	6	-	Helianthus annuus	core promoter element around -30 of transcription start
HbPAL3	TATA-box	TATAA	234	5	-	Arabidopsis thaliana	core promoter element around -30 of transcription start
HbPAL3	TATA-box	TATA	235	4	+	Arabidopsis thaliana	core promoter element around -30 of transcription start
HbPAL3	TATA-box	ATATAA	271	6	+	Brassica oleracea	core promoter element around -30 of transcription start
HbPAL3	TATA-box	TATA	272	4	+	Arabidopsis thaliana	core promoter element around -30 of transcription start
HbPAL3	TATA-box	TACAAAA	398	7	+	Oryza sativa	core promoter element around -30 of transcription start
HbPAL3	TATA-box	ATATAT	644	6	+	Brassica napus	core promoter element around -30 of transcription start

HbPAL3	TATA-box	TATA	645	4	+	Arabidopsis thaliana	core promoter element around -30 of transcription start
HbPAL3	TATA-box	ATATAT	826	6	+	Brassica napus	core promoter element around -30 of transcription start
HbPAL3	TATA-box	TATATA	827	6	+	Arabidopsis thaliana	core promoter element around -30 of transcription start
HbPAL3	TATA-box	ATATAA	828	6	+	Brassica oleracea	core promoter element around -30 of transcription start
HbPAL3	TATA-box	TATA	829	4	+	Arabidopsis thaliana	core promoter element around -30 of transcription start
HbPAL3	TATA-box	ATATAA	852	6	+	Brassica oleracea	core promoter element around -30 of transcription start
HbPAL3	TATA-box	TATA	853	4	+	Arabidopsis thaliana	core promoter element around -30 of transcription start
HbPAL3	TATA-box	ATATAA	857	6	+	Brassica oleracea	core promoter element around -30 of transcription start
HbPAL3	TATA-box	TATA	858	4	+	Arabidopsis thaliana	core promoter element around -30 of transcription start
HbPAL3	TATA-box	ATATAA	872	6	+	Brassica oleracea	core promoter element around -30 of transcription start
HbPAL3	TATA-box	TATA	873	4	+	Arabidopsis thaliana	core promoter element around -30 of transcription start
HbPAL3	TATA-box	TACATAAA	884	8	-	Oryza sativa	core promoter element around -30 of transcription start
HbPAL3	TATA-box	TATACA	888	6	-	Helianthus annuus	core promoter element around -30 of transcription start
HbPAL3	TATA-box	TATA	890	4	+	Arabidopsis thaliana	core promoter element around -30 of transcription start
HbPAL3	TATA-box	ATATAT	897	6	+	Brassica napus	core promoter element around -30 of transcription start
HbPAL3	TATA-box	TATA	898	4	+	Arabidopsis thaliana	core promoter element around -30 of transcription start
HbPAL3	TATA-box	ATTATA	930	6	+	Brassica napus	core promoter element around -30 of transcription start
HbPAL3	TATA-box	TATAA	931	5	-	Arabidopsis thaliana	core promoter element around -30 of transcription start
HbPAL3	TATA-box	TATA	932	4	+	Arabidopsis thaliana	core promoter element around -30 of transcription start
HbPAL3	TATA-box	TATACA	952	6	-	Helianthus annuus	core promoter element around -30 of transcription start
HbPAL3	TATA-box	TATA	954	4	+	Arabidopsis thaliana	core promoter element around -30 of transcription start
HbPAL3	TATA-box	TATAAAA	985	7	-	Pisum sativum	core promoter element around -30 of transcription start
HbPAL3	TATA-box	TATAAA	986	6	-	Helianthus annuus	core promoter element around -30 of transcription start
HbPAL3	TATA-box	TATAA	987	5	-	Arabidopsis thaliana	core promoter element around -30 of transcription start
HbPAL3	TATA-box	TATA	988	4	+	Arabidopsis thaliana	core promoter element around -30 of transcription start
HbPAL3	TATA-box	ATTATA	992	6	+	Brassica napus	core promoter element around -30 of transcription start

HbPAL3	TATA-box	TATAA	993	5	-	Arabidopsis thaliana	core promoter element around -30 of transcription start
HbPAL3	TATA-box	TATA	994	4	+	Arabidopsis thaliana	core promoter element around -30 of transcription start
HbPAL3	TATA-box	ATATAA	1010	6	+	Brassica oleracea	core promoter element around -30 of transcription start
HbPAL3	TATA-box	TATA	1011	4	-	Arabidopsis thaliana	core promoter element around -30 of transcription start
HbPAL3	TATA-box	TATAAAT	1094	7	-	Brassica juncea	core promoter element around -30 of transcription start
HbPAL3	TATA-box	TATAAA	1095	6	-	Helianthus annuus	core promoter element around -30 of transcription start
HbPAL3	TATA-box	TATAA	1096	5	-	Arabidopsis thaliana	core promoter element around -30 of transcription start
HbPAL3	TATA-box	TATA	1097	4	-	Arabidopsis thaliana	core promoter element around -30 of transcription start
HbPAL3	TATA-box	TATAA	1134	5	-	Arabidopsis thaliana	core promoter element around -30 of transcription start
HbPAL3	TATA-box	TATA	1135	4	-	Arabidopsis thaliana	core promoter element around -30 of transcription start
HbPAL3	TATA-box	TATAA	1141	5	-	Arabidopsis thaliana	core promoter element around -30 of transcription start
HbPAL3	TATA-box	TATA	1142	4	-	Arabidopsis thaliana	core promoter element around -30 of transcription start
HbPAL3	TATA-box	ATATAT	1164	6	-	Brassica napus	core promoter element around -30 of transcription start
HbPAL3	TATA-box	TATATA	1165	6	-	Arabidopsis thaliana	core promoter element around -30 of transcription start
HbPAL3	TATA-box	ATATAT	1166	6	-	Brassica napus	core promoter element around -30 of transcription start
HbPAL3	TATA-box	TATA	1167	4	-	Arabidopsis thaliana	core promoter element around -30 of transcription start
HbPAL3	TATA-box	TATAAAT	1181	7	-	Brassica juncea	core promoter element around -30 of transcription start
HbPAL3	TATA-box	TATAAA	1182	6	-	Helianthus annuus	core promoter element around -30 of transcription start
HbPAL3	TATA-box	TATAA	1183	5	-	Arabidopsis thaliana	core promoter element around -30 of transcription start
HbPAL3	TATA-box	TATA	1184	4	-	Arabidopsis thaliana	core promoter element around -30 of transcription start
HbPAL3	TATA-box	TATTTAAA	1201	8	+	Arabidopsis thaliana	core promoter element around -30 of transcription start
HbPAL3	TATA-box	TATTTAAA	1203	8	-	Arabidopsis thaliana	core promoter element around -30 of transcription start
HbPAL3	TATA-box	TATAAGAA	1330	8	-	Zea mays	core promoter element around -30 of transcription start
HbPAL3	TATA-box	TATATAA	1333	7	-	Arabidopsis thaliana	core promoter element around -30 of transcription start
HbPAL3	TATA-box	TATATA	1334	6	-	Arabidopsis thaliana	core promoter element around -30 of transcription start
HbPAL3	TATA-box	ATATAT	1335	6	-	Brassica napus	core promoter element around -30 of transcription start

HbPAL3	TATA-box	TATA	1336	4	-	Arabidopsis thaliana	core promoter element around -30 of transcription start
HbPAL3	TATA-box	TATTTAAA	1365	8	-	Arabidopsis thaliana	core promoter element around -30 of transcription start
HbPAL3	TATA-box	ATTATA	1372	6	+	Brassica napus	core promoter element around -30 of transcription start
HbPAL3	TATA-box	TATAA	1373	5	-	Arabidopsis thaliana	core promoter element around -30 of transcription start
HbPAL3	TATA-box	TATATTTATATTT	1374	12	+	Avena sativa	core promoter element around -30 of transcription start
HbPAL3	TATA-box	TATTTAAA	1382	8	+	Arabidopsis thaliana	core promoter element around -30 of transcription start
HbPAL3	TATA-box	ccTATAAAaa	1426	10	-	Arabidopsis thaliana	core promoter element around -30 of transcription start
HbPAL3	TATA-box	TATAAAA	1427	7	-	Pisum sativum	core promoter element around -30 of transcription start
HbPAL3	TATA-box	TATAAA	1428	6	-	Helianthus annuus	core promoter element around -30 of transcription start
HbPAL3	TATA-box	TATAA	1429	5	-	Arabidopsis thaliana	core promoter element around -30 of transcription start
HbPAL3	TATA-box	TATA	1430	4	-	Arabidopsis thaliana	core promoter element around -30 of transcription start
HbPAL3	TATA-box	TATAAAA	1465	7	-	Pisum sativum	core promoter element around -30 of transcription start
HbPAL3	TATA-box	TATAAA	1466	6	-	Helianthus annuus	core promoter element around -30 of transcription start
HbPAL3	TATA-box	TATAA	1467	5	-	Arabidopsis thaliana	core promoter element around -30 of transcription start
HbPAL3	TATA-box	TATA	1468	4	-	Arabidopsis thaliana	core promoter element around -30 of transcription start
HbPAL3	TATA-box	TACAAAA	1472	7	-	Oryza sativa	core promoter element around -30 of transcription start
HbPAL3	TATA-box	TATACA	1505	6	-	Helianthus annuus	core promoter element around -30 of transcription start
HbPAL3	TATA-box	TATA	1507	4	-	Arabidopsis thaliana	core promoter element around -30 of transcription start
HbPAL3	TATA-box	TATTTAAA	1829	8	+	Arabidopsis thaliana	core promoter element around -30 of transcription start
HbPAL3	TCA	TCATCTTCAT	1744	9	-	Pisum sativum	
HbPAL3	TCT-motif	TCTTAC	1699	6	+	Arabidopsis thaliana	part of a light responsive element
HbPAL3	TGACG-motif	TGACG	671	5	+	Hordeum vulgare	cis-acting regulatory element involved in the MeJA-responsiveness
HbPAL3	TGACG-motif	TGACG	1671	5	+	Hordeum vulgare	cis-acting regulatory element involved in the MeJA-responsiveness
HbPAL3	WRE3	CCACCT	694	6	+	Pisum sativum	wound-responsive element
HbPAL3	WRE3	CCACCT	1793	6	+	Pisum sativum	wound-responsive element
HbPAL3	WUN-motif	AAATTTCTT	368	9	-	Nicotiana glutinosa	wound-responsive element

HbPAL3	WUN-motif	CCATTTCAA	817	9	-	Nicotiana glutinosa	wound-responsive element
HbPAL3	WUN-motif	AAATTTCTT	1619	9	+	Nicotiana glutinosa	wound-responsive element
HbPAL4	3-AF1 binding site	TAAGAGAGGAA	991	10	+	Solanum tuberosum	light responsive element
HbPAL4	AAAC-motif	CAATCAAAACCT	781	11	+	Spinacia oleracea	light responsive element
HbPAL4	ABRE	CGTACGTGCA	1754	9	+	Hordeum vulgare	cis-acting element involved in the abscisic acid responsiveness
HbPAL4	ABRE	ACGTG	1757	5	+	Arabidopsis thaliana	cis-acting element involved in the abscisic acid responsiveness
HbPAL4	ABRE3a	TACGTG	1756	6	+	Zea mays	cis-acting element involved in the abscisic acid responsiveness
HbPAL4	ABRE4	CACGTA	1756	6	-	Zea mays	cis-acting element involved in the abscisic acid responsiveness
HbPAL4	ARE	AAACCA	395	6	+	Zea mays	cis-acting regulatory element essential for the anaerobic induction
HbPAL4	ARE	AAACCA	892	6	+	Zea mays	cis-acting regulatory element essential for the anaerobic induction
HbPAL4	AT~TATA-box	TATATA	1591	6	-	Arabidopsis thaliana	
HbPAL4	AT~TATA-box	TATATA	1593	6	-	Arabidopsis thaliana	
HbPAL4	AT-rich element	ATAGAAATCAA	533	10	-	Glycine max	binding site of AT-rich DNA binding protein (ATBP-1)
HbPAL4	Box 4	ATTAAT	261	6	+	Petroselinum crispum	part of a conserved DNA module involved in light responsiveness
HbPAL4	Box 4	ATTAAT	527	6	+	Petroselinum crispum	part of a conserved DNA module involved in light responsiveness
HbPAL4	Box 4	ATTAAT	1267	6	-	Petroselinum crispum	part of a conserved DNA module involved in light responsiveness
HbPAL4	CAAT-box	CAAT	50	4	+	Nicotiana glutinosa	
HbPAL4	CAAT-box	CAAT	123	4	+	Nicotiana glutinosa	
HbPAL4	CAAT-box	CAAAT	125	5	-	Pisum sativum	common cis-acting element in promoter and enhancer regions
HbPAL4	CAAT-box	CAAT	132	4	+	Nicotiana glutinosa	
HbPAL4	CAAT-box	CAAT	192	4	+	Nicotiana glutinosa	
HbPAL4	CAAT-box	CAAT	272	4	-	Nicotiana glutinosa	
HbPAL4	CAAT-box	CAAT	277	4	+	Nicotiana glutinosa	
HbPAL4	CAAT-box	CAAT	321	4	-	Nicotiana glutinosa	
HbPAL4	CAAT-box	CAAAT	334	5	+	Pisum sativum	common cis-acting element in promoter and enhancer regions

HbPAL4	CAAT-box	CAAT	590	4	+	Nicotiana glutinosa	
HbPAL4	CAAT-box	CAAAT	633	5	-	Pisum sativum	common cis-acting element in promoter and enhancer regions
HbPAL4	CAAT-box	CAAT	723	4	+	Nicotiana glutinosa	
HbPAL4	CAAT-box	CAAT	760	4	-	Nicotiana glutinosa	
HbPAL4	CAAT-box	CAAT	814	4	+	Nicotiana glutinosa	
HbPAL4	CAAT-box	CAAT	831	4	-	Nicotiana glutinosa	
HbPAL4	CAAT-box	CCAAT	877	5	+	Arabidopsis thaliana	common cis-acting element in promoter and enhancer regions
HbPAL4	CAAT-box	CAAT	878	4	+	Nicotiana glutinosa	
HbPAL4	CAAT-box	CAAT	909	4	+	Nicotiana glutinosa	
HbPAL4	CAAT-box	CAAAT	1002	5	+	Pisum sativum	common cis-acting element in promoter and enhancer regions
HbPAL4	CAAT-box	CAAAT	1077	5	+	Pisum sativum	common cis-acting element in promoter and enhancer regions
HbPAL4	CAAT-box	CCAAT	1325	5	+	Arabidopsis thaliana	common cis-acting element in promoter and enhancer regions
HbPAL4	CAAT-box	CAAT	1326	4	+	Nicotiana glutinosa	
HbPAL4	CAAT-box	CAAAT	1353	5	-	Pisum sativum	common cis-acting element in promoter and enhancer regions
HbPAL4	CAAT-box	CAAAT	1427	5	+	Pisum sativum	common cis-acting element in promoter and enhancer regions
HbPAL4	CAAT-box	CAAAT	1465	5	+	Pisum sativum	common cis-acting element in promoter and enhancer regions
HbPAL4	CAAT-box	CAAT	1472	4	+	Nicotiana glutinosa	
HbPAL4	CAAT-box	CCAAT	1524	5	+	Arabidopsis thaliana	common cis-acting element in promoter and enhancer regions
HbPAL4	CAAT-box	CAAT	1525	4	+	Nicotiana glutinosa	
HbPAL4	CAAT-box	CAAT	1550	4	+	Nicotiana glutinosa	
HbPAL4	CAAT-box	CAAT	1650	4	+	Nicotiana glutinosa	
HbPAL4	CAAT-box	CCAAT	1652	5	-	Arabidopsis thaliana	common cis-acting element in promoter and enhancer regions
HbPAL4	CAAT-box	CCAAT	1703	5	-	Arabidopsis thaliana	common cis-acting element in promoter and enhancer regions
HbPAL4	CAAT-box	CAAT	1730	4	+	Nicotiana glutinosa	
HbPAL4	CAAT-box	CAAT	1762	4	+	Nicotiana glutinosa	
HbPAL4	CAAT-box	CAAT	1780	4	-	Nicotiana glutinosa	

HbPAL4	CAAT-box	CAAT	1804	4	+	Nicotiana glutinosa	
HbPAL4	CAAT-box	CAAAT	1814	5	+	Pisum sativum	common cis-acting element in promoter and enhancer regions
HbPAL4	CAAT-box	CAAT	1857	4	-	Nicotiana glutinosa	
HbPAL4	CAT-box	GCCACT	1248	6	+	Arabidopsis thaliana	cis-acting regulatory element related to meristem expression
HbPAL4	chs-CMA2a	TCACTTGA	1908	8	+	Petroselinum crispum	part of a light responsive element
HbPAL4	circadian	CAAAGATATC	164	9	-	Lycopersicon esculentum	cis-acting regulatory element involved in circadian control
HbPAL4	ERE	ATTTCATA	1345	8	+	Nicotiana glutinos	ethylene-responsive element
HbPAL4	GATA-motif	GATAGGA	1436	7	-	Arabidopsis thaliana	part of a light responsive element
HbPAL4	GATA-motif	AAGGATAAGG	1708	9	+	Solanum tuberosum	part of a light responsive element
HbPAL4	G-box	TACGTG	1756	6	+	Arabidopsis thaliana	cis-acting regulatory element involved in light responsiveness
HbPAL4	GT1-motif	GTGTGTGAA	1494	9	+	Solanum tuberosum	light responsive element
HbPAL4	MBS	CAACTG	899	6	+	Arabidopsis thaliana	MYB binding site involved in drought-inducibility
HbPAL4	MBS	CAACTG	1053	6	+	Arabidopsis thaliana	MYB binding site involved in drought-inducibility
HbPAL4	MRE	AACCTAA	357	7	+	Petroselinum crispum	MYB binding site involved in light responsiveness
HbPAL4	MRE	AACCTAA	735	7	+	Petroselinum crispum	MYB binding site involved in light responsiveness
HbPAL4	MRE	AACCTAA	1381	7	+	Petroselinum crispum	MYB binding site involved in light responsiveness
HbPAL4	MYB	TAACCA	874	6	+	Arabidopsis thaliana	MYB binding site
HbPAL4	MYB	CAACCA	1900	6	+	Arabidopsis thaliana	MYB binding site
HbPAL4	Myb	CAACTG	899	6	+	Arabidopsis thaliana	MYB binding site
HbPAL4	Myb	CAACTG	1053	6	+	Arabidopsis thaliana	MYB binding site
HbPAL4	MYB-like sequence	TAACCA	874	6	+	Arabidopsis thaliana	MYB binding site
HbPAL4	MYC	CATGTG	1288	6	+	Arabidopsis thaliana	MYC binding site
HbPAL4	MYC	CATTTG	1465	6	-	Arabidopsis thaliana	MYC binding site
HbPAL4	MYC	CAATTG	1650	6	-	Arabidopsis thaliana	MYC binding site

HbPAL4	STRE	AGGGG	39	5	-	Arabidopsis thaliana	
HbPAL4	STRE	AGGGG	950	5	+	Arabidopsis thaliana	
HbPAL4	STRE	AGGGG	1365	5	+	Arabidopsis thaliana	
HbPAL4	TATA	TATAAAAT	347	8	+	Arabidopsis thaliana	
HbPAL4	TATA	TATAAAAT	1110	8	-	Arabidopsis thaliana	
HbPAL4	TATA-box	TACAAAA	237	7	+	Oryza sativa	core promoter element around -30 of transcription start
HbPAL4	TATA-box	ATTATA	254	6	+	Brassica napus	core promoter element around -30 of transcription start
HbPAL4	TATA-box	TATAA	255	5	-	Arabidopsis thaliana	core promoter element around -30 of transcription start
HbPAL4	TATA-box	TATA	256	4	+	Arabidopsis thaliana	core promoter element around -30 of transcription start
HbPAL4	TATA-box	TATA	268	4	+	Arabidopsis thaliana	core promoter element around -30 of transcription start
HbPAL4	TATA-box	ATTATA	279	6	+	Brassica napus	core promoter element around -30 of transcription start
HbPAL4	TATA-box	TATAA	280	5	-	Arabidopsis thaliana	core promoter element around -30 of transcription start
HbPAL4	TATA-box	TATA	281	4	+	Arabidopsis thaliana	core promoter element around -30 of transcription start
HbPAL4	TATA-box	TATA	347	4	+	Arabidopsis thaliana	core promoter element around -30 of transcription start
HbPAL4	TATA-box	TATA	391	4	+	Arabidopsis thaliana	core promoter element around -30 of transcription start
HbPAL4	TATA-box	TATAA	485	5	-	Arabidopsis thaliana	core promoter element around -30 of transcription start
HbPAL4	TATA-box	TATA	486	4	+	Arabidopsis thaliana	core promoter element around -30 of transcription start
HbPAL4	TATA-box	TATA	629	4	+	Arabidopsis thaliana	core promoter element around -30 of transcription start
HbPAL4	TATA-box	TATA	676	4	+	Arabidopsis thaliana	core promoter element around -30 of transcription start
HbPAL4	TATA-box	ATATAT	717	6	+	Brassica napus	core promoter element around -30 of transcription start
HbPAL4	TATA-box	TATA	718	4	+	Arabidopsis thaliana	core promoter element around -30 of transcription start
HbPAL4	TATA-box	TATA	792	4	+	Arabidopsis thaliana	core promoter element around -30 of transcription start
HbPAL4	TATA-box	TATAA	920	5	-	Arabidopsis thaliana	core promoter element around -30 of transcription start
HbPAL4	TATA-box	TATA	921	4	+	Arabidopsis thaliana	core promoter element around -30 of transcription start
HbPAL4	TATA-box	TATA	959	4	+	Arabidopsis thaliana	core promoter element around -30 of transcription start
HbPAL4	TATA-box	TATA	1038	4	-	Arabidopsis thaliana	core promoter element around -30 of transcription start

HbPAL4	TATA-box	TATAAAA	1111	7	-	Pisum sativum	core promoter element around -30 of transcription start
HbPAL4	TATA-box	TATAAA	1112	6	-	Helianthus annuus	core promoter element around -30 of transcription start
HbPAL4	TATA-box	TATAA	1113	5	-	Arabidopsis thaliana	core promoter element around -30 of transcription start
HbPAL4	TATA-box	TATA	1114	4	-	Arabidopsis thaliana	core promoter element around -30 of transcription start
HbPAL4	TATA-box	TATAAA	1259	6	-	Helianthus annuus	core promoter element around -30 of transcription start
HbPAL4	TATA-box	TATAA	1260	5	-	Arabidopsis thaliana	core promoter element around -30 of transcription start
HbPAL4	TATA-box	TATA	1261	4	-	Arabidopsis thaliana	core promoter element around -30 of transcription start
HbPAL4	TATA-box	ATATAA	1305	6	+	Brassica oleracea	core promoter element around -30 of transcription start
HbPAL4	TATA-box	TATA	1306	4	-	Arabidopsis thaliana	core promoter element around -30 of transcription start
HbPAL4	TATA-box	ATTATA	1328	6	+	Brassica napus	core promoter element around -30 of transcription start
HbPAL4	TATA-box	TATAA	1329	5	-	Arabidopsis thaliana	core promoter element around -30 of transcription start
HbPAL4	TATA-box	TATA	1330	4	-	Arabidopsis thaliana	core promoter element around -30 of transcription start
HbPAL4	TATA-box	TATA	1485	4	-	Arabidopsis thaliana	core promoter element around -30 of transcription start
HbPAL4	TATA-box	ATTATA	1527	6	+	Brassica napus	core promoter element around -30 of transcription start
HbPAL4	TATA-box	TATAA	1528	5	-	Arabidopsis thaliana	core promoter element around -30 of transcription start
HbPAL4	TATA-box	TATA	1529	4	-	Arabidopsis thaliana	core promoter element around -30 of transcription start
HbPAL4	TATA-box	taTATAAAtc	1589	9	-	Arabidopsis thaliana	core promoter element around -30 of transcription start
HbPAL4	TATA-box	ATATAT	1590	6	-	Brassica napus	core promoter element around -30 of transcription start
HbPAL4	TATA-box	TATATA	1591	6	-	Arabidopsis thaliana	core promoter element around -30 of transcription start
HbPAL4	TATA-box	ATATAT	1592	6	-	Brassica napus	core promoter element around -30 of transcription start
HbPAL4	TATA-box	TATATA	1593	6	-	Arabidopsis thaliana	core promoter element around -30 of transcription start
HbPAL4	TATA-box	ATATAA	1594	6	+	Brassica oleracea	core promoter element around -30 of transcription start
HbPAL4	TATA-box	TATA	1595	4	-	Arabidopsis thaliana	core promoter element around -30 of transcription start
HbPAL4	TATA-box	TATA	1745	4	-	Arabidopsis thaliana	core promoter element around -30 of transcription start
HbPAL4	TATA-box	tcTATAAATAgg	1841	11	-	Nicotiana tabacum	core promoter element around -30 of transcription start
HbPAL4	TATA-box	TATTTAAA	1843	8	+	Arabidopsis thaliana	core promoter element around -30 of transcription start

HbPAL4	TCA	TCATCTTCAT	1785	9	-	Pisum sativum	
HbPAL4	TCCC-motif	TCTCCCT	692	7	+	Spinacia oleracea	part of a light responsive element
HbPAL4	W box	TTGACC	129	6	-	Arabidopsis thaliana	WRKY binding site
HbPAL4	WUN-motif	TAATTACTC	961	9	+	Nicotiana glutinosa	wound-responsive element
HbPAL5	4cl-CMA2b	TCTCACCAACCCCA	1278	13	-	Petroselinum crispum	light responsive element
HbPAL5	AAGAA-motif	GAAAGAA	1362	7	-	Avena sativa	
HbPAL5	AAGAA-motif	GAAAGAA	1366	7	-	Avena sativa	
HbPAL5	ABRE	ACGTG	1320	5	+	Arabidopsis thaliana	cis-acting element involved in the abscisic acid responsiveness
HbPAL5	ABRE	ACGTG	1687	5	+	Arabidopsis thaliana	cis-acting element involved in the abscisic acid responsiveness
HbPAL5	ABRE	GACACGTGGC	1750	9	-	Triticum aestivum	cis-acting element involved in the abscisic acid responsiveness
HbPAL5	ABRE	CACGTG	1752	6	-	Arabidopsis thaliana	cis-acting element involved in the abscisic acid responsiveness
HbPAL5	ABRE	ACGTG	1753	5	+	Arabidopsis thaliana	cis-acting element involved in the abscisic acid responsiveness
HbPAL5	ABRE3a	TACGTG	1686	6	+	Zea mays	cis-acting element involved in the abscisic acid responsiveness
HbPAL5	ABRE4	CACGTA	1686	6	-	Zea mays	cis-acting element involved in the abscisic acid responsiveness
HbPAL5	AC-I	(T/C)C(T/C)(C/T)ACC (T/C)ACC	1281	8.5	-	Phaseolus vulgaris	
HbPAL5	AC-I	(T/C)C(T/C)(C/T)ACC (T/C)ACC	1340	9	+	Phaseolus vulgaris	
HbPAL5	as-1	TGACG	1318	5	+	Arabidopsis thaliana	
HbPAL5	as-1	TGACG	1783	5	+	Arabidopsis thaliana	
HbPAL5	AT~TATA-box	TATATA	158	6	+	Arabidopsis thaliana	
HbPAL5	AT~TATA-box	TATATA	160	6	+	Arabidopsis thaliana	
HbPAL5	AT~TATA-box	TATATA	162	6	+	Arabidopsis thaliana	
HbPAL5	AT~TATA-box	TATATA	174	6	+	Arabidopsis thaliana	
HbPAL5	AT~TATA-box	TATATA	902	6	+	Arabidopsis thaliana	
HbPAL5	AT~TATA-box	TATATA	904	6	+	Arabidopsis thaliana	

HbPAL5	AT~TATA-box	TATATA	1230	6	-	Arabidopsis thaliana	
HbPAL5	AT~TATA-box	TATATA	1482	6	-	Arabidopsis thaliana	
HbPAL5	AT~TATA-box	TATATA	1484	6	-	Arabidopsis thaliana	
HbPAL5	AT~TATA-box	TATATA	1486	6	-	Arabidopsis thaliana	
HbPAL5	AT~TATA-box	TATATA	1488	6	-	Arabidopsis thaliana	
HbPAL5	AT~TATA-box	TATATA	1490	6	-	Arabidopsis thaliana	
HbPAL5	AT~TATA-box	TATATA	1492	6	-	Arabidopsis thaliana	
HbPAL5	AT~TATA-box	TATATA	1494	6	-	Arabidopsis thaliana	
HbPAL5	AT~TATA-box	TATATA	1496	6	-	Arabidopsis thaliana	
HbPAL5	AT~TATA-box	TATATAAA	1591	8	-	Arabidopsis thaliana	
HbPAL5	AT~TATA-box	TATATA	1593	6	-	Arabidopsis thaliana	
HbPAL5	AT1-motif	AATTATTTTTTATT	1001	13	-	Solanum tuberosum	part of a light responsive module
HbPAL5	Box 4	ATTAAT	7	6	+	Petroselinum crispum	part of a conserved DNA module involved in light responsiveness
HbPAL5	Box 4	ATTAAT	58	6	+	Petroselinum crispum	part of a conserved DNA module involved in light responsiveness
HbPAL5	Box 4	ATTAAT	224	6	+	Petroselinum crispum	part of a conserved DNA module involved in light responsiveness
HbPAL5	Box 4	ATTAAT	248	6	+	Petroselinum crispum	part of a conserved DNA module involved in light responsiveness
HbPAL5	Box 4	ATTAAT	354	6	+	Petroselinum crispum	part of a conserved DNA module involved in light responsiveness
HbPAL5	Box 4	ATTAAT	1025	6	-	Petroselinum crispum	part of a conserved DNA module involved in light responsiveness
HbPAL5	Box 4	ATTAAT	1059	6	-	Petroselinum crispum	part of a conserved DNA module involved in light responsiveness
HbPAL5	Box 4	ATTAAT	1181	6	-	Petroselinum crispum	part of a conserved DNA module involved in light responsiveness
HbPAL5	Box 4	ATTAAT	1189	6	-	Petroselinum crispum	part of a conserved DNA module involved in light responsiveness
HbPAL5	Box 4	ATTAAT	1237	6	-	Petroselinum crispum	part of a conserved DNA module involved in light responsiveness
HbPAL5	Box II	TGGTAATAA	665	9	-	Solanum tuberosum	part of a light responsive element
HbPAL5	CAAT-box	CAAT	53	4	-	Nicotiana glutinosa	
HbPAL5	CAAT-box	CAAT	91	4	-	Nicotiana glutinosa	
HbPAL5	CAAT-box	CAAAT	207	5	+	Pisum sativum	common cis-acting element in promoter and enhancer regions

HbPAL5	CAAT-box	CAAT	244	4	-	Nicotiana glutinosa	
HbPAL5	CAAT-box	CAAT	293	4	+	Nicotiana glutinosa	
HbPAL5	CAAT-box	CAAAT	295	5	-	Pisum sativum	common cis-acting element in promoter and enhancer regions
HbPAL5	CAAT-box	CAAAT	300	5	-	Pisum sativum	common cis-acting element in promoter and enhancer regions
HbPAL5	CAAT-box	CAAT	305	4	+	Nicotiana glutinosa	
HbPAL5	CAAT-box	CCAAT	335	5	+	Arabidopsis thaliana	common cis-acting element in promoter and enhancer regions
HbPAL5	CAAT-box	CAAT	336	4	+	Nicotiana glutinosa	
HbPAL5	CAAT-box	CAAT	384	4	-	Nicotiana glutinosa	
HbPAL5	CAAT-box	CAAT	428	4	-	Nicotiana glutinosa	
HbPAL5	CAAT-box	CAAT	440	4	+	Nicotiana glutinosa	
HbPAL5	CAAT-box	CAAT	591	4	+	Nicotiana glutinosa	
HbPAL5	CAAT-box	CAAAT	617	5	-	Pisum sativum	common cis-acting element in promoter and enhancer regions
HbPAL5	CAAT-box	CAAT	646	4	+	Nicotiana glutinosa	
HbPAL5	CAAT-box	CAAT	689	4	-	Nicotiana glutinosa	
HbPAL5	CAAT-box	CAAT	728	4	+	Nicotiana glutinosa	
HbPAL5	CAAT-box	CAAT	784	4	+	Nicotiana glutinosa	
HbPAL5	CAAT-box	CAAAT	791	5	-	Pisum sativum	common cis-acting element in promoter and enhancer regions
HbPAL5	CAAT-box	CAAAT	804	5	+	Pisum sativum	common cis-acting element in promoter and enhancer regions
HbPAL5	CAAT-box	CAAAT	850	5	+	Pisum sativum	common cis-acting element in promoter and enhancer regions
HbPAL5	CAAT-box	CAAT	861	4	-	Nicotiana glutinosa	
HbPAL5	CAAT-box	CAAAT	875	5	-	Pisum sativum	common cis-acting element in promoter and enhancer regions
HbPAL5	CAAT-box	CAAAT	881	5	-	Pisum sativum	common cis-acting element in promoter and enhancer regions
HbPAL5	CAAT-box	CAAAT	1029	5	-	Pisum sativum	common cis-acting element in promoter and enhancer regions
HbPAL5	CAAT-box	CAAT	1048	4	+	Nicotiana glutinosa	
HbPAL5	CAAT-box	CAAT	1063	4	-	Nicotiana glutinosa	
HbPAL5	CAAT-box	CAAAT	1083	5	-	Pisum sativum	common cis-acting element in promoter and enhancer regions

HbPAL5	CAAT-box	CAAAT	1089	5	+	Pisum sativum	common cis-acting element in promoter and enhancer regions
HbPAL5	CAAT-box	CAAT	1098	4	+	Nicotiana glutinosa	
HbPAL5	CAAT-box	CAAT	1162	4	+	Nicotiana glutinosa	
HbPAL5	CAAT-box	CAAAT	1533	5	+	Pisum sativum	common cis-acting element in promoter and enhancer regions
HbPAL5	CAAT-box	CCAAT	1556	5	+	Arabidopsis thaliana	common cis-acting element in promoter and enhancer regions
HbPAL5	CAAT-box	CAAT	1557	4	+	Nicotiana glutinosa	
HbPAL5	CAAT-box	CAAT	1624	4	+	Nicotiana glutinosa	
HbPAL5	CAAT-box	CAAT	1658	4	+	Nicotiana glutinosa	
HbPAL5	CAAT-box	CAAAT	1668	5	-	Pisum sativum	common cis-acting element in promoter and enhancer regions
HbPAL5	CAAT-box	CAAT	1682	4	+	Nicotiana glutinosa	
HbPAL5	CAAT-box	CAAT	1741	4	+	Nicotiana glutinosa	
HbPAL5	CAAT-box	CCAAT	1830	5	+	Arabidopsis thaliana	common cis-acting element in promoter and enhancer regions
HbPAL5	CAAT-box	CAAT	1831	4	+	Nicotiana glutinosa	
HbPAL5	CAT-box	GCCACT	1885	6	+	Arabidopsis thaliana	cis-acting regulatory element related to meristem expression
HbPAL5	CCAAT-box	CAACGG	1791	6	+	Hordeum vulgare	MYBHv1 binding site
HbPAL5	CGTCA-motif	CGTCA	1318	5	-	Hordeum vulgare	cis-acting regulatory element involved in the MeJA-responsiveness
HbPAL5	CGTCA-motif	CGTCA	1783	5	-	Hordeum vulgare	cis-acting regulatory element involved in the MeJA-responsiveness
HbPAL5	chs-CMA1a	TTACTTAA	416	8	-	Daucus carota	part of a light responsive element
HbPAL5	ERE	ATTTTAAA	681	8	+	Nicotiana glutinos	ethylene-responsive element
HbPAL5	ERE	ATTTTAAA	683	8	-	Nicotiana glutinos	ethylene-responsive element
HbPAL5	ERE	ATTTCATA	890	8	-	Nicotiana glutinos	ethylene-responsive element
HbPAL5	ERE	ATTTCATA	1432	8	+	Nicotiana glutinos	ethylene-responsive element
HbPAL5	GA-motif	ATAGATAA	165	8	+	Arabidopsis thaliana	part of a light responsive element
HbPAL5	G-box	CACGTC	1319	6	-	Zea mays	cis-acting regulatory element involved in light responsiveness
HbPAL5	G-box	TACGTG	1686	6	+	Arabidopsis thaliana	cis-acting regulatory element involved in light responsiveness
HbPAL5	G-box	CACGTG	1752	6	-	Arabidopsis thaliana	cis-acting regulatory element involved in light responsiveness

HbPAL5	G-Box	CACGTG	1752	6	-	Pisum sativum	cis-acting regulatory element involved in light responsiveness
HbPAL5	GCN4_motif	TGAGTCA	742	7	+	Oryza sativa	cis-regulatory element involved in endosperm expression
HbPAL5	GT1-motif	GGTTAA	534	6	+	Arabidopsis thaliana	light responsive element
HbPAL5	I-box	gGATAAGGTG	548	9	+	Zea mays	part of a light responsive element
HbPAL5	MYB	CAACCA	1280	6	-	Arabidopsis thaliana	MYB binding site
HbPAL5	MYB	CAACCA	1811	6	+	Arabidopsis thaliana	MYB binding site
HbPAL5	MYB	CAACCA	1868	6	+	Arabidopsis thaliana	MYB binding site
HbPAL5	MYB	CAACCA	1877	6	-	Arabidopsis thaliana	MYB binding site
HbPAL5	MYB recognition site	CCGTTG	1791	6	-	Arabidopsis thaliana	MYB binding site
HbPAL5	MYC	CATTTG	790	6	+	Arabidopsis thaliana	MYC binding site
HbPAL5	MYC	CATTTG	804	6	-	Arabidopsis thaliana	MYC binding site
HbPAL5	O2-site	GATGA(C/T)(A/G)TG (A/G)	1316	8	+	Zea mays	cis-acting regulatory element involved in zein metabolism regulation
HbPAL5	STRE	AGGGG	824	5	-	Arabidopsis thaliana	
HbPAL5	STRE	AGGGG	1349	5	-	Arabidopsis thaliana	
HbPAL5	STRE	AGGGG	1837	5	-	Arabidopsis thaliana	
HbPAL5	STRE	AGGGG	1905	5	-	Arabidopsis thaliana	
HbPAL5	TATA	TATAAAAT	1211	8	+	Arabidopsis thaliana	
HbPAL5	TATA	TATAAAAT	1589	8	-	Arabidopsis thaliana	
HbPAL5	TATA	TATAAAAT	1603	8	-	Arabidopsis thaliana	
HbPAL5	TATA-box	TATA	122	4	+	Arabidopsis thaliana	core promoter element around -30 of transcription start
HbPAL5	TATA-box	taTATAAAtc	156	9	-	Arabidopsis thaliana	core promoter element around -30 of transcription start
HbPAL5	TATA-box	ATATAT	157	6	+	Brassica napus	core promoter element around -30 of transcription start
HbPAL5	TATA-box	TATATA	158	6	+	Arabidopsis thaliana	core promoter element around -30 of transcription start
HbPAL5	TATA-box	ATATAT	159	6	+	Brassica napus	core promoter element around -30 of transcription start

HbPAL5	TATA-box	TATATA	160	6	+	Arabidopsis thaliana	core promoter element around -30 of transcription start
HbPAL5	TATA-box	ATATAT	161	6	+	Brassica napus	core promoter element around -30 of transcription start
HbPAL5	TATA-box	TATATA	162	6	+	Arabidopsis thaliana	core promoter element around -30 of transcription start
HbPAL5	TATA-box	TATA	164	4	+	Arabidopsis thaliana	core promoter element around -30 of transcription start
HbPAL5	TATA-box	ATATAT	173	6	+	Brassica napus	core promoter element around -30 of transcription start
HbPAL5	TATA-box	TATATA	174	6	+	Arabidopsis thaliana	core promoter element around -30 of transcription start
HbPAL5	TATA-box	ATATAT	175	6	+	Brassica napus	core promoter element around -30 of transcription start
HbPAL5	TATA-box	TATA	176	4	+	Arabidopsis thaliana	core promoter element around -30 of transcription start
HbPAL5	TATA-box	ATATAA	219	6	+	Brassica oleracea	core promoter element around -30 of transcription start
HbPAL5	TATA-box	TATA	220	4	+	Arabidopsis thaliana	core promoter element around -30 of transcription start
HbPAL5	TATA-box	TATAAA	322	6	-	Helianthus annuus	core promoter element around -30 of transcription start
HbPAL5	TATA-box	TATAA	323	5	-	Arabidopsis thaliana	core promoter element around -30 of transcription start
HbPAL5	TATA-box	TATA	324	4	+	Arabidopsis thaliana	core promoter element around -30 of transcription start
HbPAL5	TATA-box	ATTATA	358	6	+	Brassica napus	core promoter element around -30 of transcription start
HbPAL5	TATA-box	TATAA	359	5	-	Arabidopsis thaliana	core promoter element around -30 of transcription start
HbPAL5	TATA-box	TATA	360	4	+	Arabidopsis thaliana	core promoter element around -30 of transcription start
HbPAL5	TATA-box	ATATAA	399	6	+	Brassica oleracea	core promoter element around -30 of transcription start
HbPAL5	TATA-box	TATA	400	4	+	Arabidopsis thaliana	core promoter element around -30 of transcription start
HbPAL5	TATA-box	ATATAA	442	6	+	Brassica oleracea	core promoter element around -30 of transcription start
HbPAL5	TATA-box	TATA	443	4	+	Arabidopsis thaliana	core promoter element around -30 of transcription start
HbPAL5	TATA-box	TATAAA	451	6	-	Helianthus annuus	core promoter element around -30 of transcription start
HbPAL5	TATA-box	TATAA	452	5	-	Arabidopsis thaliana	core promoter element around -30 of transcription start
HbPAL5	TATA-box	TATA	453	4	+	Arabidopsis thaliana	core promoter element around -30 of transcription start
HbPAL5	TATA-box	TATAA	507	5	-	Arabidopsis thaliana	core promoter element around -30 of transcription start
HbPAL5	TATA-box	TATA	508	4	+	Arabidopsis thaliana	core promoter element around -30 of transcription start
HbPAL5	TATA-box	ATTATA	648	6	+	Brassica napus	core promoter element around -30 of transcription start

HbPAL5	TATA-box	TATAA	649	5	-	Arabidopsis thaliana	core promoter element around -30 of transcription start
HbPAL5	TATA-box	TATA	650	4	+	Arabidopsis thaliana	core promoter element around -30 of transcription start
HbPAL5	TATA-box	ccTATAAAaa	714	9	-	Arabidopsis thaliana	core promoter element around -30 of transcription start
HbPAL5	TATA-box	TATAAAA	715	7	-	Pisum sativum	core promoter element around -30 of transcription start
HbPAL5	TATA-box	TATAAA	716	6	-	Helianthus annuus	core promoter element around -30 of transcription start
HbPAL5	TATA-box	TATAA	717	5	-	Arabidopsis thaliana	core promoter element around -30 of transcription start
HbPAL5	TATA-box	TATA	718	4	+	Arabidopsis thaliana	core promoter element around -30 of transcription start
HbPAL5	TATA-box	TATA	738	4	+	Arabidopsis thaliana	core promoter element around -30 of transcription start
HbPAL5	TATA-box	taTATAAAtc	753	9	-	Arabidopsis thaliana	core promoter element around -30 of transcription start
HbPAL5	TATA-box	TATTTAAA	755	8	-	Arabidopsis thaliana	core promoter element around -30 of transcription start
HbPAL5	TATA-box	TATACA	796	6	-	Helianthus annuus	core promoter element around -30 of transcription start
HbPAL5	TATA-box	TATA	798	4	+	Arabidopsis thaliana	core promoter element around -30 of transcription start
HbPAL5	TATA-box	TATA	870	4	+	Arabidopsis thaliana	core promoter element around -30 of transcription start
HbPAL5	TATA-box	taTATAAAtc	898	9	-	Arabidopsis thaliana	core promoter element around -30 of transcription start
HbPAL5	TATA-box	ATTATA	900	6	+	Brassica napus	core promoter element around -30 of transcription start
HbPAL5	TATA-box	TATATAA	901	7	-	Arabidopsis thaliana	core promoter element around -30 of transcription start
HbPAL5	TATA-box	TATATA	902	6	+	Arabidopsis thaliana	core promoter element around -30 of transcription start
HbPAL5	TATA-box	ATATAT	903	6	+	Brassica napus	core promoter element around -30 of transcription start
HbPAL5	TATA-box	TATATA	904	6	+	Arabidopsis thaliana	core promoter element around -30 of transcription start
HbPAL5	TATA-box	ATATAT	905	6	+	Brassica napus	core promoter element around -30 of transcription start
HbPAL5	TATA-box	TATA	906	4	+	Arabidopsis thaliana	core promoter element around -30 of transcription start
HbPAL5	TATA-box	TATA	959	4	+	Arabidopsis thaliana	core promoter element around -30 of transcription start
HbPAL5	TATA-box	ATTATA	1209	6	+	Brassica napus	core promoter element around -30 of transcription start
HbPAL5	TATA-box	TATAA	1210	5	-	Arabidopsis thaliana	core promoter element around -30 of transcription start
HbPAL5	TATA-box	TATA	1211	4	-	Arabidopsis thaliana	core promoter element around -30 of transcription start
HbPAL5	TATA-box	ATATAT	1229	6	-	Brassica napus	core promoter element around -30 of transcription start

HbPAL5	TATA-box	TATATA	1230	6	-	Arabidopsis thaliana	core promoter element around -30 of transcription start
HbPAL5	TATA-box	ATATAA	1231	6	+	Brassica oleracea	core promoter element around -30 of transcription start
HbPAL5	TATA-box	TATA	1232	4	-	Arabidopsis thaliana	core promoter element around -30 of transcription start
HbPAL5	TATA-box	TACAAAA	1271	7	+	Oryza sativa	core promoter element around -30 of transcription start
HbPAL5	TATA-box	TATA	1399	4	-	Arabidopsis thaliana	core promoter element around -30 of transcription start
HbPAL5	TATA-box	TATAAAT	1403	7	-	Brassica juncea	core promoter element around -30 of transcription start
HbPAL5	TATA-box	TATAAA	1404	6	-	Helianthus annuus	core promoter element around -30 of transcription start
HbPAL5	TATA-box	TATAA	1405	5	-	Arabidopsis thaliana	core promoter element around -30 of transcription start
HbPAL5	TATA-box	TATA	1406	4	-	Arabidopsis thaliana	core promoter element around -30 of transcription start
HbPAL5	TATA-box	ATTATA	1480	6	+	Brassica napus	core promoter element around -30 of transcription start
HbPAL5	TATA-box	TATATAA	1481	7	-	Arabidopsis thaliana	core promoter element around -30 of transcription start
HbPAL5	TATA-box	TATATA	1482	6	-	Arabidopsis thaliana	core promoter element around -30 of transcription start
HbPAL5	TATA-box	ATATAT	1483	6	-	Brassica napus	core promoter element around -30 of transcription start
HbPAL5	TATA-box	TATATA	1484	6	-	Arabidopsis thaliana	core promoter element around -30 of transcription start
HbPAL5	TATA-box	ATATAT	1485	6	-	Brassica napus	core promoter element around -30 of transcription start
HbPAL5	TATA-box	TATATA	1486	6	-	Arabidopsis thaliana	core promoter element around -30 of transcription start
HbPAL5	TATA-box	ATATAT	1487	6	-	Brassica napus	core promoter element around -30 of transcription start
HbPAL5	TATA-box	TATATA	1488	6	-	Arabidopsis thaliana	core promoter element around -30 of transcription start
HbPAL5	TATA-box	ATATAT	1489	6	-	Brassica napus	core promoter element around -30 of transcription start
HbPAL5	TATA-box	TATATA	1490	6	-	Arabidopsis thaliana	core promoter element around -30 of transcription start
HbPAL5	TATA-box	ATATAT	1491	6	-	Brassica napus	core promoter element around -30 of transcription start
HbPAL5	TATA-box	TATATTTATATTT	1492	12	+	Avena sativa	core promoter element around -30 of transcription start
HbPAL5	TATA-box	ATATAT	1493	6	-	Brassica napus	core promoter element around -30 of transcription start
HbPAL5	TATA-box	TATATA	1494	6	-	Arabidopsis thaliana	core promoter element around -30 of transcription start
HbPAL5	TATA-box	ATATAT	1495	6	-	Brassica napus	core promoter element around -30 of transcription start
HbPAL5	TATA-box	TATATA	1496	6	-	Arabidopsis thaliana	core promoter element around -30 of transcription start

HbPAL5	TATA-box	ATATAT	1497	6	-	Brassica napus	core promoter element around -30 of transcription start
HbPAL5	TATA-box	TATA	1498	4	-	Arabidopsis thaliana	core promoter element around -30 of transcription start
HbPAL5	TATA-box	TATA	1579	4	-	Arabidopsis thaliana	core promoter element around -30 of transcription start
HbPAL5	TATA-box	TATAAAA	1590	7	-	Pisum sativum	core promoter element around -30 of transcription start
HbPAL5	TATA-box	TATAAA	1591	6	-	Helianthus annuus	core promoter element around -30 of transcription start
HbPAL5	TATA-box	TATATAA	1592	7	-	Arabidopsis thaliana	core promoter element around -30 of transcription start
HbPAL5	TATA-box	TATATA	1593	6	-	Arabidopsis thaliana	core promoter element around -30 of transcription start
HbPAL5	TATA-box	ATATAA	1594	6	+	Brassica oleracea	core promoter element around -30 of transcription start
HbPAL5	TATA-box	TATA	1595	4	-	Arabidopsis thaliana	core promoter element around -30 of transcription start
HbPAL5	TATA-box	TATAAAA	1604	7	-	Pisum sativum	core promoter element around -30 of transcription start
HbPAL5	TATA-box	TATAAA	1605	6	-	Helianthus annuus	core promoter element around -30 of transcription start
HbPAL5	TATA-box	TATAA	1606	5	-	Arabidopsis thaliana	core promoter element around -30 of transcription start
HbPAL5	TATA-box	TATA	1607	4	-	Arabidopsis thaliana	core promoter element around -30 of transcription start
HbPAL5	TATA-box	ATTATA	1663	6	+	Brassica napus	core promoter element around -30 of transcription start
HbPAL5	TATA-box	TATAA	1664	5	-	Arabidopsis thaliana	core promoter element around -30 of transcription start
HbPAL5	TATA-box	TATA	1665	4	-	Arabidopsis thaliana	core promoter element around -30 of transcription start
HbPAL5	TATA-box	TATTTAAA	1859	8	+	Arabidopsis thaliana	core promoter element around -30 of transcription start
HbPAL5	TCCC-motif	TCTCCCT	855	7	-	Spinacia oleracea	part of a light responsive element
HbPAL5	TCCC-motif	TCTCCCT	1307	7	-	Spinacia oleracea	part of a light responsive element
HbPAL5	TCT-motif	TCTTAC	1969	6	+	Arabidopsis thaliana	part of a light responsive element
HbPAL5	TGACG-motif	TGACG	1318	5	+	Hordeum vulgare	cis-acting regulatory element involved in the MeJA-responsiveness
HbPAL5	TGACG-motif	TGACG	1783	5	+	Hordeum vulgare	cis-acting regulatory element involved in the MeJA-responsiveness
HbPAL5	WUN-motif	AAATTACTA	1584	9	-	Nicotiana glutinosa	wound-responsive element
HbPAL5	WUN-motif	AAATTACT	1585	8	-	Nicotiana glutinosa	wound-responsive element
HbPAL6	AAGAA-motif	GAAAGAA	1505	7	+	Avena sativa	
HbPAL6	AAGAA-motif	GAAAGAA	1544	7	+	Avena sativa	

HbPAL6	A-box	CCGTCC	1662	6	+	Petroselinum crispum	cis-acting regulatory element
HbPAL6	AC-I	(T/C)C(T/C)(C/T)ACC (T/C)ACC	1589	8.5	+	Phaseolus vulgaris	
HbPAL6	AC-II	TCACCAACCCCC	1591	11	+	Populus tremuloides	
HbPAL6	ARE	AAACCA	843	6	+	Zea mays	cis-acting regulatory element essential for the anaerobic induction
HbPAL6	as-1	TGACG	5	5	+	Arabidopsis thaliana	
HbPAL6	as-1	TGACG	30	5	+	Arabidopsis thaliana	
HbPAL6	as-1	TGACG	76	5	+	Arabidopsis thaliana	
HbPAL6	as-1	TGACG	101	5	-	Arabidopsis thaliana	
HbPAL6	as-1	TGACG	148	5	+	Arabidopsis thaliana	
HbPAL6	as-1	TGACG	207	5	+	Arabidopsis thaliana	
HbPAL6	as-1	TGACG	1566	5	+	Arabidopsis thaliana	
HbPAL6	AT~TATA-box	TATATA	333	6	+	Arabidopsis thaliana	
HbPAL6	AT~TATA-box	TATATAAA	382	8	-	Arabidopsis thaliana	
HbPAL6	AT~TATA-box	TATATA	384	6	+	Arabidopsis thaliana	
HbPAL6	AT~TATA-box	TATATA	402	6	+	Arabidopsis thaliana	
HbPAL6	AT~TATA-box	TATATA	404	6	+	Arabidopsis thaliana	
HbPAL6	AT~TATA-box	TATATAAA	1144	8	-	Arabidopsis thaliana	
HbPAL6	AT~TATA-box	TATATA	1146	6	-	Arabidopsis thaliana	
HbPAL6	AT~TATA-box	TATATA	1148	6	-	Arabidopsis thaliana	
HbPAL6	Box 4	ATTAAT	51	6	+	Petroselinum crispum	part of a conserved DNA module involved in light responsiveness
HbPAL6	Box 4	ATTAAT	391	6	+	Petroselinum crispum	part of a conserved DNA module involved in light responsiveness
HbPAL6	Box 4	ATTAAT	502	6	+	Petroselinum crispum	part of a conserved DNA module involved in light responsiveness
HbPAL6	Box 4	ATTAAT	802	6	+	Petroselinum crispum	part of a conserved DNA module involved in light responsiveness
HbPAL6	Box 4	ATTAAT	1208	6	-	Petroselinum crispum	part of a conserved DNA module involved in light responsiveness
HbPAL6	CAAT-box	CAAT	322	4	+	Nicotiana glutinosa	

HbPAL6	CAAT-box	CAAAT	324	5	-	Pisum sativum	common cis-acting element in promoter and enhancer regions
HbPAL6	CAAT-box	CAAAT	588	5	-	Pisum sativum	common cis-acting element in promoter and enhancer regions
HbPAL6	CAAT-box	CAAAT	604	5	-	Pisum sativum	common cis-acting element in promoter and enhancer regions
HbPAL6	CAAT-box	CAAAT	786	5	+	Pisum sativum	common cis-acting element in promoter and enhancer regions
HbPAL6	CAAT-box	CAAT	822	4	-	Nicotiana glutinosa	
HbPAL6	CAAT-box	CAAT	906	4	+	Nicotiana glutinosa	
HbPAL6	CAAT-box	CAAAT	908	5	-	Pisum sativum	common cis-acting element in promoter and enhancer regions
HbPAL6	CAAT-box	CAAT	918	4	+	Nicotiana glutinosa	
HbPAL6	CAAT-box	CAAT	941	4	+	Nicotiana glutinosa	
HbPAL6	CAAT-box	CAAT	950	4	+	Nicotiana glutinosa	
HbPAL6	CAAT-box	CAAT	984	4	-	Nicotiana glutinosa	
HbPAL6	CAAT-box	CAAAT	1099	5	+	Pisum sativum	common cis-acting element in promoter and enhancer regions
HbPAL6	CAAT-box	CAAT	1118	4	+	Nicotiana glutinosa	
HbPAL6	CAAT-box	CAAT	1212	4	-	Nicotiana glutinosa	
HbPAL6	CAAT-box	CAAAT	1254	5	-	Pisum sativum	common cis-acting element in promoter and enhancer regions
HbPAL6	CAAT-box	CAAT	1268	4	-	Nicotiana glutinosa	
HbPAL6	CAAT-box	CAAAT	1399	5	-	Pisum sativum	common cis-acting element in promoter and enhancer regions
HbPAL6	CAAT-box	CAAAT	1445	5	-	Pisum sativum	common cis-acting element in promoter and enhancer regions
HbPAL6	CAAT-box	CCAAT	1512	5	-	Arabidopsis thaliana	common cis-acting element in promoter and enhancer regions
HbPAL6	CAAT-box	CCAAT	1631	5	+	Arabidopsis thaliana	common cis-acting element in promoter and enhancer regions
HbPAL6	CAAT-box	CAAT	1632	4	+	Nicotiana glutinosa	
HbPAL6	CAAT-box	CCAAT	1674	5	+	Arabidopsis thaliana	common cis-acting element in promoter and enhancer regions
HbPAL6	CAAT-box	CAAT	1675	4	+	Nicotiana glutinosa	
HbPAL6	CAAT-box	CAAT	1717	4	+	Nicotiana glutinosa	
HbPAL6	CAAT-box	CAAAT	1719	5	-	Pisum sativum	common cis-acting element in promoter and enhancer regions
HbPAL6	CAAT-box	CAAT	1827	4	+	Nicotiana glutinosa	

HbPAL6	CAAT-box	CAAT	1991	4	+	Nicotiana glutinosa	
HbPAL6	CCAAT-box	CAACGG	16	6	+	Hordeum vulgare	MYBHv1 binding site
HbPAL6	CCGTCC motif	CCGTCC	1662	6	+	Nicotiana tabacum	
HbPAL6	CCGTCC-box	CCGTCC	1662	6	+	Petroselinum hortense	
HbPAL6	CGTCA-motif	CGTCA	5	5	-	Hordeum vulgare	cis-acting regulatory element involved in the MeJA-responsiveness
HbPAL6	CGTCA-motif	CGTCA	30	5	-	Hordeum vulgare	cis-acting regulatory element involved in the MeJA-responsiveness
HbPAL6	CGTCA-motif	CGTCA	76	5	-	Hordeum vulgare	cis-acting regulatory element involved in the MeJA-responsiveness
HbPAL6	CGTCA-motif	CGTCA	101	5	+	Hordeum vulgare	cis-acting regulatory element involved in the MeJA-responsiveness
HbPAL6	CGTCA-motif	CGTCA	148	5	-	Hordeum vulgare	cis-acting regulatory element involved in the MeJA-responsiveness
HbPAL6	CGTCA-motif	CGTCA	207	5	-	Hordeum vulgare	cis-acting regulatory element involved in the MeJA-responsiveness
HbPAL6	CGTCA-motif	CGTCA	1566	5	-	Hordeum vulgare	cis-acting regulatory element involved in the MeJA-responsiveness
HbPAL6	DRE core	GCCGAC	1796	6	-	Arabidopsis thaliana	dehydration-responsive element
HbPAL6	ERE	ATTTTAAA	375	8	-	Nicotiana glutinos	ethylene-responsive element
HbPAL6	ERE	ATTTTAAA	1011	8	+	Nicotiana glutinos	ethylene-responsive element
HbPAL6	ERE	ATTTTAAA	1342	8	-	Nicotiana glutinos	ethylene-responsive element
HbPAL6	GT1-motif	GGTTAA	1178	6	+	Arabidopsis thaliana	light responsive element
HbPAL6	GT1-motif	GGTTAAT	1415	7	-	Avena sativa	light responsive element
HbPAL6	GT1-motif	GGTTAA	1416	6	-	Arabidopsis thaliana	light responsive element
HbPAL6	MBS	CAACTG	1878	6	-	Arabidopsis thaliana	MYB binding site involved in drought-inducibility
HbPAL6	MRE	AACCTAA	1650	7	+	Petroselinum crispum	MYB binding site involved in light responsiveness
HbPAL6	MYB	CAACCA	775	6	-	Arabidopsis thaliana	MYB binding site
HbPAL6	MYB	CAACCA	1529	6	-	Arabidopsis thaliana	MYB binding site
HbPAL6	MYB	TAACCA	1654	6	+	Arabidopsis thaliana	MYB binding site
HbPAL6	Myb	TAACTG	42	6	+	Arabidopsis thaliana	MYB binding site
HbPAL6	Myb	CAACTG	1878	6	-	Arabidopsis thaliana	MYB binding site
HbPAL6	MYB	CCGTTG	16	6	-	Arabidopsis thaliana	MYB binding site

	recognition site						
HbPAL6	MYB-like sequence	TAACCA	1654	6	+	Arabidopsis thaliana	MYB binding site
HbPAL6	Myc	TCTCTTA	474	7	+	Arabidopsis thaliana	MYC binding site
HbPAL6	MYC	CATTTG	587	6	+	Arabidopsis thaliana	MYC binding site
HbPAL6	O2-site	GATGACATGG	1728	9	-	Zea mays	cis-acting regulatory element involved in zein metabolism regulation
HbPAL6	STRE	AGGGG	1744	5	-	Arabidopsis thaliana	
HbPAL6	STRE	AGGGG	1833	5	-	Arabidopsis thaliana	
HbPAL6	TATA	TATAAAAT	768	8	+	Arabidopsis thaliana	
HbPAL6	TATA	TATAAAAT	1194	8	-	Arabidopsis thaliana	
HbPAL6	TATA-box	TATAAAT	65	7	-	Brassica juncea	core promoter element around -30 of transcription start
HbPAL6	TATA-box	TATAAA	66	6	-	Helianthus annuus	core promoter element around -30 of transcription start
HbPAL6	TATA-box	TATAA	67	5	-	Arabidopsis thaliana	core promoter element around -30 of transcription start
HbPAL6	TATA-box	TATA	68	4	+	Arabidopsis thaliana	core promoter element around -30 of transcription start
HbPAL6	TATA-box	ATTATA	331	6	+	Brassica napus	core promoter element around -30 of transcription start
HbPAL6	TATA-box	TATATAA	332	7	-	Arabidopsis thaliana	core promoter element around -30 of transcription start
HbPAL6	TATA-box	TATATA	333	6	+	Arabidopsis thaliana	core promoter element around -30 of transcription start
HbPAL6	TATA-box	ATATAA	334	6	+	Brassica oleracea	core promoter element around -30 of transcription start
HbPAL6	TATA-box	TATA	335	4	+	Arabidopsis thaliana	core promoter element around -30 of transcription start
HbPAL6	TATA-box	taTATAAAtc	380	9	-	Arabidopsis thaliana	core promoter element around -30 of transcription start
HbPAL6	TATA-box	TATAAAT	381	7	-	Brassica juncea	core promoter element around -30 of transcription start
HbPAL6	TATA-box	TATAAA	382	6	-	Helianthus annuus	core promoter element around -30 of transcription start
HbPAL6	TATA-box	TATATAA	383	7	-	Arabidopsis thaliana	core promoter element around -30 of transcription start
HbPAL6	TATA-box	TATATA	384	6	+	Arabidopsis thaliana	core promoter element around -30 of transcription start
HbPAL6	TATA-box	ATATAA	385	6	+	Brassica oleracea	core promoter element around -30 of transcription start

HbPAL6	TATA-box	TATA	386	4	+	Arabidopsis thaliana	core promoter element around -30 of transcription start
HbPAL6	TATA-box	ATTATA	400	6	+	Brassica napus	core promoter element around -30 of transcription start
HbPAL6	TATA-box	TATATAA	401	7	-	Arabidopsis thaliana	core promoter element around -30 of transcription start
HbPAL6	TATA-box	TATATA	402	6	+	Arabidopsis thaliana	core promoter element around -30 of transcription start
HbPAL6	TATA-box	ATATAT	403	6	+	Brassica napus	core promoter element around -30 of transcription start
HbPAL6	TATA-box	TATATA	404	6	+	Arabidopsis thaliana	core promoter element around -30 of transcription start
HbPAL6	TATA-box	ATATAA	405	6	+	Brassica oleracea	core promoter element around -30 of transcription start
HbPAL6	TATA-box	TATA	406	4	+	Arabidopsis thaliana	core promoter element around -30 of transcription start
HbPAL6	TATA-box	ATATAA	465	6	+	Brassica oleracea	core promoter element around -30 of transcription start
HbPAL6	TATA-box	TATA	466	4	+	Arabidopsis thaliana	core promoter element around -30 of transcription start
HbPAL6	TATA-box	ATTATA	573	6	+	Brassica napus	core promoter element around -30 of transcription start
HbPAL6	TATA-box	TATAA	574	5	-	Arabidopsis thaliana	core promoter element around -30 of transcription start
HbPAL6	TATA-box	TATA	575	4	+	Arabidopsis thaliana	core promoter element around -30 of transcription start
HbPAL6	TATA-box	TATA	740	4	+	Arabidopsis thaliana	core promoter element around -30 of transcription start
HbPAL6	TATA-box	ATATAT	750	6	+	Brassica napus	core promoter element around -30 of transcription start
HbPAL6	TATA-box	TATA	751	4	+	Arabidopsis thaliana	core promoter element around -30 of transcription start
HbPAL6	TATA-box	ATATAA	767	6	+	Brassica oleracea	core promoter element around -30 of transcription start
HbPAL6	TATA-box	TATA	768	4	+	Arabidopsis thaliana	core promoter element around -30 of transcription start
HbPAL6	TATA-box	ATATAA	797	6	+	Brassica oleracea	core promoter element around -30 of transcription start
HbPAL6	TATA-box	TATA	798	4	+	Arabidopsis thaliana	core promoter element around -30 of transcription start
HbPAL6	TATA-box	TATA	882	4	+	Arabidopsis thaliana	core promoter element around -30 of transcription start
HbPAL6	TATA-box	ATTATA	889	6	+	Brassica napus	core promoter element around -30 of transcription start
HbPAL6	TATA-box	TATAA	890	5	-	Arabidopsis thaliana	core promoter element around -30 of transcription start
HbPAL6	TATA-box	TATA	891	4	+	Arabidopsis thaliana	core promoter element around -30 of transcription start
HbPAL6	TATA-box	TATAAAA	969	7	-	Pisum sativum	core promoter element around -30 of transcription start
HbPAL6	TATA-box	TATAAA	970	6	-	Helianthus annuus	core promoter element around -30 of transcription start

HbPAL6	TATA-box	TATAA	971	5	-	Arabidopsis thaliana	core promoter element around -30 of transcription start
HbPAL6	TATA-box	TATA	972	4	+	Arabidopsis thaliana	core promoter element around -30 of transcription start
HbPAL6	TATA-box	ATTATA	1110	6	+	Brassica napus	core promoter element around -30 of transcription start
HbPAL6	TATA-box	TATAA	1111	5	-	Arabidopsis thaliana	core promoter element around -30 of transcription start
HbPAL6	TATA-box	TATA	1112	4	-	Arabidopsis thaliana	core promoter element around -30 of transcription start
HbPAL6	TATA-box	taTATAAAtc	1142	9	-	Arabidopsis thaliana	core promoter element around -30 of transcription start
HbPAL6	TATA-box	TATAAAT	1143	7	-	Brassica juncea	core promoter element around -30 of transcription start
HbPAL6	TATA-box	TATAAA	1144	6	-	Helianthus annuus	core promoter element around -30 of transcription start
HbPAL6	TATA-box	TATATAA	1145	7	-	Arabidopsis thaliana	core promoter element around -30 of transcription start
HbPAL6	TATA-box	TATATA	1146	6	-	Arabidopsis thaliana	core promoter element around -30 of transcription start
HbPAL6	TATA-box	ATATAT	1147	6	-	Brassica napus	core promoter element around -30 of transcription start
HbPAL6	TATA-box	TATATA	1148	6	-	Arabidopsis thaliana	core promoter element around -30 of transcription start
HbPAL6	TATA-box	ATATAA	1149	6	+	Brassica oleracea	core promoter element around -30 of transcription start
HbPAL6	TATA-box	TATA	1150	4	-	Arabidopsis thaliana	core promoter element around -30 of transcription start
HbPAL6	TATA-box	TATAAATA	1166	8	-	Daucus carota	core promoter element around -30 of transcription start
HbPAL6	TATA-box	TATAAAT	1167	7	-	Brassica juncea	core promoter element around -30 of transcription start
HbPAL6	TATA-box	TATAAA	1168	6	-	Helianthus annuus	core promoter element around -30 of transcription start
HbPAL6	TATA-box	TATAA	1169	5	-	Arabidopsis thaliana	core promoter element around -30 of transcription start
HbPAL6	TATA-box	TATA	1170	4	-	Arabidopsis thaliana	core promoter element around -30 of transcription start
HbPAL6	TATA-box	TATAAAA	1195	7	-	Pisum sativum	core promoter element around -30 of transcription start
HbPAL6	TATA-box	TATAAA	1196	6	-	Helianthus annuus	core promoter element around -30 of transcription start
HbPAL6	TATA-box	TATAA	1197	5	-	Arabidopsis thaliana	core promoter element around -30 of transcription start
HbPAL6	TATA-box	TATA	1198	4	-	Arabidopsis thaliana	core promoter element around -30 of transcription start
HbPAL6	TATA-box	TATA	1285	4	-	Arabidopsis thaliana	core promoter element around -30 of transcription start
HbPAL6	TATA-box	ATTATA	1348	6	+	Brassica napus	core promoter element around -30 of transcription start
HbPAL6	TATA-box	TATAA	1349	5	-	Arabidopsis thaliana	core promoter element around -30 of transcription start

HbPAL6	TATA-box	TATA	1350	4	-	Arabidopsis thaliana	core promoter element around -30 of transcription start
HbPAL6	TATA-box	ATATAT	1682	6	-	Brassica napus	core promoter element around -30 of transcription start
HbPAL6	TATA-box	TATA	1683	4	-	Arabidopsis thaliana	core promoter element around -30 of transcription start
HbPAL6	TATA-box	taTATAAAtc	1756	9	+	Arabidopsis thaliana	core promoter element around -30 of transcription start
HbPAL6	TATA-box	ATATAA	1757	6	+	Brassica oleracea	core promoter element around -30 of transcription start
HbPAL6	TATA-box	TATA	1758	4	-	Arabidopsis thaliana	core promoter element around -30 of transcription start
HbPAL6	TATA-box	TATTTAAA	1860	8	+	Arabidopsis thaliana	core promoter element around -30 of transcription start
HbPAL6	TATA-box	TATA	1925	4	-	Arabidopsis thaliana	core promoter element around -30 of transcription start
HbPAL6	TCCC-motif	TCTCCCT	271	7	+	Spinacia oleracea	part of a light responsive element
HbPAL6	TC-rich repeats	ATTCTCTAAC	628	9	-	Nicotiana tabacum	cis-acting element involved in defense and stress responsiveness
HbPAL6	TCT-motif	TCTTAC	700	6	-	Arabidopsis thaliana	part of a light responsive element
HbPAL6	TCT-motif	TCTTAC	871	6	-	Arabidopsis thaliana	part of a light responsive element
HbPAL6	TCT-motif	TCTTAC	1739	6	+	Arabidopsis thaliana	part of a light responsive element
HbPAL6	TGACG-motif	TGACG	5	5	+	Hordeum vulgare	cis-acting regulatory element involved in the MeJA-responsiveness
HbPAL6	TGACG-motif	TGACG	30	5	+	Hordeum vulgare	cis-acting regulatory element involved in the MeJA-responsiveness
HbPAL6	TGACG-motif	TGACG	76	5	+	Hordeum vulgare	cis-acting regulatory element involved in the MeJA-responsiveness
HbPAL6	TGACG-motif	TGACG	101	5	-	Hordeum vulgare	cis-acting regulatory element involved in the MeJA-responsiveness
HbPAL6	TGACG-motif	TGACG	148	5	+	Hordeum vulgare	cis-acting regulatory element involved in the MeJA-responsiveness
HbPAL6	TGACG-motif	TGACG	207	5	+	Hordeum vulgare	cis-acting regulatory element involved in the MeJA-responsiveness
HbPAL6	TGACG-motif	TGACG	1566	5	+	Hordeum vulgare	cis-acting regulatory element involved in the MeJA-responsiveness
HbPAL6	TGA-element	AACGAC	190	6	-	Brassica oleracea	auxin-responsive element
HbPAL6	WRE3	CCACCT	1974	6	+	Pisum sativum	wound-responsive element
HbPAL6	WUN-motif	TTATTACAT	515	9	+	Nicotiana glutinosa	wound-responsive element
HbPAL6	WUN-motif	AAATTTCCT	1912	9	+	Brassica oleracea	wound-responsive element
HbPAL7	ABRE	ACGTG	978	5	+	Arabidopsis thaliana	cis-acting element involved in the abscisic acid responsiveness
HbPAL7	ABRE3a	TACGTG	977	6	+	Zea mays	cis-acting element involved in the abscisic acid responsiveness

HbPAL7	ABRE4	CACGTA	977	6	-	Zea mays	cis-acting element involved in the abscisic acid responsiveness
HbPAL7	ARE	AAACCA	157	6	-	Zea mays	cis-acting regulatory element essential for the anaerobic induction
HbPAL7	ARE	AAACCA	216	6	-	Zea mays	cis-acting regulatory element essential for the anaerobic induction
HbPAL7	ARE	AAACCA	1729	6	+	Zea mays	cis-acting regulatory element essential for the anaerobic induction
HbPAL7	as-1	TGACG	248	5	+	Arabidopsis thaliana	
HbPAL7	as-1	TGACG	398	5	+	Arabidopsis thaliana	
HbPAL7	as-1	TGACG	1059	5	+	Arabidopsis thaliana	
HbPAL7	AT~TATA-box	TATATA	734	6	+	Arabidopsis thaliana	
HbPAL7	AT~TATA-box	TATATA	736	6	+	Arabidopsis thaliana	
HbPAL7	AT~TATA-box	TATATAAA	905	8	-	Arabidopsis thaliana	
HbPAL7	AT~TATA-box	TATATA	907	6	+	Arabidopsis thaliana	
HbPAL7	AT~TATA-box	TATATA	1018	6	-	Arabidopsis thaliana	
HbPAL7	AT~TATA-box	TATATA	1020	6	-	Arabidopsis thaliana	
HbPAL7	AT~TATA-box	TATATA	1022	6	-	Arabidopsis thaliana	
HbPAL7	AT~TATA-box	TATATAAA	1256	8	-	Arabidopsis thaliana	
HbPAL7	AT~TATA-box	TATATA	1258	6	-	Arabidopsis thaliana	
HbPAL7	AT~TATA-box	TATATAAA	1295	8	-	Arabidopsis thaliana	
HbPAL7	AT~TATA-box	TATATA	1297	6	-	Arabidopsis thaliana	
HbPAL7	AT~TATA-box	TATATA	1350	6	-	Arabidopsis thaliana	
HbPAL7	Box 4	ATTAAT	833	6	+	Petroselinum crispum	part of a conserved DNA module involved in light responsiveness
HbPAL7	Box 4	ATTAAT	1086	6	-	Petroselinum crispum	part of a conserved DNA module involved in light responsiveness
HbPAL7	box S	AGCCACC	1561	7	-	Arabidopsis thaliana	
HbPAL7	CAAT-box	CAAT	103	4	-	Nicotiana glutinosa	
HbPAL7	CAAT-box	CAAT	109	4	-	Nicotiana glutinosa	
HbPAL7	CAAT-box	CAAT	135	4	-	Nicotiana glutinosa	
HbPAL7	CAAT-box	CAAT	265	4	-	Nicotiana glutinosa	

HbPAL7	CAAT-box	CAAT	282	4	+	Nicotiana glutinosa	
HbPAL7	CAAT-box	CAAAT	301	5	-	Pisum sativum	common cis-acting element in promoter and enhancer regions
HbPAL7	CAAT-box	CAAT	351	4	-	Nicotiana glutinosa	
HbPAL7	CAAT-box	CAAT	396	4	-	Nicotiana glutinosa	
HbPAL7	CAAT-box	CCAAT	413	5	+	Arabidopsis thaliana	common cis-acting element in promoter and enhancer regions
HbPAL7	CAAT-box	CAAT	414	4	+	Nicotiana glutinosa	
HbPAL7	CAAT-box	CAAT	471	4	+	Nicotiana glutinosa	
HbPAL7	CAAT-box	CAAT	481	4	+	Nicotiana glutinosa	
HbPAL7	CAAT-box	CAAT	612	4	-	Nicotiana glutinosa	
HbPAL7	CAAT-box	CAAT	619	4	+	Nicotiana glutinosa	
HbPAL7	CAAT-box	CAAT	621	4	-	Nicotiana glutinosa	
HbPAL7	CAAT-box	CAAT	704	4	-	Nicotiana glutinosa	
HbPAL7	CAAT-box	CAAT	857	4	-	Nicotiana glutinosa	
HbPAL7	CAAT-box	CAAT	898	4	+	Nicotiana glutinosa	
HbPAL7	CAAT-box	CAAT	925	4	+	Nicotiana glutinosa	
HbPAL7	CAAT-box	CAAT	997	4	+	Nicotiana glutinosa	
HbPAL7	CAAT-box	CAAT	1003	4	+	Nicotiana glutinosa	
HbPAL7	CAAT-box	CAAAT	1080	5	+	Pisum sativum	common cis-acting element in promoter and enhancer regions
HbPAL7	CAAT-box	CAAT	1173	4	+	Nicotiana glutinosa	
HbPAL7	CAAT-box	CAAT	1188	4	-	Nicotiana glutinosa	
HbPAL7	CAAT-box	CAAT	1216	4	-	Nicotiana glutinosa	
HbPAL7	CAAT-box	CAAT	1236	4	+	Nicotiana glutinosa	
HbPAL7	CAAT-box	CAAAT	1306	5	-	Pisum sativum	common cis-acting element in promoter and enhancer regions
HbPAL7	CAAT-box	CAAAT	1375	5	+	Pisum sativum	common cis-acting element in promoter and enhancer regions
HbPAL7	CAAT-box	CAAT	1407	4	-	Nicotiana glutinosa	
HbPAL7	CAAT-box	CAAT	1416	4	+	Nicotiana glutinosa	

HbPAL7	CAAT-box	CCAAT	1445	5	-	Arabidopsis thaliana	common cis-acting element in promoter and enhancer regions
HbPAL7	CAAT-box	CAAAT	1454	5	+	Pisum sativum	common cis-acting element in promoter and enhancer regions
HbPAL7	CAAT-box	CAAT	1490	4	-	Nicotiana glutinosa	
HbPAL7	CAAT-box	CAAT	1496	4	-	Nicotiana glutinosa	
HbPAL7	CAAT-box	CAAAT	1503	5	+	Pisum sativum	common cis-acting element in promoter and enhancer regions
HbPAL7	CAAT-box	CAAT	1535	4	+	Nicotiana glutinosa	
HbPAL7	CAAT-box	CCAAT	1540	5	+	Arabidopsis thaliana	common cis-acting element in promoter and enhancer regions
HbPAL7	CAAT-box	CAAT	1541	4	+	Nicotiana glutinosa	
HbPAL7	CAAT-box	CAAT	1590	4	+	Nicotiana glutinosa	
HbPAL7	CAAT-box	CAAAT	1660	5	-	Pisum sativum	common cis-acting element in promoter and enhancer regions
HbPAL7	CAAT-box	CAAT	1668	4	+	Nicotiana glutinosa	
HbPAL7	CAAT-box	CAAAT	1752	5	+	Pisum sativum	common cis-acting element in promoter and enhancer regions
HbPAL7	CAAT-box	CAAAT	1775	5	+	Pisum sativum	common cis-acting element in promoter and enhancer regions
HbPAL7	CAAT-box	CAAT	1914	4	+	Nicotiana glutinosa	
HbPAL7	CAAT-box	CAAT	1920	4	+	Nicotiana glutinosa	
HbPAL7	CAAT-box	CAAT	1938	4	+	Nicotiana glutinosa	
HbPAL7	CAAT-box	CCAAT	1952	5	+	Arabidopsis thaliana	common cis-acting element in promoter and enhancer regions
HbPAL7	CAAT-box	CAAT	1953	4	+	Nicotiana glutinosa	
HbPAL7	CAAT-box	CAAT	1962	4	+	Nicotiana glutinosa	
HbPAL7	CGTCA-motif	CGTCA	248	5	-	Hordeum vulgare	cis-acting regulatory element involved in the MeJA-responsiveness
HbPAL7	CGTCA-motif	CGTCA	398	5	-	Hordeum vulgare	cis-acting regulatory element involved in the MeJA-responsiveness
HbPAL7	CGTCA-motif	CGTCA	1059	5	-	Hordeum vulgare	cis-acting regulatory element involved in the MeJA-responsiveness
HbPAL7	circadian	CAAAGATATC	175	9	+	Lycopersicon esculentum	cis-acting regulatory element involved in circadian control
HbPAL7	DRE core	GCCGAC	1370	6	-	Arabidopsis thaliana	dehydration-responsive element
HbPAL7	ERE	ATTTTAAA	1045	8	-	Nicotiana glutinos	ethylene-responsive element

HbPAL7	ERE	ATTTTAAA	1280	8	+	Nicotiana glutinos	ethylene-responsive element
HbPAL7	ERE	ATTTTAAA	1282	8	-	Nicotiana glutinos	ethylene-responsive element
HbPAL7	ERE	ATTTCATA	1596	8	-	Nicotiana glutinos	ethylene-responsive element
HbPAL7	GARE-motif	TCTGTTG	330	7	-	Brassica oleracea	gibberellin-responsive element
HbPAL7	GATA-motif	GATAGGA	649	7	-	Arabidopsis thaliana	part of a light responsive element
HbPAL7	G-box	TACGTG	977	6	+	Arabidopsis thaliana	cis-acting regulatory element involved in light responsiveness
HbPAL7	GCN4_motif	TGAGTCA	614	7	+	Oryza sativa	cis-regulatory element involved in endosperm expression
HbPAL7	LAMP-element	CTTTATCA	1784	8	-	Pisum sativum	part of a light responsive element
HbPAL7	LTR	CCGAAA	1052	6	-	Hordeum vulgare	cis-acting element involved in low-temperature responsiveness
HbPAL7	MYB	TAACCA	17	6	+	Arabidopsis thaliana	MYB binding site
HbPAL7	MYB	CAACAG	330	6	+	Arabidopsis thaliana	MYB binding site
HbPAL7	MYB	CAACCA	1719	6	+	Arabidopsis thaliana	MYB binding site
HbPAL7	MYB	CAACCA	1723	6	+	Arabidopsis thaliana	MYB binding site
HbPAL7	Myb	TAACTG	728	6	+	Arabidopsis thaliana	MYB binding site
HbPAL7	Myb-binding site	CAACAG	330	6	+	Nicotiana tabacum	MYB binding site
HbPAL7	MYB-like sequence	TAACCA	17	6	+	Arabidopsis thaliana	MYB binding site
HbPAL7	MYC	CAATTG	619	6	+	Arabidopsis thaliana	MYC binding site
HbPAL7	MYC	CATTTG	1659	6	+	Arabidopsis thaliana	MYC binding site
HbPAL7	MYC	CATGTG	1733	6	+	Arabidopsis thaliana	MYC binding site
HbPAL7	Myc	TCTCTTA	960	7	+	Arabidopsis thaliana	MYC binding site
HbPAL7	O2-site	GATGATGTGG	91	9	+	Zea mays	cis-acting regulatory element involved in zein metabolism regulation
HbPAL7	TATA	TATAAAAT	903	8	-	Arabidopsis thaliana	
HbPAL7	TATA	TATAAAAT	946	8	+	Arabidopsis thaliana	

HbPAL7	TATA-box	ATATAT	131	6	+	Brassica napus	core promoter element around -30 of transcription start
HbPAL7	TATA-box	TATA	132	4	+	Arabidopsis thaliana	core promoter element around -30 of transcription start
HbPAL7	TATA-box	TATA	298	4	+	Arabidopsis thaliana	core promoter element around -30 of transcription start
HbPAL7	TATA-box	TATA	695	4	+	Arabidopsis thaliana	core promoter element around -30 of transcription start
HbPAL7	TATA-box	TATACA	732	6	-	Helianthus annuus	core promoter element around -30 of transcription start
HbPAL7	TATA-box	TATATA	734	6	+	Arabidopsis thaliana	core promoter element around -30 of transcription start
HbPAL7	TATA-box	ATATAT	735	6	+	Brassica napus	core promoter element around -30 of transcription start
HbPAL7	TATA-box	TATATA	736	6	+	Arabidopsis thaliana	core promoter element around -30 of transcription start
HbPAL7	TATA-box	ATATAT	737	6	+	Brassica napus	core promoter element around -30 of transcription start
HbPAL7	TATA-box	TATA	738	4	+	Arabidopsis thaliana	core promoter element around -30 of transcription start
HbPAL7	TATA-box	ATTATA	814	6	+	Brassica napus	core promoter element around -30 of transcription start
HbPAL7	TATA-box	TATAA	815	5	-	Arabidopsis thaliana	core promoter element around -30 of transcription start
HbPAL7	TATA-box	TATA	816	4	+	Arabidopsis thaliana	core promoter element around -30 of transcription start
HbPAL7	TATA-box	ATTATA	843	6	+	Brassica napus	core promoter element around -30 of transcription start
HbPAL7	TATA-box	TATAA	844	5	-	Arabidopsis thaliana	core promoter element around -30 of transcription start
HbPAL7	TATA-box	TATA	845	4	+	Arabidopsis thaliana	core promoter element around -30 of transcription start
HbPAL7	TATA-box	TATAAAA	904	7	-	Pisum sativum	core promoter element around -30 of transcription start
HbPAL7	TATA-box	TATAAA	905	6	-	Helianthus annuus	core promoter element around -30 of transcription start
HbPAL7	TATA-box	TATATAA	906	7	-	Arabidopsis thaliana	core promoter element around -30 of transcription start
HbPAL7	TATA-box	TATATA	907	6	+	Arabidopsis thaliana	core promoter element around -30 of transcription start
HbPAL7	TATA-box	ATATAT	908	6	+	Brassica napus	core promoter element around -30 of transcription start
HbPAL7	TATA-box	TATA	909	4	+	Arabidopsis thaliana	core promoter element around -30 of transcription start
HbPAL7	TATA-box	TATAA	945	5	-	Arabidopsis thaliana	core promoter element around -30 of transcription start
HbPAL7	TATA-box	TATA	946	4	+	Arabidopsis thaliana	core promoter element around -30 of transcription start
HbPAL7	TATA-box	TATA	975	4	+	Arabidopsis thaliana	core promoter element around -30 of transcription start
HbPAL7	TATA-box	TATACA	1016	6	-	Helianthus annuus	core promoter element around -30 of transcription start

HbPAL7	TATA-box	TATATA	1018	6	-	Arabidopsis thaliana	core promoter element around -30 of transcription start
HbPAL7	TATA-box	ATATAT	1019	6	-	Brassica napus	core promoter element around -30 of transcription start
HbPAL7	TATA-box	TATATA	1020	6	-	Arabidopsis thaliana	core promoter element around -30 of transcription start
HbPAL7	TATA-box	ATATAT	1021	6	-	Brassica napus	core promoter element around -30 of transcription start
HbPAL7	TATA-box	TATATA	1022	6	-	Arabidopsis thaliana	core promoter element around -30 of transcription start
HbPAL7	TATA-box	ATATAA	1023	6	+	Brassica oleracea	core promoter element around -30 of transcription start
HbPAL7	TATA-box	TATA	1024	4	-	Arabidopsis thaliana	core promoter element around -30 of transcription start
HbPAL7	TATA-box	TATAAA	1034	6	-	Helianthus annuus	core promoter element around -30 of transcription start
HbPAL7	TATA-box	TATAA	1035	5	-	Arabidopsis thaliana	core promoter element around -30 of transcription start
HbPAL7	TATA-box	TATA	1036	4	-	Arabidopsis thaliana	core promoter element around -30 of transcription start
HbPAL7	TATA-box	TATA	1150	4	-	Arabidopsis thaliana	core promoter element around -30 of transcription start
HbPAL7	TATA-box	ATATAA	1198	6	+	Brassica oleracea	core promoter element around -30 of transcription start
HbPAL7	TATA-box	TATA	1199	4	-	Arabidopsis thaliana	core promoter element around -30 of transcription start
HbPAL7	TATA-box	ATATAT	1212	6	-	Brassica napus	core promoter element around -30 of transcription start
HbPAL7	TATA-box	TATA	1213	4	-	Arabidopsis thaliana	core promoter element around -30 of transcription start
HbPAL7	TATA-box	TATACA	1218	6	-	Helianthus annuus	core promoter element around -30 of transcription start
HbPAL7	TATA-box	TATA	1220	4	-	Arabidopsis thaliana	core promoter element around -30 of transcription start
HbPAL7	TATA-box	TATA	1243	4	-	Arabidopsis thaliana	core promoter element around -30 of transcription start
HbPAL7	TATA-box	taTATAAAg	1255	9	-	Lycopersicon esculentum	core promoter element around -30 of transcription start
HbPAL7	TATA-box	TATAAA	1256	6	-	Helianthus annuus	core promoter element around -30 of transcription start
HbPAL7	TATA-box	TATATAA	1257	7	-	Arabidopsis thaliana	core promoter element around -30 of transcription start
HbPAL7	TATA-box	TATATA	1258	6	-	Arabidopsis thaliana	core promoter element around -30 of transcription start
HbPAL7	TATA-box	ATATAA	1259	6	+	Brassica oleracea	core promoter element around -30 of transcription start
HbPAL7	TATA-box	TATA	1260	4	-	Arabidopsis thaliana	core promoter element around -30 of transcription start
HbPAL7	TATA-box	TATA	1272	4	-	Arabidopsis thaliana	core promoter element around -30 of transcription start

HbPAL7	TATA-box	taTATAAAtc	1293	9	-	Arabidopsis thaliana	core promoter element around -30 of transcription start
HbPAL7	TATA-box	TATAAAT	1294	7	-	Brassica juncea	core promoter element around -30 of transcription start
HbPAL7	TATA-box	TATAAA	1295	6	-	Helianthus annuus	core promoter element around -30 of transcription start
HbPAL7	TATA-box	TATATAA	1296	7	-	Arabidopsis thaliana	core promoter element around -30 of transcription start
HbPAL7	TATA-box	TATATA	1297	6	-	Arabidopsis thaliana	core promoter element around -30 of transcription start
HbPAL7	TATA-box	ATATAA	1298	6	+	Brassica oleracea	core promoter element around -30 of transcription start
HbPAL7	TATA-box	TATA	1299	4	-	Arabidopsis thaliana	core promoter element around -30 of transcription start
HbPAL7	TATA-box	ATATAT	1349	6	-	Brassica napus	core promoter element around -30 of transcription start
HbPAL7	TATA-box	TATATA	1350	6	-	Arabidopsis thaliana	core promoter element around -30 of transcription start
HbPAL7	TATA-box	ATATAT	1351	6	-	Brassica napus	core promoter element around -30 of transcription start
HbPAL7	TATA-box	TATA	1352	4	-	Arabidopsis thaliana	core promoter element around -30 of transcription start
HbPAL7	TATA-box	ATATAA	1471	6	+	Brassica oleracea	core promoter element around -30 of transcription start
HbPAL7	TATA-box	TATA	1472	4	-	Arabidopsis thaliana	core promoter element around -30 of transcription start
HbPAL7	TATA-box	TATA	1615	4	-	Arabidopsis thaliana	core promoter element around -30 of transcription start
HbPAL7	TATA-box	ATTATA	1699	6	+	Brassica napus	core promoter element around -30 of transcription start
HbPAL7	TATA-box	TATAA	1700	5	-	Arabidopsis thaliana	core promoter element around -30 of transcription start
HbPAL7	TATA-box	TATA	1701	4	-	Arabidopsis thaliana	core promoter element around -30 of transcription start
HbPAL7	TATA-box	ATATAA	1792	6	+	Brassica oleracea	core promoter element around -30 of transcription start
HbPAL7	TATA-box	TATA	1793	4	-	Arabidopsis thaliana	core promoter element around -30 of transcription start
HbPAL7	TATA-box	ATTATA	1810	6	+	Brassica napus	core promoter element around -30 of transcription start
HbPAL7	TATA-box	TATAA	1811	5	-	Arabidopsis thaliana	core promoter element around -30 of transcription start
HbPAL7	TATA-box	TATA	1812	4	-	Arabidopsis thaliana	core promoter element around -30 of transcription start
HbPAL7	TATA-box	TATA	1883	4	-	Arabidopsis thaliana	core promoter element around -30 of transcription start
HbPAL7	TCA	TCATCTTCAT	1335	10	+	Pisum sativum	
HbPAL7	TGACG-motif	TGACG	248	5	+	Hordeum vulgare	cis-acting regulatory element involved in the MeJA-responsiveness
HbPAL7	TGACG-motif	TGACG	398	5	+	Hordeum vulgare	cis-acting regulatory element involved in the MeJA-responsiveness

HbPAL7	TGACG-motif	TGACG	1059	5	+	Hordeum vulgare	cis-acting regulatory element involved in the MeJA-responsiveness
HbPAL7	W box	TTGACC	1587	6	-	Arabidopsis thaliana	WRKY binding site
HbPAL7	W box	TTGACC	1665	6	-	Arabidopsis thaliana	WRKY binding site
HbPAL7	WUN-motif	AAATTTCCT	423	9	-	Brassica oleracea	wound-responsive element
HbPAL8	AAGAA-motif	gGTAAAGAAA	1423	9	+	Avena sativa	
HbPAL8	AAGAA-motif	gGTAAAGAAA	1668	9	-	Avena sativa	
HbPAL8	ARE	AAACCA	48	6	+	Zea mays	cis-acting regulatory element essential for the anaerobic induction
HbPAL8	ARE	AAACCA	209	6	-	Zea mays	cis-acting regulatory element essential for the anaerobic induction
HbPAL8	AT~TATA-box	TATATA	1204	6	-	Arabidopsis thaliana	
HbPAL8	AT1-motif	AATTATTTTTTATT	1476	13	+	Solanum tuberosum	part of a light responsive module
HbPAL8	AT1-motif	AATTATTTTTTATT	1596	14	-	Solanum tuberosum	part of a light responsive module
HbPAL8	Box 4	ATTAAT	1096	6	-	Petroselinum crispum	part of a conserved DNA module involved in light responsiveness
HbPAL8	Box 4	ATTAAT	1100	6	-	Petroselinum crispum	part of a conserved DNA module involved in light responsiveness
HbPAL8	Box 4	ATTAAT	1581	6	-	Petroselinum crispum	part of a conserved DNA module involved in light responsiveness
HbPAL8	CAAT-box	CAAT	5	4	+	Nicotiana glutinosa	
HbPAL8	CAAT-box	CAAAT	34	5	-	Pisum sativum	common cis-acting element in promoter and enhancer regions
HbPAL8	CAAT-box	CAAT	71	4	+	Nicotiana glutinosa	
HbPAL8	CAAT-box	CAAT	83	4	+	Nicotiana glutinosa	
HbPAL8	CAAT-box	CAAAT	88	5	-	Pisum sativum	common cis-acting element in promoter and enhancer regions
HbPAL8	CAAT-box	CAAT	119	4	+	Nicotiana glutinosa	
HbPAL8	CAAT-box	CAAT	126	4	-	Nicotiana glutinosa	
HbPAL8	CAAT-box	CAAT	196	4	-	Nicotiana glutinosa	
HbPAL8	CAAT-box	CAAAT	215	5	+	Pisum sativum	common cis-acting element in promoter and enhancer regions
HbPAL8	CAAT-box	CAAAT	338	5	+	Pisum sativum	common cis-acting element in promoter and enhancer regions
HbPAL8	CAAT-box	CAAAT	459	5	-	Pisum sativum	common cis-acting element in promoter and enhancer regions
HbPAL8	CAAT-box	CAAT	537	4	+	Nicotiana glutinosa	

HbPAL8	CAAT-box	CAAAT	542	5	+	Pisum sativum	common cis-acting element in promoter and enhancer regions
HbPAL8	CAAT-box	CAAT	694	4	-	Nicotiana glutinosa	
HbPAL8	CAAT-box	CAAAT	716	5	-	Pisum sativum	common cis-acting element in promoter and enhancer regions
HbPAL8	CAAT-box	CAAT	775	4	+	Nicotiana glutinosa	
HbPAL8	CAAT-box	CCAAT	822	5	+	Arabidopsis thaliana	common cis-acting element in promoter and enhancer regions
HbPAL8	CAAT-box	CAAT	823	4	+	Nicotiana glutinosa	
HbPAL8	CAAT-box	CAAT	998	4	-	Nicotiana glutinosa	
HbPAL8	CAAT-box	CAAAT	1009	5	-	Pisum sativum	common cis-acting element in promoter and enhancer regions
HbPAL8	CAAT-box	CAAT	1016	4	-	Nicotiana glutinosa	
HbPAL8	CAAT-box	CAAAT	1111	5	+	Pisum sativum	common cis-acting element in promoter and enhancer regions
HbPAL8	CAAT-box	CAAT	1135	4	-	Nicotiana glutinosa	
HbPAL8	CAAT-box	CAAT	1299	4	-	Nicotiana glutinosa	
HbPAL8	CAAT-box	CAAAT	1337	5	+	Pisum sativum	common cis-acting element in promoter and enhancer regions
HbPAL8	CAAT-box	CAAAT	1340	5	-	Pisum sativum	common cis-acting element in promoter and enhancer regions
HbPAL8	CAAT-box	CAAT	1696	4	+	Nicotiana glutinosa	
HbPAL8	CAAT-box	CAAT	1844	4	-	Nicotiana glutinosa	
HbPAL8	CAAT-box	CCAAT	1901	5	+	Arabidopsis thaliana	common cis-acting element in promoter and enhancer regions
HbPAL8	CAAT-box	CAAT	1902	4	+	Nicotiana glutinosa	
HbPAL8	CAAT-box	CAAAT	1954	5	+	Pisum sativum	common cis-acting element in promoter and enhancer regions
HbPAL8	CAAT-box	CAAT	1979	4	+	Nicotiana glutinosa	
HbPAL8	ERE	ATTTTAAA	1569	8	-	Nicotiana glutinos	ethylene-responsive element
HbPAL8	GATA-motif	GATAGGA	802	7	-	Arabidopsis thaliana	part of a light responsive element
HbPAL8	G-box	CACGAC	571	6	+	Zea mays	cis-acting regulatory element involved in light responsiveness
HbPAL8	GT1-motif	GGTTAA	134	6	+	Arabidopsis thaliana	light responsive element
HbPAL8	GT1-motif	GTGTGTGAA	497	9	+	Solanum tuberosum	light responsive element
HbPAL8	MRE	AACCTAA	648	7	-	Petroselinum crispum	MYB binding site involved in light responsiveness

HbPAL8	Myb	TAACTG	840	6	+	Arabidopsis thaliana	MYB binding site
HbPAL8	Myb	TAACTG	893	6	-	Arabidopsis thaliana	MYB binding site
HbPAL8	Myb	TAACTG	980	6	-	Arabidopsis thaliana	MYB binding site
HbPAL8	MYB	CAACCA	103	6	-	Arabidopsis thaliana	MYB binding site
HbPAL8	MYB	TAACCA	191	6	+	Arabidopsis thaliana	MYB binding site
HbPAL8	MYB	CAACCA	379	6	-	Arabidopsis thaliana	MYB binding site
HbPAL8	MYB-like sequence	TAACCA	191	6	+	Arabidopsis thaliana	MYB binding site
HbPAL8	MYC	CATTTG	33	6	+	Arabidopsis thaliana	MYC binding site
HbPAL8	MYC	CATGTG	424	6	+	Arabidopsis thaliana	MYC binding site
HbPAL8	MYC	CATTTG	542	6	-	Arabidopsis thaliana	MYC binding site
HbPAL8	MYC	CATTTG	715	6	+	Arabidopsis thaliana	MYC binding site
HbPAL8	O2-site	GATGACATGG	627	9	-	Zea mays	cis-acting regulatory element involved in zein metabolism regulation
HbPAL8	STRE	AGGGG	349	5	+	Arabidopsis thaliana	
HbPAL8	TATA	TATAAAAT	1607	8	-	Arabidopsis thaliana	
HbPAL8	TATA-box	ATTATA	231	6	+	Brassica napus	core promoter element around -30 of transcription start
HbPAL8	TATA-box	TATAA	232	5	-	Arabidopsis thaliana	core promoter element around -30 of transcription start
HbPAL8	TATA-box	TATA	233	4	+	Arabidopsis thaliana	core promoter element around -30 of transcription start
HbPAL8	TATA-box	ccTATAAAaa	261	9	-	Arabidopsis thaliana	core promoter element around -30 of transcription start
HbPAL8	TATA-box	TATAAAA	262	7	-	Pisum sativum	core promoter element around -30 of transcription start
HbPAL8	TATA-box	TATAAA	263	6	-	Helianthus annuus	core promoter element around -30 of transcription start
HbPAL8	TATA-box	TATAA	264	5	-	Arabidopsis thaliana	core promoter element around -30 of transcription start
HbPAL8	TATA-box	TATA	265	4	+	Arabidopsis thaliana	core promoter element around -30 of transcription start
HbPAL8	TATA-box	TATA	681	4	+	Arabidopsis thaliana	core promoter element around -30 of transcription start
HbPAL8	TATA-box	TATA	768	4	+	Arabidopsis thaliana	core promoter element around -30 of transcription start

HbPAL8	TATA-box	TATAAAA	946	7	-	Pisum sativum	core promoter element around -30 of transcription start
HbPAL8	TATA-box	TATAAA	947	6	-	Helianthus annuus	core promoter element around -30 of transcription start
HbPAL8	TATA-box	TATAA	948	5	-	Arabidopsis thaliana	core promoter element around -30 of transcription start
HbPAL8	TATA-box	TATA	949	4	+	Arabidopsis thaliana	core promoter element around -30 of transcription start
HbPAL8	TATA-box	ATATAT	1066	6	-	Brassica napus	core promoter element around -30 of transcription start
HbPAL8	TATA-box	TATA	1067	4	-	Arabidopsis thaliana	core promoter element around -30 of transcription start
HbPAL8	TATA-box	ATATAA	1077	6	+	Brassica oleracea	core promoter element around -30 of transcription start
HbPAL8	TATA-box	TATA	1078	4	-	Arabidopsis thaliana	core promoter element around -30 of transcription start
HbPAL8	TATA-box	TATA	1156	4	-	Arabidopsis thaliana	core promoter element around -30 of transcription start
HbPAL8	TATA-box	ATTATA	1184	6	+	Brassica napus	core promoter element around -30 of transcription start
HbPAL8	TATA-box	TATAA	1185	5	-	Arabidopsis thaliana	core promoter element around -30 of transcription start
HbPAL8	TATA-box	TATA	1186	4	-	Arabidopsis thaliana	core promoter element around -30 of transcription start
HbPAL8	TATA-box	ATTATA	1202	6	+	Brassica napus	core promoter element around -30 of transcription start
HbPAL8	TATA-box	TATATAA	1203	7	-	Arabidopsis thaliana	core promoter element around -30 of transcription start
HbPAL8	TATA-box	TATATA	1204	6	-	Arabidopsis thaliana	core promoter element around -30 of transcription start
HbPAL8	TATA-box	ATATAT	1205	6	-	Brassica napus	core promoter element around -30 of transcription start
HbPAL8	TATA-box	TATA	1206	4	-	Arabidopsis thaliana	core promoter element around -30 of transcription start
HbPAL8	TATA-box	TATA	1269	4	-	Arabidopsis thaliana	core promoter element around -30 of transcription start
HbPAL8	TATA-box	TAAAGATT	1284	8	+	Arabidopsis thaliana	core promoter element around -30 of transcription start
HbPAL8	TATA-box	TATA	1310	4	-	Arabidopsis thaliana	core promoter element around -30 of transcription start
HbPAL8	TATA-box	ATATAA	1469	6	+	Brassica oleracea	core promoter element around -30 of transcription start
HbPAL8	TATA-box	TATA	1470	4	-	Arabidopsis thaliana	core promoter element around -30 of transcription start
HbPAL8	TATA-box	TATTTAAA	1535	8	-	Arabidopsis thaliana	core promoter element around -30 of transcription start
HbPAL8	TATA-box	TATTTAAA	1567	8	+	Arabidopsis thaliana	core promoter element around -30 of transcription start
HbPAL8	TATA-box	ATTATA	1588	6	+	Brassica napus	core promoter element around -30 of transcription start
HbPAL8	TATA-box	TATAA	1589	5	-	Arabidopsis thaliana	core promoter element around -30 of transcription start

HbPAL8	TATA-box	TATA	1590	4	-	Arabidopsis thaliana	core promoter element around -30 of transcription start
HbPAL8	TATA-box	TATAAAA	1608	7	-	Pisum sativum	core promoter element around -30 of transcription start
HbPAL8	TATA-box	TATAAA	1609	6	-	Helianthus annuus	core promoter element around -30 of transcription start
HbPAL8	TATA-box	TATAA	1610	5	-	Arabidopsis thaliana	core promoter element around -30 of transcription start
HbPAL8	TATA-box	TATA	1611	4	-	Arabidopsis thaliana	core promoter element around -30 of transcription start
HbPAL8	TATA-box	ATATAT	1662	6	-	Brassica napus	core promoter element around -30 of transcription start
HbPAL8	TATA-box	TATA	1663	4	-	Arabidopsis thaliana	core promoter element around -30 of transcription start
HbPAL8	TATA-box	ATATAT	1778	6	-	Brassica napus	core promoter element around -30 of transcription start
HbPAL8	TATA-box	TATA	1779	4	-	Arabidopsis thaliana	core promoter element around -30 of transcription start
HbPAL8	TCA	TCATCTTCAT	364	9	-	Pisum sativum	
HbPAL8	TCA	TCATCTTCAT	385	9	-	Pisum sativum	
HbPAL8	TCA	TCATCTTCAT	391	9	-	Pisum sativum	
HbPAL8	TC-rich repeats	ATTCTCTAAC	1932	9	-	Nicotiana tabacum	cis-acting element involved in defense and stress responsiveness
HbPAL8	TCT-motif	TCTTAC	1168	6	+	Arabidopsis thaliana	part of a light responsive element
HbPAL8	W box	TTGACC	1342	6	+	Arabidopsis thaliana	WRKY binding site

Table S3. List of primer sequences used for qRT-PCR analysis.

Gene	Primer sequence (5'-3')	Primer length (bp)	PCR product size (bp)	Primer efficiency
HbPAL1	F: TTGAGTCGTGTTGTGTGGTATC	22	86	1.06
	R: AGAAGGTGGGAATGGGGTTT	20		
HbPAL2	F: TGAAGCACGCAGTCAAAAAC	20	101	0.98
	R: GTCCTTCTCGCAGAACCTTG	20		
HbPAL3	F: CACGCCTCTGCCAATTTGTTA	21	107	0.93
	R: GGGAAATGCAGAATGCTCTTACA	23		
HbPAL4	F: GAATCGTCGAGAGGTGGTGT	20	128	0.95
	R: GCTCCTCGGTTGGTTCTTCT	20		
HbPAL5	F: GGAATACGTGTTCGCCTACG	20	171	0.96
	R: AGGGCCTTGAGTTCCTCTTC	20		
HbPAL6	F: AAGTTTGCTAACCGGCGAGA	20	143	0.95
	R: CTTAGCTGATAGGAAGAGGAGCA	23		
HbPAL7	F: ATGTCCGCGACTTTCATGGT	20	113	0.99
	R: AGGGTTTTCCTTACCGCCTG	20		
HbPAL8	F: GTGACCGAGTAGCGAAGAGC	20	90	0.95
	R: TGGCACAAAATCCAGTGGTA	20		
HbUBC4	F: TACAAAGAGGTGCAGCGTGA	20	125	0.96
	R: ACTCCGCCCTCATAAGGAGT	20		
